# Design, synthesis, *in silico* studies, and apoptotic antiproliferative activity of novel thiazole-2-acetamide derivatives as tubulin polymerization inhibitors

**DOI:** 10.3389/fchem.2025.1565699

**Published:** 2025-04-16

**Authors:** Lamya H. Al-Wahaibi, Ali M. Elshamsy, Taha F. S. Ali, Bahaa G. M. Youssif, Stefan Bräse, Mohamed Abdel-Aziz, Nawal A. El-Koussi

**Affiliations:** ^1^ Department of Chemistry, College of Sciences, Princess Nourah bint Abdulrahman University, Riyadh, Saudi Arabia; ^2^ Pharmceutical Chemistry Department, Faculty of Pharmacy, Deraya University, Minia, Egypt; ^3^ Medicinal Chemistry Department, Faculty of Pharmacy, Minia University, Minia, Egypt; ^4^ Department of Pharmaceutical Organic Chemistry, Faculty of Pharmacy, Assiut University, Assiut, Egypt; ^5^ Institute of Biological and Chemical Systems, IBCS-FMS, Karlsruhe Institute of Technology, Karlsruhe, Germany; ^6^ Department of Pharmaceutical Medicinal Chemistry, Faculty of Pharmacy, Assiut University, Assiut, Egypt

**Keywords:** tubulin, colchicine, CA-4, antiproliferative, cell viability, docking

## Abstract

**Introduction:**

Tubulin polymerization inhibitors have emerged as interesting anticancer therapies. We present the design, synthesis, and structural elucidation of novel thiazole-based derivatives to identify novel tubulin inhibitors with potent antiproliferative efficacy and strong inhibition of tubulin polymerization.

**Methods:**

The novel compounds consist of two scaffolds. Scaffold A compounds **10a-e** and scaffold B compounds **13a-e**. the structures of the newly synthesized compounds **10a-e** and **13a-e** were validated using ^1^H NMR, ^13^C NMR, and elemental analysis.

**Results and Discussion:**

The most effective antitubulin derivative was **10a**, exhibiting an IC_50_ value of 2.69 μM. Subsequently, **10o** and **13d** exhibited IC_50_ values of 3.62 μM and 3.68 μM, respectively. These compounds exhibited more potency than the reference combretastatin A-4, which displayed an IC_50_ value of 8.33 μM. These compounds had no cytotoxic effects on normal cells, preserving over 85% cell viability at 50 μM. The antiproliferative experiment demonstrated that compounds **10a**, **10o**, and **13d** displayed significant activity against four cancer cell lines, with average GI_50_ values of 6, 7, and 8 μM, equivalent to the reference’s doxorubicin and sorafenib. Compounds 10a, 10o, and 13d were demonstrated to activate caspases 3, 9, and Bax, while down-regulating the anti-apoptotic protein Bcl2. Molecular docking studies demonstrated superior binding affinities for **10a** (-7.3 kcal/mol) at the colchicine binding site of tubulin, forming key hydrophobic and hydrogen bonding interactions that enhance its activity. ADMET analysis confirmed favorable drug-like properties, establishing these compounds as promising candidates for further development as anticancer agents targeting tubulin polymerization.

## 1 Introduction

Cancers are a group of disorders characterized by unregulated and disorganized cellular proliferation. These disorders can spread to nearby tissues through a process called metastasis, which is the main cause of cancer-related death (El-Sherief et al., 2018; Hagar et al., 2023; Chu and Mehrzad, 2023). Factors associated with an elevated cancer risk include tobacco consumption, insufficient physical exercise, alcohol consumption, inadequate intake of fruits and vegetables, and obesity. These factors are believed to contribute to roughly one-third of cancer deaths (Seiler et al., 2018; Sun et al., 2020; Gregory et al., 2024). Cancer development entails many genetic changes in aberrant cell proliferation (Madhurya et al., 2024; Hanahan and Weinberg, 2011). The rising global cancer incidence has resulted in significant advancements in the identification of novel, safer, and curative chemotherapeutic drugs (Anand et al., 2023). In prominently developing new antitumor drugs, we focus on the current study of thiazoles, which are bioactive heterocyclic compounds known for their numerous biological activities and have prominent anticancer properties (Sahil et al., 2022; Kassem et al., 2024; Rana et al., 2023; Sabry et al., 2022; Wang et al., 2025).

**FIGURE 1:**
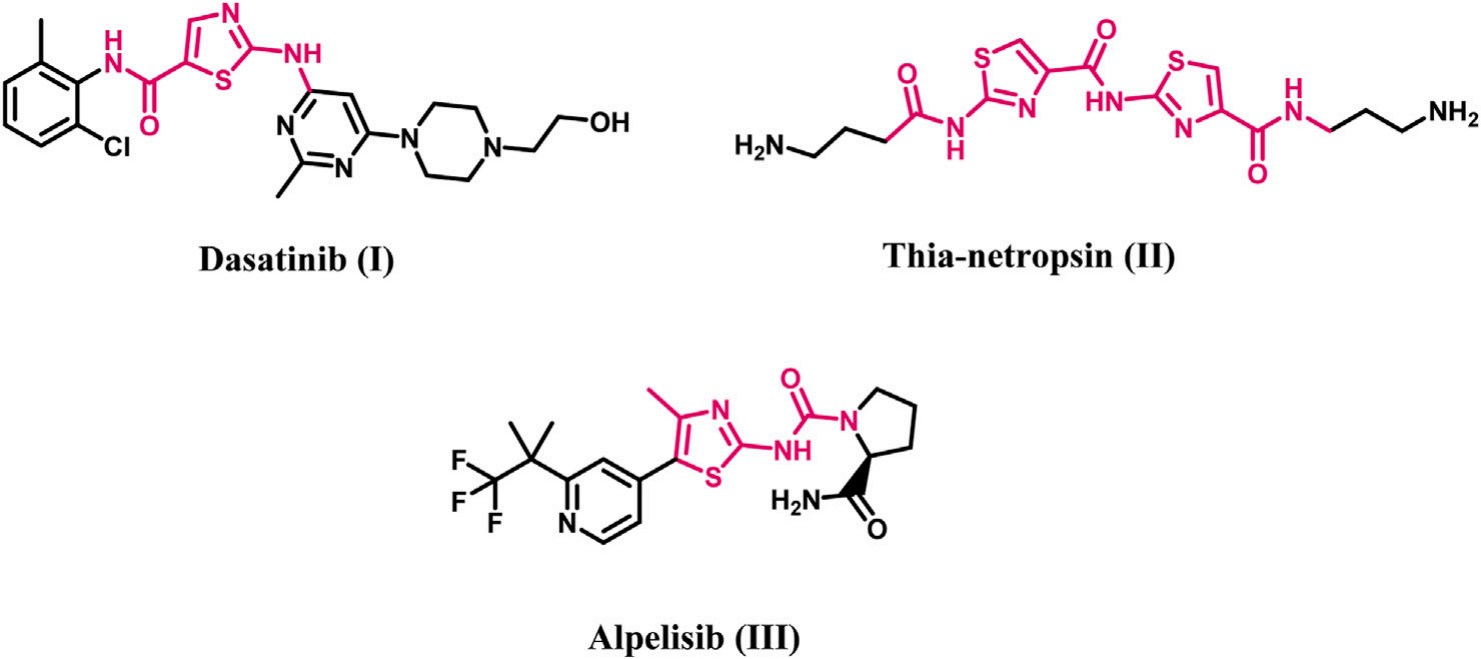
Structures of Dasatinib, thia-netropsin, and Alpelisib.

Thiazole derivatives featuring either one or more thiazole rings constitute a significant class of heterocyclic compounds recognized for their anticancer properties, attributed to their notable affinity for various biological targets implicated in cancer development, including tiazofurin (Tricot et al., 1990; Franchetti et al., 1995) and bleomycin (Hecht, 2000). Efforts have been made to improve the antitumor activity of the 2-aminothiazole core in anticancer therapies, such as dasatinib (**I**, Figure 1; Saha et al., 2016), thia-netropsin (**II**, Figure 1; Plouvier et al., 1989), and alpelisib (**III**, Figure 1; Juric et al., 2019), which received medical approval in 2019.

**FIGURE 2:**
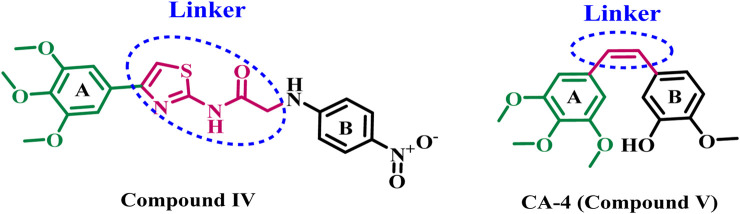
Structures of compound **IV** and CA-4 **(V)**.

Additionally, studies have shown that several 2-amino-4-phenylthiazol derivatives hinder tubulin polymerization, interfere with microtubule assembly, and impair cellular division (Lee et al., 2010; Sun et al., 2017). A novel series of 2,4-disubstituted thiazole derivatives was developed and assessed for their potential anticancer action as tubulin polymerization inhibitors (El-Abd et al., 2022). All synthesized compounds were evaluated for their cytotoxic activities against four human cancer cell lines. The results indicated that compound **IV** (Figure 2) was the most effective inhibitor of tubulin polymerization, with an IC_50_ value of 2.00 ± 0.12 μM, surpassing that of the reference compound combretastatin A-4 (CA-4) (IC_50_ = 2.96 ± 0.18 μM). The docking analysis of compound **IV** into the colchicine binding site indicated that both the sulfur atom of the thiazole ring and the amidic NH established hydrogen bonds with the residue ThrB353, which is crucial for receptor site interaction. Additionally, the amidic NH formed a hydrogen bond with the GlnB247 amino acid residue, demonstrating the importance of this group (amidic NH) for activity.

Microtubules are long, filamentous, tubular protein polymers that help cells form and maintain their structure. They help with tasks like transporting vesicles and protein complexes, maintaining cell shape, and directing cell migration and division (Gudimchuk and McIntosh, 2021; Logan and Menko, 2019). Microtubules comprising tubulin heterodimers are very important in mitosis, marked by increased dynamic instability during spindle formation and chromosome segregation (Vicente and Wordeman, 2015). Disruption of microtubules can induce cell cycle arrest in the G2-M phase and produce abnormal mitotic spindles. Because of their role in mitosis and cell division, microtubules are an attractive target for anticancer drug development.

The South African tree Combretum caffrum naturally generates Combretastatin A4 (**CA-4**, compound **V**, Figure 2), a stilbene that inhibits tubulin polymerization and exhibits significant cytotoxicity to a variety of human cancer cell lines. Furthermore, investigations have shown that CA-4 inhibits the blood flow to cancer cells. Nonetheless, the isomerization of the cis-double bond to a more stable and inactive trans-form impeded its potential therapeutic application (Tron et al., 2006; Seddigi et al., 2017). As a result, researchers are increasingly interested in developing novel tubulin inhibitors for cancer treatment.

### 1.1 Rational design

El-Abd et al. (2022) have reported the development of a new series of thiazole-based tubulin inhibitors. Compound **IV**, depicted in Figure 2, was the most potent tubulin inhibitor. In this series, the authors maintain the trimethoxy phenyl moiety (ring A) in CA-4 while substituting the cis-alkene linker in CA-4 with a thiazole-2-acetamide moiety as a rigid heterocyclic linker. Diversity in structure was achieved by including various bioactive side arms at the 2-position of the chosen backbone (Ring B), specifically amide derivatives, urea derivatives, or other heterocyclic moieties, including thiazole.

Gaspari et al. (2017) present a high-resolution crystal structure of the tubulin-cis-CA-4 complex. Researchers discovered that CA-4 interacts with the tubulin site and is distinct from the standard microtubule-destabilizing drug colchicine. The 3,4,5-trimethoxy-substituted phenyl (ring A) is deeply embedded in the tubulin-cis-CA-4 complex via a network of hydrophobic interactions with the amino acid residues that define the receptor site’s hydrophobic pocket. Hydrophobic interactions with various amino acid residues that define the pocket help to stabilize the 3-hydroxy-4-methoxy-substituted phenyl moiety (ring B) of cis-CA-4. The hydroxyl group in ring B also establishes two hydrogen bonds with the Val181 and Thr179 residues.

**FIGURE 3:**
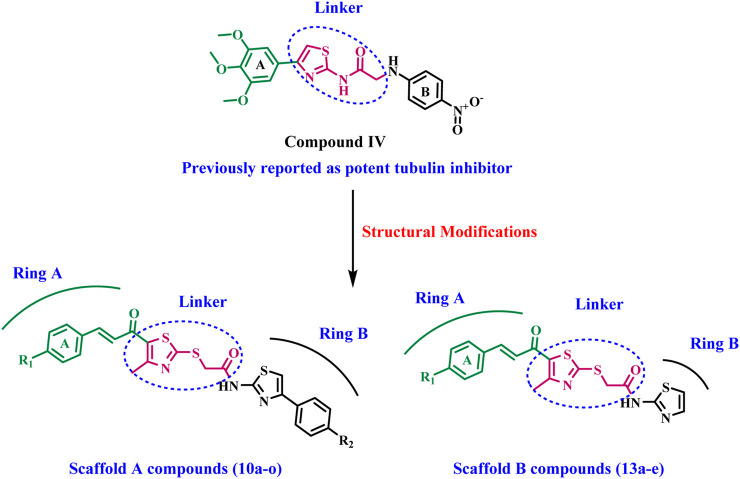
Structures of previously reported compound **IV** and new compounds **10a-o** and **13a-e**.

Inspired by the previously mentioned data, we continue our initiative (Abdelbaset et al., 2021; Abdelbaset et al., 2018) to develop specific anticancer agents as tubulin inhibitors by designing and synthesizing a novel series of thiazole-based inhibitors. The newly synthesized compounds comprise three major components: In ring A, we replace the tri-methoxy phenyl group in compound **IV** with a chalcone moiety, seeking to improve binding to the colchicine binding site through an array of hydrophobic interactions. The second component is the linker, the thiazole-2-acetamide moiety, as seen in compound **IV**. The final component is ring B, which is the thiazole or 4-phenyl thiazole moiety responsible for both hydrophobic and hydrophilic interactions. As shown in Figure 3.

The newly synthesized compounds are of two scaffolds: Scaffold A, which contains compounds **10a–o**, and Scaffold B, which comprises compounds **13a–e**. All newly synthesized compounds were tested for their tubulin inhibitory activity using CA-4 as a reference compound. Next, the most potent tubulin inhibitors were tested for their antiproliferative activity against a panel of four cancer cell lines. Finally, a docking study was performed to explore the binding method and interactions within the colchicine binding site.

## 2 Results and discussion

### 2.1 Chemistry

**SCHEME 1:**
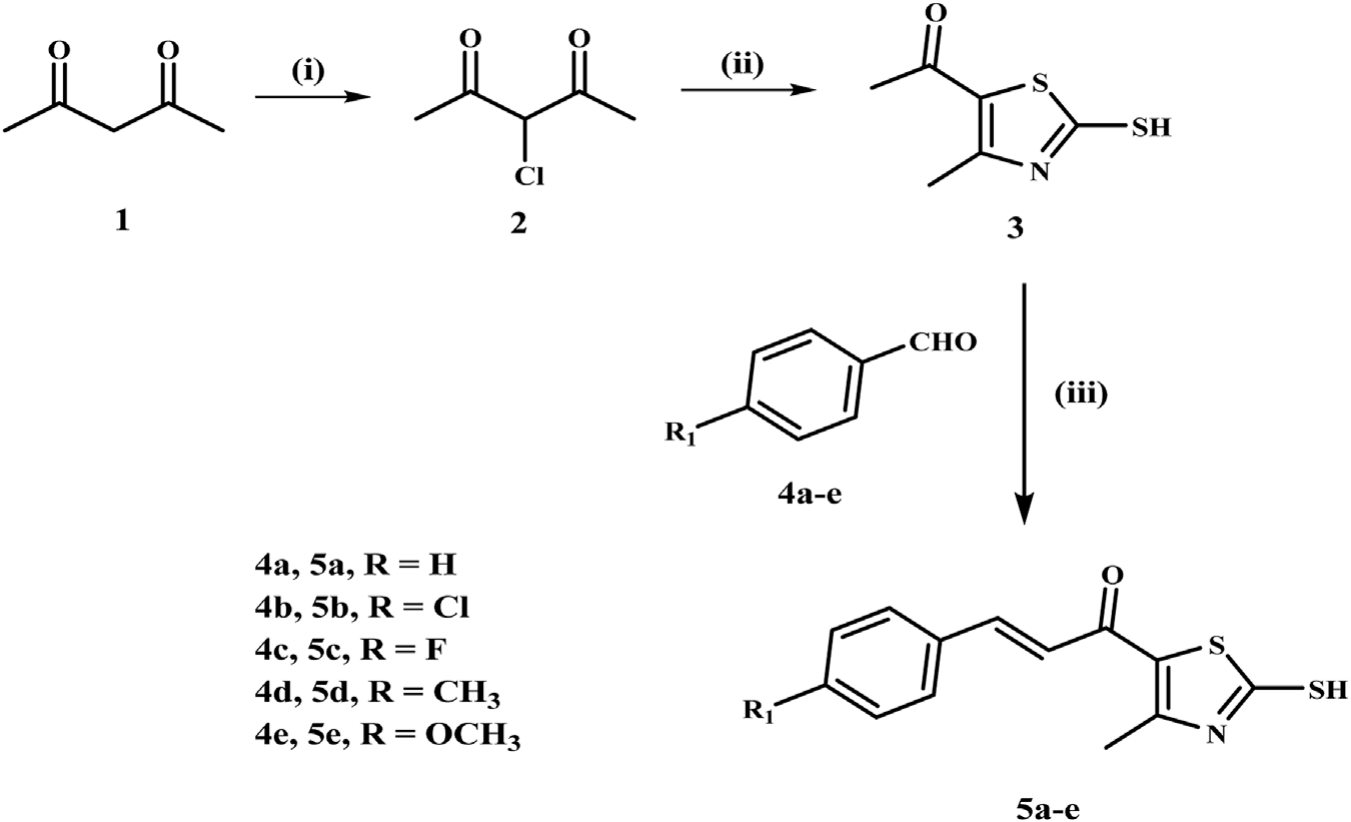
Synthesis of the chalcone intermediates** 5a–e**. Reagents and Reaction conditions: (i) SO_2_Cl_2_, toluene, 0°C, 12 h; (ii) NH_3_, CS_2_, EtOH, RT, 6 h; (iii) appropriate aromatic aldehyde, 60% NaOH, EtOH, 0°C, 18 h.

The synthesis of chalcone intermediates **5a-e** employs a multi-step pathways, as illustrated in Scheme 1, which begins with the synthesis of 3-chloroacetylacetone **(2)** from acetylacetone **(1)** via chlorination using sulfuryl chloride in toluene at 0°C. This reaction chlorinates the methylene group, generating the reactive electrophilic intermediate (Abo-Ashour et al., 2018). Compound **2** is then cyclized into 1-(2-mercapto-4-methylthiazol-5-yl)ethan-1-one **(3)** reacting with ammonia and carbon disulfide in absolute ethanol. The acetyl group of the thiazole ring then undergoes a Claisen-Schmidt condensation reaction with different substituted benzaldehydes **(4a-e)** in ethanol under basic conditions, resulting in chalcone derivatives **(5a-e)** with the development of a new carbon-carbon double bond (Hashem et al., 2024).

**SCHEME 2:**
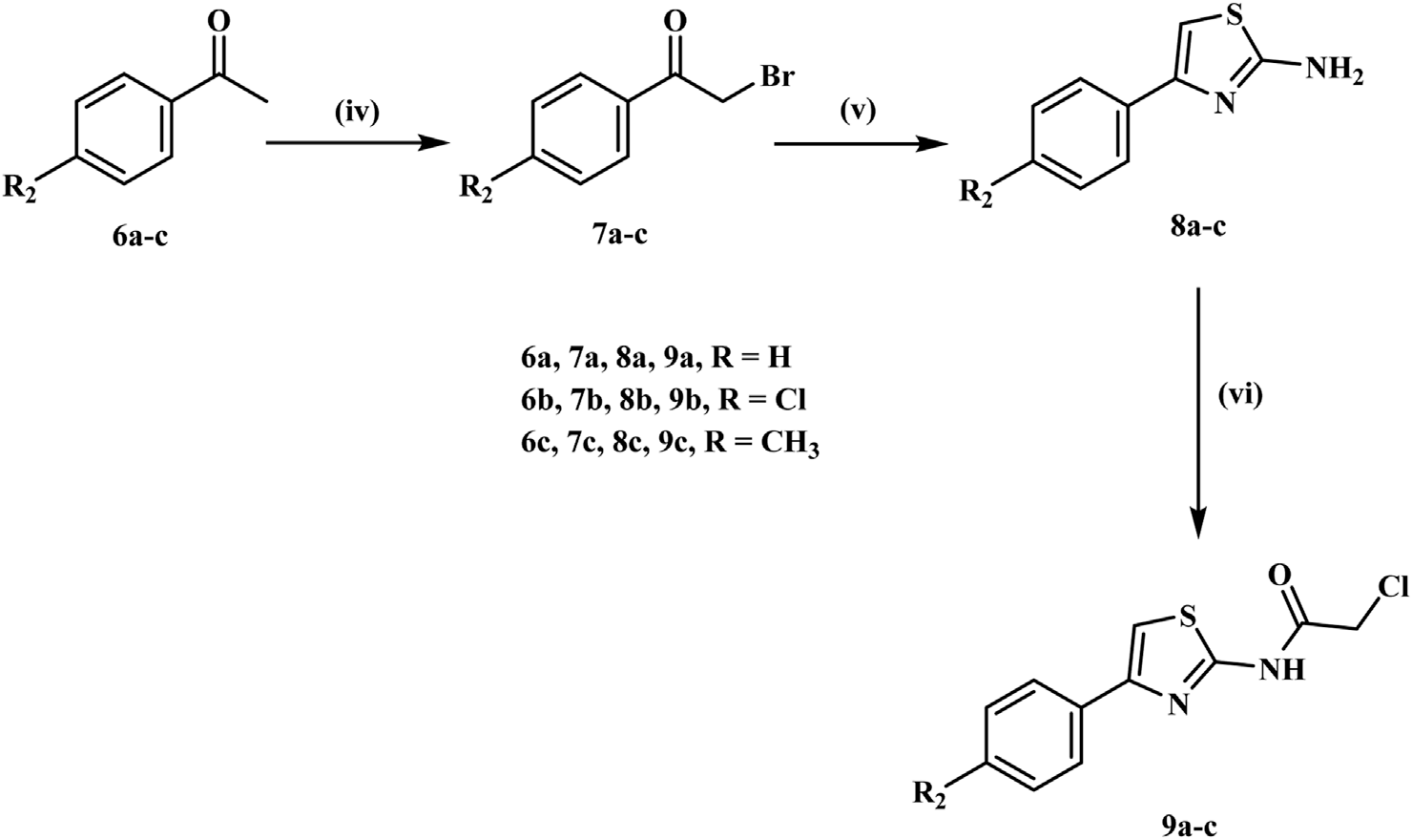
Synthesis of the key intermediates **9a-c**. Reagents and Reaction conditions: (iv) NBS, PTSA. H_2_O, acetonitrile, reflux, 10 h; (v) thiourea, Na_2_CO_3_, EtOH, reflux, 5 h; (vi) Chloroacetyl chloride, DCM, Na_2_CO_3_, H_2_O, 0°C, 12 h.

Simultaneously, phenacyl bromides **(7a-c)** are synthesized from acetophenone derivatives **(6a-c)** via α-bromination using N-bromo succinimide (NBS) with *p*-toluene sulfonic acid as a catalyst in refluxing acetonitrile, as illustrated in Scheme 2. Phenacyl bromides **(7a-c)** react with thiourea in ethanol under reflux to yield 2-amino-4-aryl-thiazoles **(8a-c)**, which are subsequently acylated with chloroacetyl chloride in dichloromethane using sodium carbonate as a base, resulting in *N*-acylated thiazoles **(9a-c)** containing an electrophilic chloroacetyl moiety (Valiveti et al., 2015).

**SCHEME 3:**
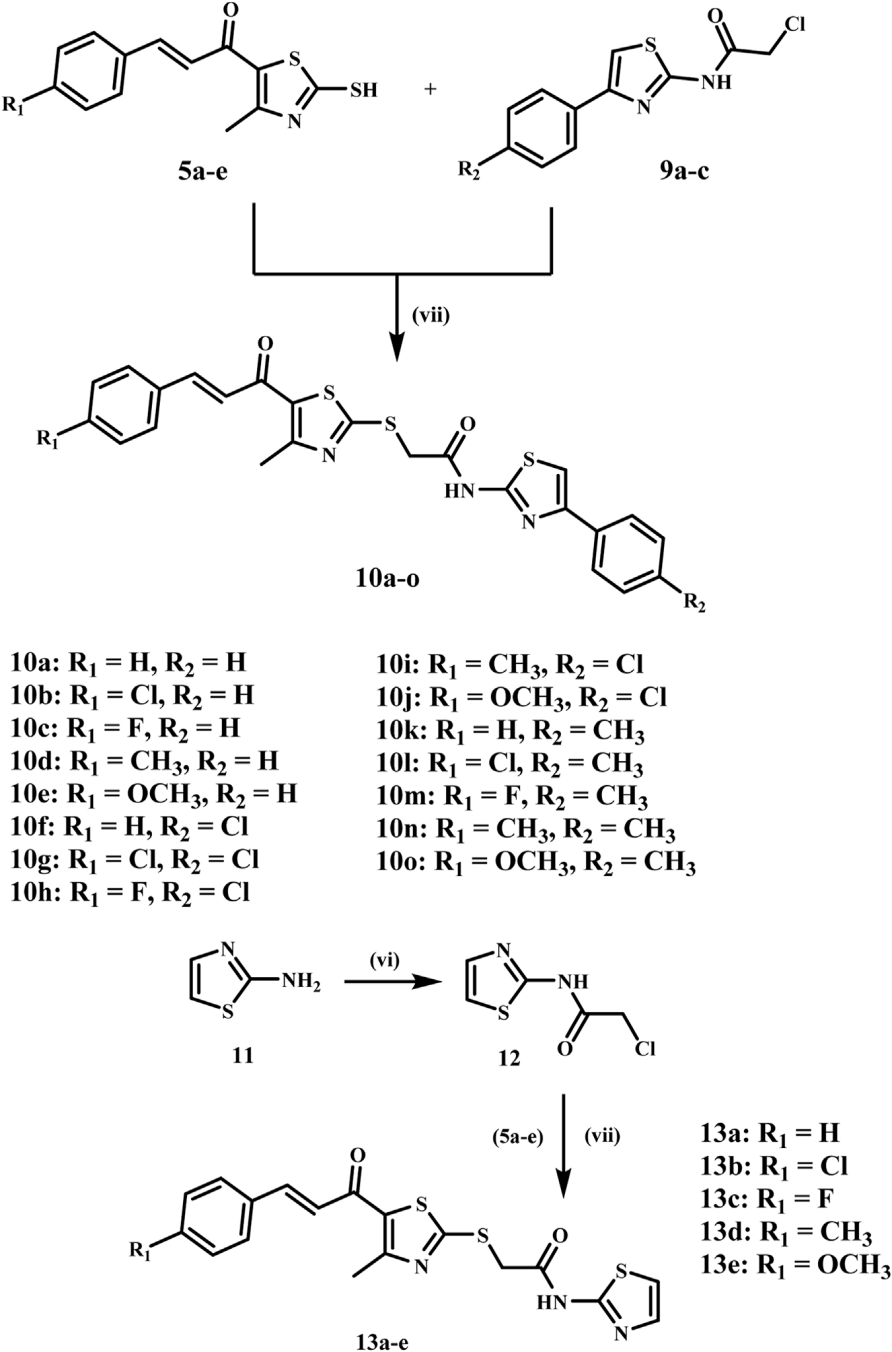
Synthesis of the target compounds **10a-o** and **13a-e**. Reagents and Reaction conditions: (vi) Chloroacetyl chloride, DCM, Na_2_CO_3_, H_2_O, 0°C, 12 h; (vii) Na_2_CO_3_, NaI, acetone, RT, 6 h.

The final step comprises alkylation of the chalcone derivatives **5a-e** with the acylated thiazoles **9a-c** in acetone using anhydrous sodium carbonate and sodium iodide to afford the target compounds **10a-o**, which were isolated in good yields after recrystallization from ethanol, Scheme 3. The structures of the final compounds **10a-o** were elucidated by ^1^H NMR, ^13^C NMR, and elemental analysis. The ^1^H NMR spectra of the target compounds **10a-o** showed distinct peaks, including a singlet peak at δ 4.40–4.44 ppm, which indicates the methylene group. The methyl group on the thiazole ring displays a singlet signal at δ 2.64–2.66 ppm, whereas the chalcone protons provide two doublets at around δ 7.30 and 7.65 ppm. In addition, compounds having methoxy groups show a characteristic peak at around δ 3.81–3.82 ppm. The NH of the amide group displayed a singlet at δ 12.67–12.74 ppm, thereby corroborating the structure of these compounds. Compound **10j** is a good example of this group because it had characteristic peaks in ^1^H NMR, like two singlets at δ 2.65 and 4.41 ppm, which are the methyl and methylene groups. The methoxy group appeared as a singlet peak at δ 3.81 ppm, whereas the typical two doublets of the chalcone protons appeared at δ 7.23 and 7.65 ppm, respectively. Additionally, the NH of the amide group exhibited a singlet signal at δ 12.74 ppm. The ^13^C NMR spectrum of **10j** detected the methyl group on the thiazole ring at δ 18.72 ppm and the methylene linker at δ 37.37 ppm. In addition, the carbonyl carbon signal was observed at δ 181.85 ppm and the methoxy group signal at 55.89 ppm.

Similarly, we synthesized scaffold B compounds **13a-e** by reacting 2-aminothiazole **11** with chloroacetyl chloride, which produced an intermediate **12**, Scheme 3 (Tilekar et al., 2020). The intermediate **12** then reacts with thiazole chalcone derivatives **5a-e** to form the target compound **13a-e** in high yields. ^1^H NMR exhibited a comparable pattern, with the methylene protons appearing as a singlet signal at δ 4.40–4.41 ppm and the methyl protons displaying a singlet signal at δ 2.64–2.66 ppm. Chalcone protons exhibited two doublets at δ 7.23–7.39 and 7.65–7.68, and the NH of the amide group appeared as a singlet at δ 12.54–12.55 ppm. Compound **13d**, as a representative example, exhibited three singlets at δ 2.35, 2.65, and 4.40 ppm, corresponding to the two methyl groups and the methylene group, respectively. The chalcone protons exhibit doublets at δ 7.31 and 7.65 ppm, while the NH of the amidic group showed a singlet signal at δ 12.54 ppm.

### 2.2 Biology

#### 2.2.1 Cell viability assay

The impact of novel compounds **10a-o** and **13a-e** on cell viability was examined using the MCF-10A (human mammary gland epithelial) normal cell line. The MTT test was employed to assess the cell viability of the novel compounds after a four-day incubation with MCF-10A cells (El-Sherief et al., 2019; Ramadan et al., 2020). Table 1 indicates that none of the analyzed compounds exhibited cytotoxic effects on normal cells; all compounds preserved cell viability over 85% at a concentration of 50 µM.

**TABLE 1:** Results of cell viability assay and anti-tubulin (IC_50_) for compounds **10a-o** and **13a-e**.

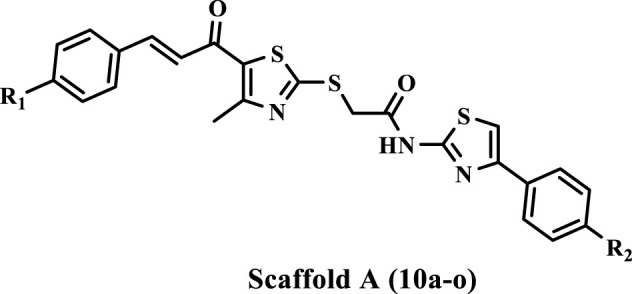 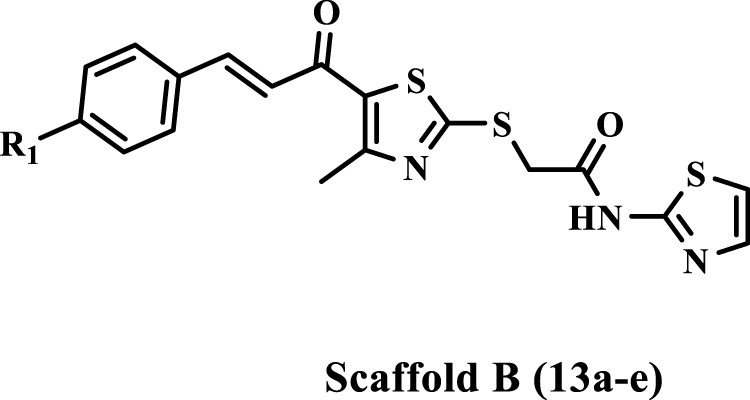				
Compound	R_1_	R_2_	Cell viability %	Tubulin‡ inhibition IC_50_ ± SEM (µM)
**10a**	H	H	87	2.69 ± 0.08
**10b**	Cl	H	91	8.75 ± 0.27
**10c**	F	H	89	22.50 ± 0.69
**10d**	Me	H	90	5.26 ± 0.16
**10e**	OMe	H	92	15.30 ± 0.49
**10f**	H	Cl	86	5.62 ± 0.17
**10g**	Cl	Cl	89	59.37 ± 3.78
**10h**	F	Cl	85	7.95 ± 0.24
**10i**	Me	Cl	91	33.45 ± 1.21
**10j**	OMe	Cl	93	20.55 ± 0.75
**10k**	H	Me	90	47.75 ± 1.74
**10l**	Cl	Me	89	13.93 ± 0.51
**10m**	F	Me	87	7.92 ± 0.29
**10n**	Me	Me	89	15.96 ± 0.59
**10o**	OMe	Me	90	3.62 ± 0.11
**13a**	H	--	89	15.82 ± 0.48
**13b**	Cl	--	87	3.73 ± 0.11
**13c**	F	--	86	6.05 ± 0.18
**13d**	Me	--	90	3.68 ± 0.14
**13e**	OMe	--	91	39.93 ± 1.22
CA-4	--	--	--	8.33 ± 0.29

--: Not applicable.

‡: Triplicate for each tested concentration.

#### 2.2.2 Tubulin inhibitory assay

The effects of all novel synthetic compounds **10a-o** and **13a-e** on tubulin polymerization, using CA-4 as a reference compound (Abdelbaset et al., 2021; Abdelbaset et al., 2018), are detailed in Table 1. Compounds **10a-o** and **13a-e** displayed strong anti-tubulin activity, with IC_50_ values ranging from 2.69 to 59.37 µM, compared to the reference CA-4 (IC_50_ = 8.33 µM). Compounds **10a**, **10d**, **10f**, **10h**, **10m**, **10o**, and **13b-d** had the most significant anti-tubulin activity, with IC_50_ values between 2.69 and 7.95 µM, surpassing the reference compound CA-4 (IC_50_ = 8.33 µM).

Compound **10a** (R_1_ = R_2_ = H, Scaffold A) outperformed all other evaluated compounds, demonstrating an IC_50_ of 2.69 µM, making it three times more effective than CA-4’s IC_50_ of 8.33 µM, as indicated in Table 1. The anti-tubulin efficacy of compounds **10a-o** is significantly affected by the substitution pattern on the chalcone moiety’s phenyl groups and at the thiazole moiety’s fourth position. For example, compounds **10b** (R_1_ = Cl; R_2_ = H), **10c** (R_1_ = F; R_2_ = H), **10d** (R_1_ = CH_3_; R_2_ = H), and **10e** (R_1_ = OCH_3_; R_2_ = H), which possess a phenyl group at the four positions of the thiazole moiety, demonstrated diminished efficacy as antitubulin agents relative to **10a** (R_1_ = R_2_ = H).

Compounds **10b**, **10c**, **10d**, and **10e** exhibit IC_50_ values of 8.75, 22.50, 5.26, and 15.30 µM, respectively, demonstrating that the unsubstituted phenyl group of the chalcone moiety is more favorable for anti-tubulin activity than the substituted phenyl group, with efficacy increasing in the sequence: H > CH_3_ > Cl > OCH_3_ > F. Compound **10d** exhibited superior potency compared to the standard CA-4.

Furthermore, substituting the hydrogen atom of the phenyl group at the four positions of the thiazole moiety in compound **10a** with a 4-chloro atom in compound **10f** (R_1_ = H, R_2_ = 4-Cl) or with a methyl group in compound **10k** (R_1_ = H, R_2_ = 4-CH_3_) resulted in a significant decrease in anti-tubulin activity. The IC_50_ values for **10f** and **10k** were 5.62 µM and 47.75 µM, respectively, indicating a potency decrease of 2-fold and 18-fold relative to **10a** (IC_50_ = 2.69 µM). These findings indicated that hydrogen and chlorine atoms at the phenyl group of the fourth position of the thiazole moiety are more tolerated than the methyl group for anti-tubulin activity.

Compound **10o** (R_1_ = OCH_3_, R_2_ = CH_3_) exhibited the second highest anti-tubulin activity, with an IC_50_ value of 3.62 µM, which is 1.4-fold less effective than **10a** (IC_50_ = 2.69 µM). Yet, **10o** demonstrated superior activity (2.4-fold) compared to CA-4 (IC_50_ = 8.33 µM), as indicated in Table 1. Ultimately, compound **10g** (R_1_ = R_2_ = Cl) exhibited the lowest potency among the anti-tubulin derivatives, with an IC_50_ value of 59.37 µM, rendering it 22-fold less potent than **10a** and 7-fold less potent than the reference CA-4, indicating that the incorporation of chlorine atoms on both phenyl groups is not conducive to activity. This supports the notion that the unsubstituted phenyl rings of the chalcone and thiazole moieties exhibited greater tolerance for activity.

The anti-tubulin activity of Scaffold B compounds, **13a-e**, was moderate to high relative to Scaffold A compounds, **10a-o**. Compounds **13a-e** exhibited enhanced IC_50_ values between 3.68 and 6.05 µM, with the exceptions of **13a** and **13e**, which displayed IC_50_ values of 15.82 and 39.93 µM, respectively. Compounds **13b**, **13c**, and **13d** exhibited the highest efficacy among scaffold B derivatives, with IC_50_ values of 3.73, 6.05, and 3.68 µM, respectively, rendering them more effective than the reference CA-4 (IC_50_ = 8.33 µM), but less potent than compound **10a** (IC_50_ = 2.69 µM). Compound **13a** (R_1_ = H) has the same structure as **10a**, but it does not have the phenyl group at the fourth position of the thiazole moiety. Its IC_50_ value is 15.82 µM, 6 times lower than **10a**′s. The observations underscore the phenyl group’s importance at the thiazole moiety’s fourth position for anti-tubulin action.

#### 2.2.3 Antiproliferative assay

The antiproliferative efficacy of novel compounds **10a**, **10d**, **10f**, **10h**, **10m**, **10o** (Scaffold A) and **13b-d** (Scaffold B) against four human cancer cell lines (colon - HCT-116, prostatic - PC-3, breast - MCF-7, and pancreatic - MDAMB-231) was assessed utilizing the MTT test (Mahmoud et al., 2022; Hisham et al., 2022; Al-Wahaibi et al., 2023). Doxorubicin and Sorafenib were employed as controls in this investigation. Table 2 displays the median inhibitory concentration (IC_50_) and GI_50_ (mean IC_50_) values for the four cancer cell lines.

**TABLE 2:** Antiproliferative activity (IC_50_) for compounds **10a-o** and **13a-e**.

Compd	Antiproliferative activity IC_50_ ± SEM (µM)				
	HCT-116	MCF-7	MDAMB-231	PC-3	GI_50_ (average IC_50_)
**10a**	8 ± 0.7	4 ± 0.2	6 ± 0.3	7 ± 0.6	6
**10d**	19 ± 1.5	10 ± 0.8	8 ± 0.7	16 ± 1.3	13
**10f**	25 ± 1.8	14 ± 1.1	10 ± 0.9	22 ± 1.6	18
**10h**	29 ± 1.9	24 ± 1.7	15 ± 1.3	61 ± 3.5	32
**10m**	35 ± 2.2	18 ± 1.4	20 ± 1.6	43 ± 2.5	29
**10o**	6 ± 0.4	5 ± 0.4	3 ± 0.1	13 ± 1.0	7
**13b**	17 ± 1.4	9 ± 0.7	8 ± 0.7	14 ± 1.2	12
**13c**	18 ± 1.4	28 ± 1.8	23 ± 1.7	34 ± 2.1	26
**13d**	9 ± 0.9	7 ± 0.4	5 ± 0.3	9 ± 0.8	8
Doxorubicin	5 ± 0.3	4 ± 0.2	3 ± 0.1	9 ± 0.6	5
Sorafenib	6 ± 0.3	7 ± 0.3	8 ± 0.4	12 ± 0.9	8

The outcomes of this antiproliferative assay are congruent with the *in vitro* antitubulin assay findings. Compounds **10a** (R_1_ = R_2_ = H), **10o** (R_1_ = OCH_3_, R_2_ = CH_3_), and **13d** (R_1_ = CH_3_), the most potent anti-tubulin derivatives, exhibited the highest antiproliferative efficacy with GI_50_ values of 6, 7, and 8 μM, respectively, rendering them comparably potent to the reference drugs doxorubicin and sorafenib, which have GI_50_ values of 5 and 8 μM, respectively.

Compound **10a** (R_1_ = R_2_ = H) was the most effective among the derivatives tested against the prostate PC-3 cancer cell line, with an IC_50_ value of 7 ± 0.6 µM. It had more potency than doxorubicin and sorafenib. Compound **10a** demonstrated 1.7-fold higher efficacy than sorafenib against the PC-3 prostate cancer cell line, as shown in Table 2. Also, compound **10a** showed strong antiproliferative activity against the MCF-7 breast cancer cell line, with an IC_50_ value of 4 ± 0.2 µM, which was higher than other derivatives and equal to doxorubicin (IC_50_ = 4 ± 0.2 µM) and twice as strong as sorafenib (IC_50_ = 7 ± 0.3 µM).

Compound **10o** (R_1_ = OCH_3_, R_2_ = CH_3_), the second most efficient derivative in all assays, had the highest potency among the derivatives evaluated against the pancreatic - MDAMB-231 cancer cell line, with an IC_50_ value of 3 ± 0.2 µM. It exhibited equivalent potency to doxorubicin (IC_50_ = 3 ± 0.2 µM) but showed more potency than sorafenib. Compound **10o** demonstrated 2.7-fold more potency than sorafenib against the pancreatic MDAMB-231 cancer cell line, Table 2. Compound **10o** exhibited equal efficacy to doxorubicin against both colon (HCT-116) and breast (MCF-7) cancer cell lines. However, it was marginally less efficient than doxorubicin against the prostatic-PC-3 cancer cell line.

The third most potent compound, **13d** (R_1_ = CH_3_), had significant antiproliferative activity with IC_50_ values equivalent to sorafenib across all four cancer cell lines analyzed, but it was often somewhat less effective than doxorubicin, as illustrated in Table 2. Ultimately, all other evaluated derivatives **10d**, **10f**, **10h**, **10m**, **13b**, and **13c** exhibited weak to moderate antiproliferative activity, with GI_50_ values between 12 and 29 μM, demonstrating at least 2.5-fold and 1.5-fold reduced potency compared to doxorubicin and sorafenib, respectively.

#### 2.2.4 Apoptotic markers assay

Apoptosis, or anticipated cell death, encompasses various biochemical and morphological processes (Cavalcante et al., 2019). Antiapoptotic proteins, such as Bcl-2, coexist with proapoptotic proteins like Bax (Qian et al., 2022). Pro-apoptotic proteins facilitate the release of cytochrome c, whereas anti-apoptotic proteins modulate apoptosis by inhibiting cytochrome c release. The outer mitochondrial membrane becomes permeable when proapoptotic protein concentrations exceed those of antiapoptotic proteins, triggering a cascade of events. The release of cytochrome c initiates caspase-3 and caspase-9 activation. Caspase-3 causes apoptosis by targeting many critical proteins necessary for cellular function (Ponder and Boise, 2019).

Compounds **10a**, **10o**, and **13d**, the most efficient in all *in vitro* experiments, were further investigated as activators of apoptotic markers.

##### 2.2.4.1 Caspase 3/9 activation assay

Compounds **10a**, **10o**, and **13d** were assessed as activators of caspase-3/9 in the MDAMB-231 pancreatic cancer cell line (Youssif et al., 2019), with results detailed in Table 3. The results indicated that cells treated with derivatives **10a**, **10o**, and **13d** exhibited markedly elevated levels of caspase-3 protein (625 ± 5, 605 ± 5, and 690 ± 5 pg/mL, respectively), compared to the cells treated with standard compound staurosporine (510 ± 4 pg/mL). Compounds **10a**, **10o**, and **13g** induced an increase of the overall amount of caspase-3 protein in the MDAMB-231 cancer cell line, reaching levels almost 10-fold greater than the untreated control cells and surpassing those of the reference compound staurosporine.

**TABLE 3:** Caspase-3/9 activation of compounds **10a**, **10o**, and **13d** against MDAMB-231 pancreatic cancer cell line.

Compound number	Caspase-3		Caspase-9	
	Conc (pg/ml)	Fold change	Conc (ng/ml)	Fold change
**10a**	625 ± 5	10	1.60 ± 0.10	16
**10o**	605 ± 5	9	0.90 ± 0.10	9
**13d**	690 ± 5	11	1.50 ± 0.10	15
Staurosporine	510 ± 4	8	1.30 ± 0.10	13
Control	65 ± 0.5	1	0.10 ± 0.001	1

Additionally, caspase-9 activation testing results showed that test compounds **10a**, **10o**, and **13g** significantly increased caspase-9 levels compared to staurosporine. Compound **10a** exhibited the highest overexpression of caspase-9 (1.60 ng/mL), proceeded by compound **13d** (1.50 ng/mL) and staurosporine as a control (1.30 ng/mL). Compound **10o** was the least efficient derivative as caspase-9 activators (0.90 ng/mL). These data suggest that apoptosis may contribute to the antiproliferative actions of the examined compounds, possibly due to the activation of caspase-3 and 9.

##### 2.2.4.2 Assay for levels of bax and Bcl2

We additionally investigated the impact of compounds **10a**, **10o**, and **13d** on the apoptotic marker Bax and the anti-apoptotic Bcl2 levels in the MDAMB-231 cancer cell line, applying staurosporine as a reference (Youssif et al., 2019). The results are presented in Table 4.

**TABLE 4:** Bax and Bcl-2 levels of **10a, 10o** and **13d** in pancreatic MDAMB-231 cell line.

Compound number	Bax		Bcl-2	
	Conc (pg/ml)	Fold change	Conc (ng/ml)	Fold reduction
**10a**	305 ± 8	38	0.90	6
**10o**	285 ± 7	35	1.05	5
**13d**	320 ± 8	40	0.80	6
Staurosporine	280 ± 7	35	1.10	5
Control	8 ± 0.01	1	5	1

Compounds **10a**, **10o**, and **13d** exhibited superior Bax induction (305, 285, and 320 pg/mL, respectively) compared to staurosporine (280 pg/mL), reflecting a 38-fold, 35-fold, and 40-fold improvement relative to untreated control MDAMB-213 carcinoma cells. Ultimately, compound **13d** induced a significant reduction in the anti-apoptotic Bcl-2 protein concentration (0.80 ng/mL), got by compound **10a** (0.90 ng/mL) in the MDAMB-231 cell line, in contrast to staurosporine (1.10 ng/mL). These apoptosis experiments demonstrated that compounds **10a** and **13d** exhibited a significant apoptotic antiproliferative activity.

### 2.3 Computational studies

#### 2.3.1 Molecular docking study

**FIGURE 4:**
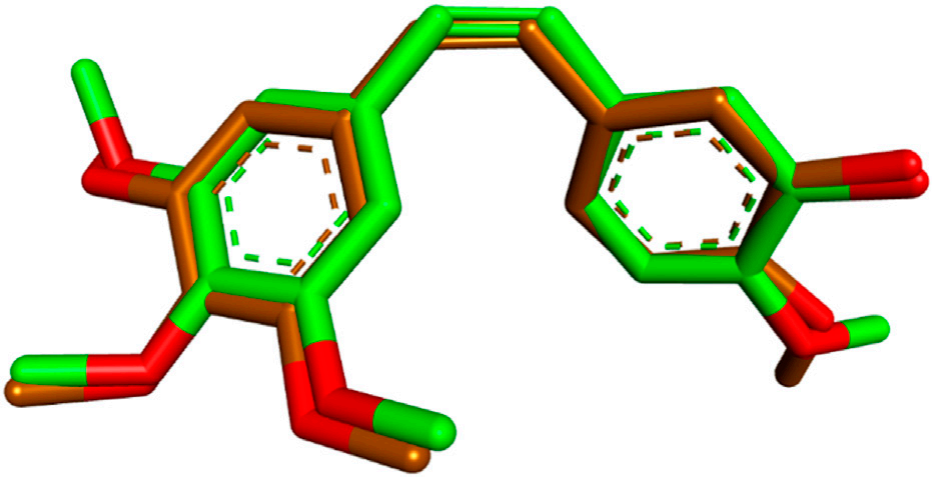
Superimposition of redocked (brown) and co-crystallized (green) poses of **CA-4** in the colchicine binding site (RMSD = 0.4994 Å).

The most potent compounds from Scaffold A (**10a**) and Scaffold B (**13d**) were selected for molecular docking studies to explore their interactions with the colchicine binding site of tubulin. The crystal structure of tubulin (PDB code: 5LYJ) was used for docking (Wang et al., 2024; Afzal et al., 2023), and the simulations were performed using Auto-Dock Vina (Trott and Olson, 2010). Visualization and analysis of the docking results were carried out using Discovery Studio Visualizer (Podila et al., 2024; Fatima et al., 2023), enabling a detailed understanding of the molecular interactions at the target site. To validate the docking protocol and ensure its reliability for subsequent docking studies, the co-crystallized ligand combretastatin A4 (**CA-4**) was redocked into the colchicine binding site of tubulin. The redocking yielded a low root-mean-square deviation (RMSD) of 0.4994 Å, indicating a high degree of alignment between the redocked and the original co-crystallized ligand. Additionally, the redocked ligand demonstrated a favorable binding affinity of −6.6 kcal/mol, consistent with its known interaction profile. The superimposition of the redocked and co-crystallized **CA-4** is illustrated in Figure 4.

**FIGURE 5:**
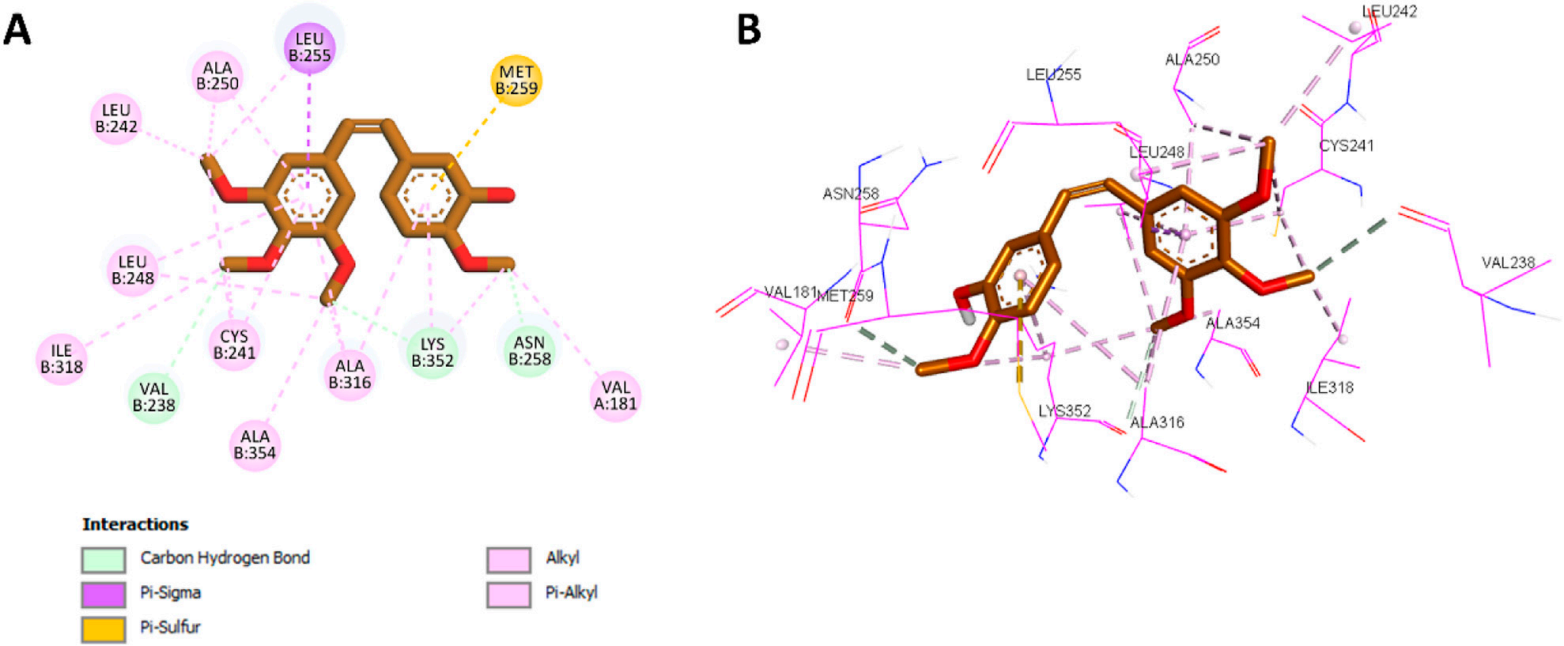
**(A)** 2D interaction diagram and **(B)** 3D binding mode of CA‐4 within the colchicine binding site.

After validating the docking protocol with **CA-4**, the most potent compound in Scaffold A, compound **10a**, was docked into the colchicine binding site of tubulin. Docking results showed that **10a** exhibited an improved binding affinity of −7.3 kcal/mol, surpassing the native ligand **CA-4**. Compared with the CA-4 binding mode (Figure 5), **10a** retained most of the key binding interactions demonstrated by CA-4 while introducing additional interactions that potentially account for its superior potency.

**FIGURE 6:**
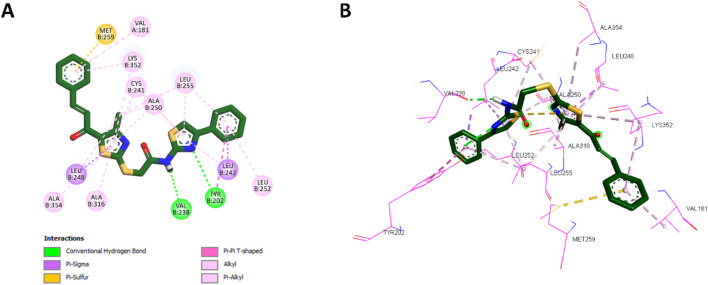
**(A)** 2D interaction diagram and **(B)** 3D binding mode of **10a** within the colchicine binding site.

Docking analysis revealed that **10a** was well-positioned within the hydrophobic pocket of the colchicine binding site. It preserved key hydrophobic interactions with residues Leu248, Ala354, Ala316, Cys241, Ala250, Leu255, and Leu242, which were also observed with **CA-4**. Furthermore, **10a** formed new hydrophobic interactions with Lys352 and additional hydrogen bonds with Tyr202, absent in the **CA-4** complex. The amide group of **10a** further contributed a novel hydrogen bond with Val238, another interaction not observed with **CA-4**. The phenyl ring of the chalcone scaffold in **10a** participated in hydrophobic interactions, including π-sulfur interactions with Met259 and π-alkyl interactions with Val181 and Lys352, which were also observed in the **CA-4** binding profile. Additionally, the phenyl ring attached to the thiazole moiety engaged in π-sigma interactions with Leu242 (a conserved interaction with **CA-4**) while introducing unique contacts such as π-alkyl interactions with Leu252 and π–π T-shaped interactions with Tyr202. These distinct interactions are illustrated in Figure 6, showing the 2D and 3D interaction profiles of **10a** within the colchicine binding site.

These docking results correlate strongly with *in vitro* findings, where **10a** exhibited greater inhibitory potency than **CA-4**. Its higher binding affinity, retention of key interactions, and novel hydrophobic and hydrogen bonding interactions collectively explain its enhanced activity.

**FIGURE 7:**
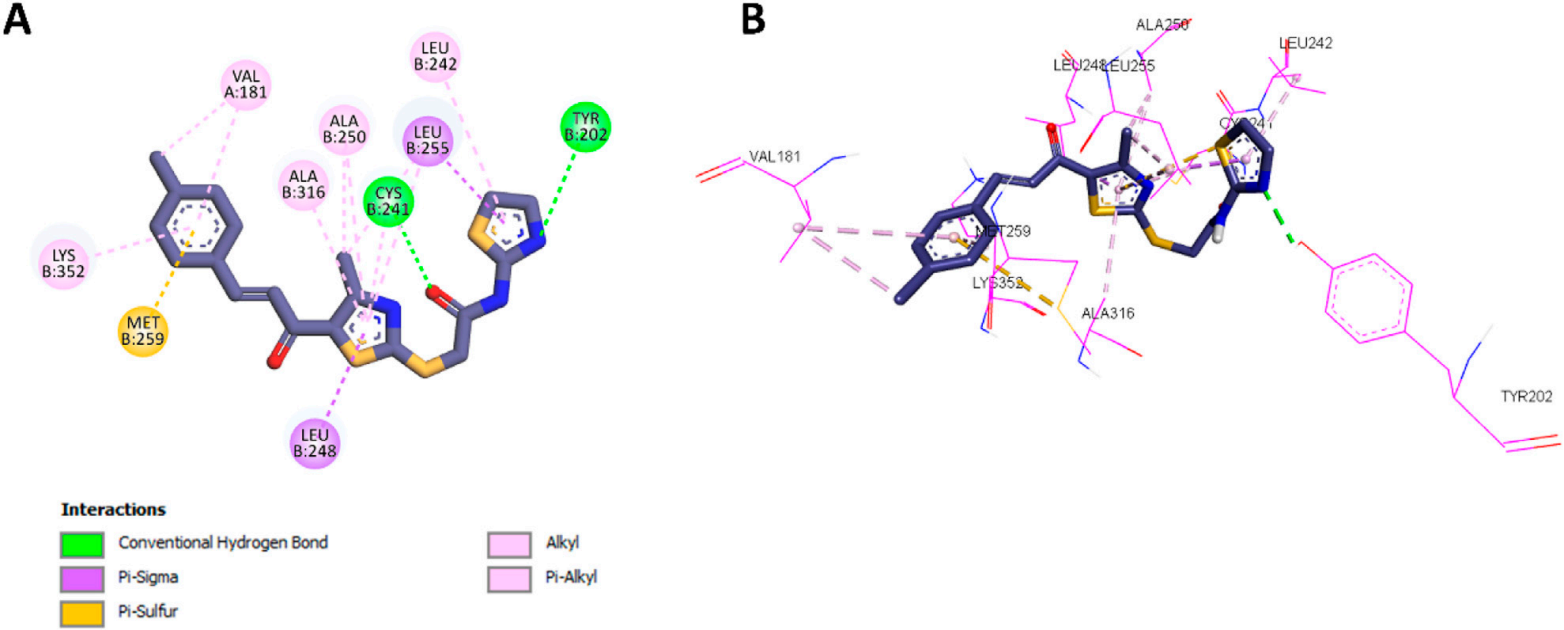
**(A)** 2D interaction diagram and **(B)** 3D binding mode of **13d** within the colchicine binding site.

Following the docking analysis of **10a**, the most potent compound in Scaffold B, **13d**, was also docked into the colchicine binding site of tubulin. Compound **13d** exhibited a binding affinity of −7.2 kcal/mol, slightly lower than **10a**. Its interaction profile revealed similar hydrophobic interactions with the same amino acids as **10a**, including Leu248, Ala316, Cys241, Ala250, Leu242, Met259, Val181, and Lys352. These conserved interactions indicate that **13d** retains a significant portion of the binding mode observed for **10a**. However, the absence of the phenyl ring attached to the thiazole moiety in **13d** limited its ability to form certain hydrophobic interactions. Specifically, **13d** could not establish π–π T-shaped interactions with Tyr202 or π-alkyl interactions with Leu252, key contributors to **10a**’s enhanced binding and potency. This structural difference likely accounts for the slightly lower potency of **13d** as a tubulin polymerization inhibitor compared to **10a**. The detailed 2D and 3D interaction profiles of **13d** within the colchicine binding site are presented in Figure 7.

The comparative docking analysis of **CA-4**, **10a**, and **13d** underscores the importance of specific structural features in enhancing binding affinity and inhibitory potency at the colchicine binding site of tubulin. These results emphasize the critical role of molecular interactions in determining potency and offer insights for the rational design of more effective tubulin polymerization inhibitors.

#### 2.3.2 ADME prediction

The SwissADME approach reveals various helpful features that distinguish compound **10a** as a potential lead chemotherapeutic agent targeting tubulin polymerization (Daina et al., 2017; Afridi et al., 2024). The molecular weight of 477.62 g/mol, although on the higher end of the permitted range for drug-like compounds, adheres to Lipinski’s rule of five without any violations. This adherence to Lipinski’s guidelines strongly indicates the compound’s drug-like properties and potential for future development. The large topological polar surface area (TPSA) of 153.73 Å^2^ indicates that it may interact with tubulin’s polar areas, making it more selective for the target. Additionally, the compound’s moderate molar refractivity (133.55) supports its compatibility with biological membranes and receptor sites.

The lipophilicity profile is notably promising, including a consensus Log P of 5.10, which balances hydrophobicity and hydrophilicity. This degree of lipophilicity is advantageous for traversing cell membranes and engaging with intracellular targets such as tubulin. Higher Log P values may be associated with solubility issues. Still, they also make it simpler for the compound to concentrate in lipid-rich cancer cell membranes, potentially increasing its efficacy and selectivity. Moreover, **10a** does not serve as a substrate for P-glycoprotein (P-gp), which is a notable advantage. P-glycoprotein-mediated efflux affects numerous anticancer agents, leading to drug resistance; **10a**’s resistance to this mechanism enhances its potential effectiveness in cancer treatment.

The compound’s pharmacokinetic characteristics enhance its attractiveness. Despite the anticipated inhibition of CYP3A4 and CYP2C9, clinical environments may alleviate these interactions with careful dose modifications and monitoring. The lack of BBB permeability is advantageous in this situation, as it diminishes the likelihood of central nervous system side effects, enhancing its suitability for systemic anticancer uses. The low skin permeability (Log Kp = −4.79 cm/s) suggests a reduced risk of unintentional cutaneous absorption, which is advantageous for handling and formulation.

**FIGURE 8:**
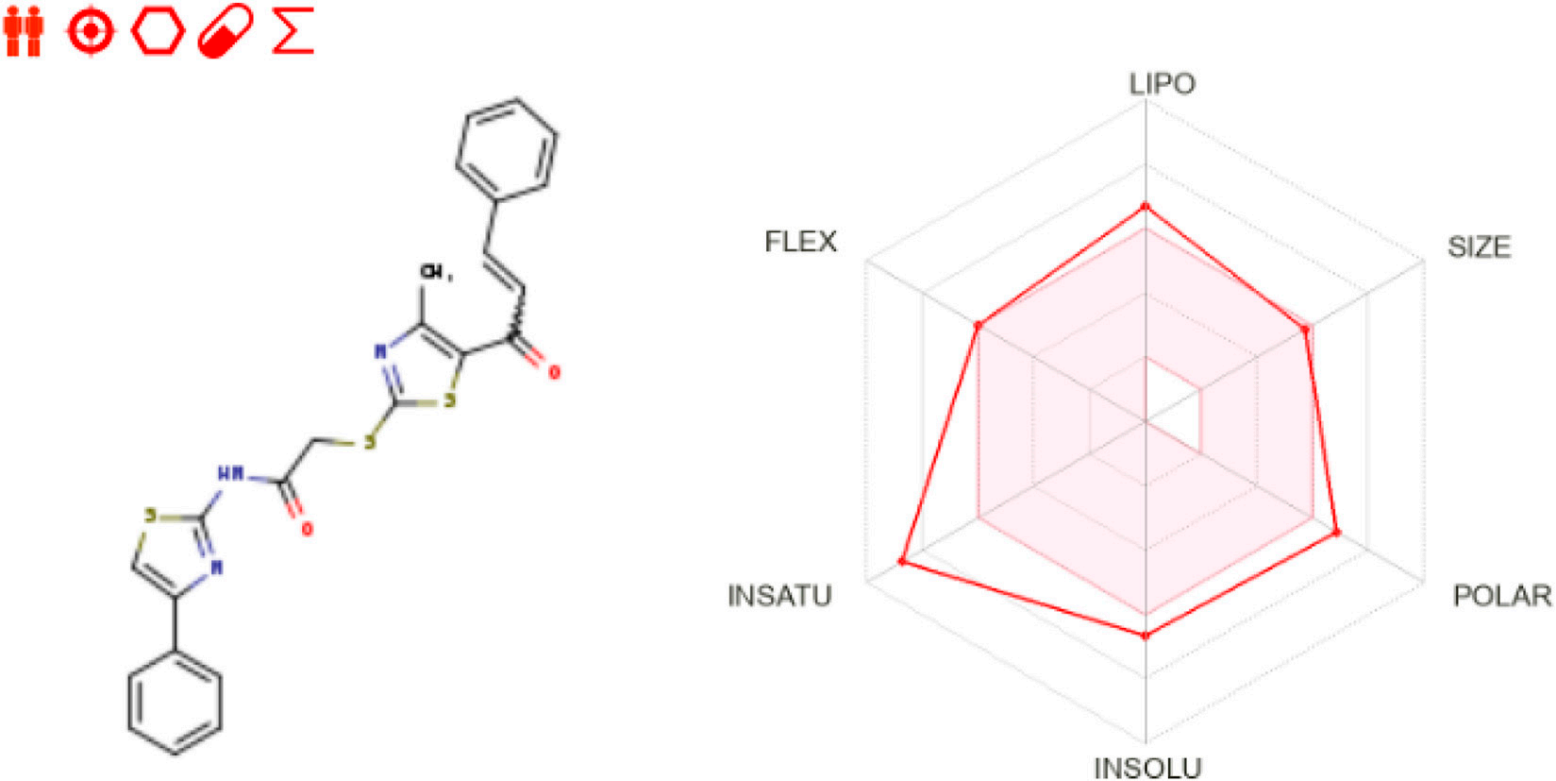
Bioavailability radar of **10a** as retrieved from SwissADME server.

Regarding drug-likeness and medicinal chemistry, **10a** is notable for its bioavailability score of 0.55, which is commendable for an orally delivered medication candidate. The bioavailability radar (Figure 8) visibly depicts the compound’s strengths. Although certain qualities, such as solubility and flexibility, are undesirable, they pose reasonable challenges in pharmaceutical development. The significant number of rotatable bonds (9) indicates that its structure is flexible, making it more likely to attach to tubulin. Also, using nanocarriers or co-crystals in the formulation process can help with its low solubility, making it easier to distribute while maintaining its natural activity.

The findings for compound **10a** indicate a robust basis for continued advancement as a tubulin polymerization inhibitor. Its drug-likeness, advantageous pharmacokinetics, and potential anticancer efficacy beyond tolerable constraints establish it as a highly useful lead compound for anticancer therapy.

## 3 Conclusion

This work describes a new series of thiazole-based compounds that inhibit tubulin polymerization, indicating their potential use in cancer therapy. All target compounds were first examined for cell viability and tubulin inhibition efficacy, and the most effective derivatives were studied as antiproliferative agents. The results show that compound **10a** is the most potent and selectively active compound at both the enzyme and cellular levels with potential apoptotic effect. Molecular docking and dynamic testing revealed that compound **10a** strongly binds to the colchicine binding site and interacts with enzymes in various ways. The findings of this investigation suggested that **10a** could be a promising candidate for additional biological testing against various cancer cell lines. This may also lead to more research into how it works, *in vivo* carcinogenic animal models, and lead optimization.

## 4 Experimental

### 4.1 Chemistry

General Details: Refer to Supplementary Appendix A.

3-Chloroacetylacetone **(2)** (Abo-Ashour et al., 2018), 1-(2-mercapto-4-methylthiazol-5-yl)ethan-1-one **(3)** and **(5a-e)** (Hashem et al., 2024), phenacyl bromides **(7a-c)**, 2-amino-4-aryl-thiazoles **(8a-c)**, and **(9a-c)** (Valiveti et al., 2015) were prepared according to reported procedures.

A mixture of chalcone derivatives **5a-e** (1 mmol), the corresponding acylated thiazole **9a-c** or **12** (1 mmol), anhydrous sodium carbonate (1.5 mmol), and sodium iodide (2 mmol) in acetone was stirred at ambient temperature for 6 h. The solvent was subsequently evaporated under reduced pressure, and the residue was thoroughly washed with a 10% sodium thiosulfate solution and distilled water. The crude product underwent recrystallization from ethanol.

#### 4.1.1 (*E*)-2-((5-cinnamoyl-4-methylthiazol-2-yl)thio)-*N*-(4-phenylthiazol-2-yl)acetamide (10a)

Yellow powder; 0.440 g, 92% yield; mp 203-207 °C; ^1^H NMR (400 MHz, DMSO-*d*_6_) δ 12.74 (s, 1H, NH), 7.91 (d, *J =* 7.2 Hz, 2H, Ar-H), 7.82–7.77 (m, 2H, Ar-H), 7.68 (d, *J =* 14.6 Hz, 2H, Ar & *CH* =CH), 7.46–7.43 (m, 5H, Ar-H), 7.38 (d, *J =* 15.6 Hz, 1H, CH= *CH*), 7.34 (t, *J =* 7.4 Hz, 1H, Ar-H), 4.43 (s, 2H, CH_2_), 2.66 (s, 3H, CH_3_); ^13^C NMR (100 MHz, DMSO-*d*_6_) δ 182.01, 168.69, 166.36, 158.38, 158.33, 149.43, 144.19, 134.67, 134.62, 132.66, 131.38, 129.50, 129.30, 129.25, 128.34, 126.15, 124.79, 108.85, 37.27, 18.78; Anal. Calcd. For C_24_H_19_N_3_O_2_S_3_: C, 60.35%; H, 4.01%; N, 8.80%. Found: C, 60.57%; H, 4.20%; N, 8.68%.

#### 4.1.2 (*E*)-2-((5-(3-(4-chlorophenyl)acryloyl)-4-methylthiazol-2-yl)thio)-*N*-(4-phenylthiazol-2-yl)acetamide (10b)

Yellow powder; 0.356 g, 70% yield; mp 245-249 °C; ^1^H NMR (400 MHz, DMSO-*d*_6_) δ 12.73 (s, 1H, NH), 7.91 (d, *J =* 7.3 Hz, 2H, Ar-H), 7.83 (d, *J =* 8.5 Hz, 2H, Ar-H), 7.66 (d, *J =* 13.4 Hz, 2H, Ar-H & *CH* =CH), 7.50 (d, *J =* 8.5 Hz, 2H, Ar-H), 7.45 (t, *J =* 7.7 Hz, 2H, Ar-H), 7.39 (d, *J =* 15.6 Hz, 1H, CH= *CH*), 7.34 (t, *J =* 7.4 Hz, 1H, Ar-H), 4.44 (s, 2H, CH_2_), 2.66 (s, 3H, CH_3_); ^13^C NMR (100 MHz, DMSO-*d*_6_) δ 181.87, 168.76, 166.28, 158.49, 158.17, 149.45, 142.71, 135.85, 134.63, 133.59, 132.56, 130.99, 129.52, 129.20, 128.36, 126.15, 125.48, 108.91, 37.19, 18.78; Anal. Calcd. For C_24_H_18_ClN_3_O_2_S_3_: C, 56.30%; H, 3.54%; N, 8.21%. Found: C, 56.16%; H, 3.62%; N, 8.11%.

#### 4.1.3 (*E*)-2-((5-(3-(4-fluorophenyl)acryloyl)-4-methylthiazol-2-yl)thio)-*N*-(4-phenylthiazol-2-yl)acetamide (10c)

Yellow powder; 0.427 g, 86% yield; mp 211-213 °C; ^1^H NMR (400 MHz, DMSO-*d*_6_) δ 12.74 (s, 1H, NH), 7.94–7.86 (m, 4H, Ar-H), 7.71–7.66 (m, 2H, Ar-H & *CH* =CH), 7.44 (t, *J =* 7.7 Hz, 2H, Ar-H & CH= *CH*), 7.36 (s, 1H, Ar-H), 7.33 (d, *J =* 7.2 Hz, 1H, Ar-H), 7.29 (t, *J =* 8.8 Hz, 2H, Ar-H), 4.43 (s, 2H, CH_2_), 2.66 (s, 3H, CH_3_); ^13^C NMR (100 MHz, DMSO-*d*_6_) δ 181.94, 168.64, 166.31, 158.33, 149.44, 143.00, 134.65, 132.63, 131.77 (d, *J* = 8.67), 131.70, 131.30 (d, *J* = 3.07), 129.25, 128.35, 126.15, 124.68, 116.62, 116.44 (d, *J* = 21.83), 108.89, 37.21, 18.77; Anal. Calcd. For C_24_H_18_FN_3_O_2_S_3_: C, 58.16%; H, 3.66%; N, 8.48%. Found: C, 58.37%; H, 3.88%; N, 8.34%.

#### 4.1.4 (*E*)-2-((4-methyl-5-(3-(*p*-tolyl)acryloyl)thiazol-2-yl)thio)-*N*-(4-phenylthiazol-2-yl)acetamide (10d)

Yellow powder; 0.441 g, 90% yield; mp 227-230 °C; ^1^H NMR (400 MHz, DMSO-*d*_6_) δ 12.74 (s, 1H, NH), 7.92 (dd, *J =* 8.3, 1.1 Hz, 2H, Ar-H), 7.68 (d, *J =* 3.1 Hz, 2H), 7.65 (d, *J =* 17.0 Hz, 2H), 7.45 (t, *J =* 7.7 Hz, 2H), 7.35 (d, *J =* 8.5 Hz, 1H), 7.30 (d, *J =* 15.5 Hz, 1H), 7.25 (d, *J =* 8.1 Hz, 2H), 4.43 (s, 2H), 2.65 (s, 3H), 2.33 (s, 3H); ^13^C NMR (100 MHz, DMSO-*d*_6_) δ 181.92, 168.41, 166.31, 158.19, 149.45, 144.30, 141.56, 134.64, 132.67, 131.87, 130.14, 130.11, 129.33, 129.25, 128.35, 126.16, 123.71, 108.90, 37.19, 21.58, 18.74; Anal. Calcd. For C_25_H_21_N_3_O_2_S_3_: C, 61.08%; H, 4.31%; N, 8.55%. Found: C, 60.87%; H, 4.22%; N, 8.69%.

#### 4.1.5 (*E*)-2-((5-(3-(4-methoxyphenyl)acryloyl)-4-methylthiazol-2-yl)thio)-*N*-(4-phenylthiazol-2-yl)acetamide (10e)

Yellow powder; 0.334 g, 66% yield; mp 220-223 °C; ^1^H NMR (400 MHz, DMSO-*d*_6_) δ 12.73 (s, 1H, NH), 7.91 (d, *J =* 7.3 Hz, 2H), 7.76 (d, *J =* 8.7 Hz, 2H), 7.66 (d, *J =* 17.2 Hz, 2H), 7.44 (t, *J =* 7.7 Hz, 2H), 7.34 (t, *J =* 7.4 Hz, 1H), 7.23 (d, *J =* 15.4 Hz, 1H), 7.00 (d, *J =* 8.7 Hz, 2H), 4.42 (s, 2H), 3.81 (s, 3H), 2.65 (s, 3H); ^13^C NMR (100 MHz, DMSO-*d*_6_) δ 181.86, 168.14, 166.35, 162.09, 158.29, 157.90, 149.43, 144.31, 134.66, 132.81, 131.29, 129.25, 128.35, 127.21, 126.15, 122.21, 114.99, 108.87, 55.89, 37.22, 18.72; Anal. Calcd. For C_25_H_21_N_3_O_3_S_3_: C, 59.15%; H, 4.17%; N, 8.28%. Found: C, 58.94%; H, 4.11%; N, 8.46%.

#### 4.1.6 (*E*)-N-(4-(4-chlorophenyl)thiazol-2-yl)-2-((5-cinnamoyl-4-methylthiazol-2-yl)thio) acetamide (10f)

Yellow powder; 0.360 g, 70% yield; mp 251-253 °C; ^1^H NMR (400 MHz, DMSO-*d*_6_) δ 12.74 (s, 1H, NH), 7.93 (d, *J =* 8.5 Hz, 2H), 7.82–7.78 (m, 2H), 7.74 (s, 1H), 7.68 (d, *J =* 15.5 Hz, 1H), 7.51 (d, *J =* 8.6 Hz, 2H), 7.47–7.43 (m, 3H), 7.38 (d, *J =* 15.5 Hz, 1H), 4.44 (s, 2H), 2.66 (s, 3H); ^13^C NMR (100 MHz, DMSO-*d*_6_) δ 182.00, 168.57, 166.38, 158.38, 158.30, 148.21, 144.19, 134.61, 133.50, 132.81, 132.68, 131.38, 129.49, 129.29, 129.28, 127.87, 124.76, 109.65, 37.19, 18.78; Anal. Calcd. For C_24_H_18_ClN_3_O_2_S_3_: C, 56.30%; H, 3.54%; N, 8.21%. Found: C, 56.25%; H, 3.72%; N, 8.12%.

#### 4.1.7 (*E*)-2-((5-(3-(4-chlorophenyl)acryloyl)-4-methylthiazol-2-yl)thio)-*N*-(4-(4-chlorophenyl) thiazol-2-yl)acetamide (10g)

Yellow powder; 0.467 g, 85.45% yield; mp 260-263 °C; ^1^H NMR (400 MHz, DMSO-*d*_6_) δ 12.73 (s, 1H, NH), 7.92 (d, *J =* 8.3 Hz, 2H), 7.84 (d, *J =* 8.3 Hz, 2H), 7.74 (s, 1H), 7.67 (d, *J =* 15.5 Hz, 1H), 7.51 (d, *J =* 8.3 Hz, 4H), 7.39 (d, *J =* 15.6 Hz, 1H), 4.43 (s, 2H), 2.65 (s, 3H); ^13^C NMR (100 MHz, DMSO-*d*_6_) δ 181.90, 168.77, 166.39, 158.49, 148.20, 143.42, 142.72, 135.85, 133.60, 133.52, 132.80, 132.55, 131.01, 129.52, 129.28, 127.87, 125.51, 109.63, 37.23, 18.78; Anal. Calcd. For C_24_H_17_Cl_2_N_3_O_2_S_3_: C, 52.75%; H, 3.14%; N, 7.69%. Found: C, 52.86%; H, 3.20%; N, 7.60%.

#### 4.1.8 (*E*)-N-(4-(4-chlorophenyl)thiazol-2-yl)-2-((5-(3-(4-fluorophenyl)acryloyl)-4-methyl thiazol-2-yl)thio)acetamide (10h)

Yellow powder; 0.482 g, 90.93% yield; mp 217-219 °C; ^1^H NMR (400 MHz, DMSO-*d*_6_) δ 12.74 (s, 1H, NH), 7.93 (d, *J =* 8.6 Hz, 2H), 7.89 (dd, *J =* 8.7, 5.6 Hz, 2H), 7.74 (s, 1H), 7.68 (d, *J =* 15.6 Hz, 1H), 7.51 (d, *J =* 8.6 Hz, 2H), 7.34 (d, *J =* 15.6 Hz, 1H), 7.29 (t, *J =* 8.8 Hz, 2H), 4.43 (s, 2H), 2.65 (s, 3H); ^13^C NMR (100 MHz, DMSO-*d*_6_) δ 181.94, 168.61, 166.40, 165.01, 163.03, 158.32, 148.20, 143.00, 133.52, 132.80, 132.63, 131.70 (d, *J* = 8.68), 131.32 (d, *J* = 2.94), 129.28, 127.87, 124.66, 116.44 (d, *J* = 21.86), 109.63, 37.22, 18.77; Anal. Calcd. For C_24_H_17_ClFN_3_O_2_S_3_: C, 54.38%; H, 3.23%; N, 7.93%. Found: C, 54.31%; H, 3.11%; N, 7.98%.

#### 4.1.9 (*E*)-N-(4-(4-chlorophenyl)thiazol-2-yl)-2-((4-methyl-5-(3-(*p*-tolyl)acryloyl)thiazol-2-yl)thio)acetamide (10i)

Yellow powder; 0.445 g, 84.59% yield; mp 240-241 °C; ^1^H NMR (400 MHz, DMSO-*d*_6_) δ 12.74 (s, 1H, NH), 7.93 (d, *J =* 8.6 Hz, 2H), 7.71 (d, *J =* 18.6 Hz, 2H), 7.68–7.62 (m, 2H), 7.50 (d, *J =* 8.6 Hz, 2H), 7.31 (d, *J =* 15.5 Hz, 1H), 7.25 (d, *J =* 8.0 Hz, 2H), 4.42 (s, 2H), 2.65 (s, 3H), 2.34 (s, 3H); ^13^C NMR (100 MHz, DMSO-*d*_6_) δ 181.93, 168.51, 166.49, 158.21, 148.17, 144.30, 141.56, 133.58, 132.83, 132.76, 132.62, 131.88, 130.12, 129.34, 129.27, 127.87, 123.73, 109.55, 37.35, 21.58, 18.74; Anal. Calcd. For C_25_H_20_ClN_3_O_2_S_3_: C, 57.08%; H, 3.83%; N, 7.99%. Found: C, 57.30%; H, 3.67%; N, 7.88%.

#### 4.1.10 (*E*)-*N*-(4-(4-chlorophenyl)thiazol-2-yl)-2-((5-(3-(4-methoxyphenyl)acryloyl)-4-methylthiazol-2-yl)thio)acetamide (10j)

Yellow powder; 0.361 g, 67% yield; mp 223-226 °C; ^1^H NMR (400 MHz, DMSO-*d*_6_) δ 12.74 (s, 1H, NH), 7.93 (d, *J =* 8.6 Hz, 2H), 7.76 (d, *J =* 8.8 Hz, 2H), 7.72 (s, 1H), 7.65 (d, *J =* 15.4 Hz, 1H), 7.50 (d, *J =* 8.6 Hz, 2H), 7.23 (d, *J =* 15.4 Hz, 1H), 7.00 (d, *J =* 8.8 Hz, 2H), 4.41 (s, 2H), 3.81 (s, 3H), 2.65 (s, 3H); ^13^C NMR (100 MHz, DMSO-*d*_6_) δ 181.85, 168.23, 166.53, 162.09, 157.91, 148.15, 144.30, 133.59, 132.77, 132.74, 131.33, 131.30, 129.27, 127.86, 127.21, 122.22, 114.99, 109.53, 55.89, 37.37, 18.72; Anal. Calcd. For C_25_H_20_ClN_3_O_3_S_3_: C, 55.39%; H, 3.72%; N, 7.75%. Found: C, 55.59%; H, 3.8%; N, 7.53%.

#### 4.1.11 (*E*)-2-((5-cinnamoyl-4-methylthiazol-2-yl)thio)-*N*-(4-(p-tolyl)thiazol-2-yl)acetamide (10k)

Yellow powder; 0.364 g, 74% yield; mp 255-257 °C; ^1^H NMR (400 MHz, DMSO-*d*_6_) δ 12.70 (s, 1H, NH), 7.80 (d, *J =* 7.8 Hz, 4H), 7.68 (d, *J =* 15.5 Hz, 1H), 7.59 (s, 1H), 7.47–7.44 (m, 3H), 7.38 (d, *J =* 15.6 Hz, 1H), 7.25 (d, *J =* 8.0 Hz, 2H), 4.43 (s, 2H), 2.66 (s, 3H), 2.33 (s, 3H); ^13^C NMR (100 MHz, DMSO-*d*_6_) δ 182.01, 168.59, 166.21, 158.31, 157.97, 149.55, 144.19, 137.69, 134.60, 132.69, 131.97, 131.38, 129.81, 129.50, 129.29, 126.10, 124.76, 108.04, 37.14, 21.29, 18.78; Anal. Calcd. For C_25_H_21_N_3_O_2_S_3_: C, 61.08%; H, 4.31%; N, 8.55%. Found: C, 60.96%; H, 4.18%; N, 8.72%.

#### 4.1.12 (*E*)-2-((5-(3-(4-chlorophenyl)acryloyl)-4-methylthiazol-2-yl)thio)-*N*-(4-(*p*-tolyl)thiazol-2-yl)acetamide (10l)

Pale yellow crystal; 0.358 g, 68% yield; mp 229-232 °C; ^1^H NMR (400 MHz, DMSO-*d*_6_) δ 12.70 (s, 1H, NH), 7.83 (d, *J =* 8.6 Hz, 2H), 7.80 (d, *J =* 8.1 Hz, 2H), 7.67 (d, *J =* 15.6 Hz, 1H), 7.60 (s, 1H), 7.50 (d, *J =* 8.5 Hz, 2H), 7.39 (d, *J =* 15.6 Hz, 1H), 7.25 (d, *J =* 8.0 Hz, 2H), 4.42 (s, 2H), 2.66 (s, 3H), 2.33 (s, 3H); ^13^C NMR (100 MHz, DMSO-*d*_6_) δ 181.90, 170.25, 166.21, 165.49, 158.50, 151.75, 142.73, 137.70, 137.45, 135.85, 133.60, 132.56, 131.01, 129.82, 129.53, 126.10, 125.51, 108.04, 37.42, 21.29, 18.78; Anal. Calcd. For C_25_H_20_ClN_3_O_2_S_3_: C, 57.08%; H, 3.83%; N, 7.99%. Found: C, 57.27%; H, 4.05%; N, 7.91%.

#### 4.1.13 (*E*)-2-((5-(3-(4-fluorophenyl)acryloyl)-4-methylthiazol-2-yl)thio)-*N*-(4-(*p*-tolyl)thiazol-2-yl)acetamide (10m)

Yellow powder; 0.399 g, 78% yield; mp 237-238 °C; ^1^H NMR (400 MHz, DMSO-*d*_6_) δ 12.70 (s, 1H, NH), 7.89 (dd, *J =* 8.6, 5.7 Hz, 2H), 7.80 (d, *J =* 8.1 Hz, 2H), 7.68 (d, *J =* 15.5 Hz, 1H), 7.59 (s, 1H), 7.34 (d, *J =* 15.5 Hz, 1H), 7.29 (t, *J =* 8.8 Hz, 2H), 7.25 (d, *J =* 8.2 Hz, 2H), 4.42 (s, 2H), 2.66 (s, 3H), 2.33 (s, 3H); ^13^C NMR (100 MHz, DMSO-*d*_6_) δ 181.94, 168.64, 166.24, 165.01, 163.03, 158.34, 149.52, 143.00, 137.67, 132.62, 132.00, 131.76 (d, *J* = 8.71), 131.30 (d, *J* = 3.11), 129.80, 126.10, 124.66, 116.44 (d, *J* = 21.83), 108.00, 37.21, 21.28, 18.77; Anal. Calcd. For C_25_H_20_FN_3_O_2_S_3_: C, 58.92%; H, 3.96%; N, 8.25%. Found: C, 7.91%; H, 3.79%; N, 8.06%.

#### 4.1.14 (*E*)-2-((4-methyl-5-(3-(*p*-tolyl)acryloyl)thiazol-2-yl)thio)-*N*-(4-(*p*-tolyl)thiazol-2-yl)acetamide (10n)

Yellow powder; 0.420 g, 83% yield; mp 248-250 °C; ^1^H NMR (400 MHz, DMSO-*d*_6_) δ 12.68 (s, 1H, NH), 7.80 (d, *J =* 8.1 Hz, 2H), 7.68 (d, *J =* 8.1 Hz, 2H), 7.65 (d, *J =* 15.6 Hz, 1H), 7.59 (s, 1H), 7.31 (d, *J =* 15.5 Hz, 1H), 7.25 (d, *J =* 7.9 Hz, 4H), 4.42 (s, 2H), 2.65 (s, 3H), 2.33 (s, 6H); ^13^C NMR (100 MHz, DMSO-*d*_6_) δ 181.95, 168.45, 166.25, 158.20, 144.32, 141.57, 137.67, 132.66, 132.01, 131.88, 130.13, 129.81, 129.34, 126.36, 126.30, 126.11, 123.73, 108.02, 37.19, 21.58, 21.29, 18.74; Anal. Calcd. For C_26_H_23_N_3_O_2_S_3_: C, 61.76%; H, 4.58%; N, 8.31%. Found: C, 61.61%; H, 4.63%; N, 8.10%.

#### 4.1.15 (*E*)-2-((5-(3-(4-methoxyphenyl)acryloyl)-4-methylthiazol-2-yl)thio)-*N*-(4-(*p*-tolyl) thiazol-2-yl)acetamide (10o)

Yellow powder; 0.378 g, 72% yield; mp 240-242 °C; ^1^H NMR (400 MHz, DMSO-*d*_6_) δ 12.70 (s, 1H, NH), 7.80 (d, *J =* 8.1 Hz, 2H), 7.76 (d, *J =* 8.8 Hz, 2H), 7.65 (d, *J =* 15.4 Hz, 1H), 7.59 (s, 1H), 7.26 (s, 1H), 7.22 (d, *J =* 15.6 Hz, 2H), 7.00 (d, *J =* 8.8 Hz, 2H), 4.42 (s, 2H), 3.81 (s, 3H), 2.65 (s, 3H), 2.33 (s, 3H); ^13^C NMR (100 MHz, DMSO-*d*_6_) δ 181.86, 168.11, 166.25, 162.09, 158.04, 157.90, 149.54, 144.31, 137.68, 132.82, 131.99, 131.29, 129.81, 127.20, 126.10, 122.21, 114.99, 108.03, 55.88, 37.16, 21.29, 18.71; Anal. Calcd. For C_26_H_23_N_3_O_3_S_3_: C, 59.86%; H, 4.44%; N, 8.06%. Found: C, 59.64%; H, 4.50%; N, 8.16%.

#### 4.1.16 (*E*)-2-((5-cinnamoyl-4-methylthiazol-2-yl)thio)-*N*-(thiazol-2-yl)acetamide (13a)

Yellow powder; 0.272 g, 67% yield; mp 215-219 °C; ^1^H NMR (400 MHz, DMSO-*d*_6_) δ 12.55 (s, 1H, NH), 7.80 (dd, *J =* 6.7, 2.9 Hz, 2H), 7.68 (d, *J =* 15.5 Hz, 1H), 7.51 (d, *J =* 3.6 Hz, 1H), 7.46 (dd, *J =* 5.0, 1.7 Hz, 3H), 7.37 (d, *J =* 15.5 Hz, 1H), 7.26 (d, *J =* 3.6 Hz, 1H), 4.41 (s, 2H), 2.66 (s, 3H); ^13^C NMR (100 MHz, DMSO-*d*_6_) δ 181.99, 168.62, 165.97, 158.30, 158.26, 144.19, 138.26, 134.61, 132.65, 131.39, 129.50, 129.30, 124.77, 114.38, 37.17, 18.76; Anal. Calcd. For C_18_H_15_N_3_O_2_S_3_: C, 53.85%; H, 3.77%; N, 10.47%. Found: C, 53.68%; H, 3.56%; N, 10.67%.

#### 4.1.17 (*E*)-2-((5-(3-(4-chlorophenyl)acryloyl)-4-methylthiazol-2-yl)thio)-*N*-(thiazol-2-yl)acetamide (13b)

Yellow powder; 0.335 g, 77% yield; mp 221-224 °C; ^1^H NMR (400 MHz, DMSO-*d*_6_) δ 12.55 (s, 1H, NH), 7.85 (d, *J =* 8.5 Hz, 2H), 7.67 (d, *J =* 15.6 Hz, 1H), 7.52 (d, *J =* 8.5 Hz, 2H), 7.51 (d, *J =* 3.6 Hz, 1H), 7.39 (d, *J =* 15.6 Hz, 1H), 7.26 (d, *J =* 3.6 Hz, 1H), 4.41 (s, 2H), 2.65 (s, 3H); ^13^C NMR (100 MHz, DMSO-*d*_6_) δ 181.89, 168.77, 165.95, 158.46, 158.24, 142.72, 138.27, 135.85, 133.60, 132.57, 131.02, 129.53, 125.48, 114.39, 37.16, 18.78; Anal. Calcd. For C_18_H_14_ClN_3_O_2_S_3_: C, 49.59%; H, 3.24%; N, 9.64%. Found: C, 49.77%; H, 3.09%; N, 9.73%.

#### 4.1.18 (*E*)-2-((5-(3-(4-fluorophenyl)acryloyl)-4-methylthiazol-2-yl)thio)-*N*-(thiazol-2-yl)acetamide (13c)

Yellow powder; 0.344 g, 82% yield; mp 218-222 °C; ^1^H NMR (400 MHz, DMSO-*d*_6_) δ 12.54 (s, 1H, NH), 7.89 (dd, *J =* 8.7, 5.6 Hz, 2H), 7.68 (d, *J =* 15.5 Hz, 1H), 7.51 (d, *J =* 3.6 Hz, 1H), 7.36–7.28 (m, 3H), 7.26 (d, *J =* 3.6 Hz, 1H), 4.40 (s, 2H), 2.65 (s, 3H); ^13^C NMR (100 MHz, DMSO-*d*_6_) δ 181.94, 168.64, 165.97, 165.02, 163.04, 158.31, 143.00, 138.26, 132.62, 131.71 (d, *J* = 8.72), 131.30 (d, *J* = 3.13), 124.67, 116.63 (d, *J* = 21.80), 114.37, 37.18, 18.76; Anal. Calcd. For C_18_H_14_FN_3_O_2_S_3_: C, 51.54%; H, 3.36%; N, 10.02%. Found: C, 51.37%; H, 3.30%; N, 10.18%.

#### 4.1.19 (*E*)-2-((4-methyl-5-(3-(*p*-tolyl)acryloyl)thiazol-2-yl)thio)-*N*-(thiazol-2-yl)acetamide (13d)

Yellow powder; 0.356 g, 86% yield; mp 212-215 °C; ^1^H NMR (400 MHz, DMSO-*d*_6_) δ 12.54 (s, 1H, NH), 7.69 (d, *J =* 8.1 Hz, 2H), 7.65 (d, *J =* 15.5 Hz, 1H), 7.51 (d, *J =* 3.5 Hz, 1H), 7.31 (d, *J =* 15.5 Hz, 1H), 7.28 (s, 1H), 7.26 (d, *J =* 3.6 Hz, 2H), 4.40 (s, 2H), 2.65 (s, 3H), 2.35 (s, 3H); ^13^C NMR (100 MHz, DMSO-*d*_6_) δ 181.95, 168.42, 165.98, 158.27, 158.14, 144.32, 141.59, 138.26, 132.70, 131.89, 130.13, 129.36, 123.70, 114.37, 37.17, 21.59, 18.73; Anal. Calcd. For C_19_H_17_N_3_O_2_S_3_: C, 54.92%; H, 4.12%; N, 10.11%. Found: C, 55.06%; H, 4.01%; N, 10.04%.

#### 4.1.20 (*E*)-2-((5-(3-(4-methoxyphenyl)acryloyl)-4-methylthiazol-2-yl)thio)-*N*-(thiazol-2-yl)acetamide (13e)

Yellow powder; 0.332 g, 77% yield; mp 225-227 °C; ^1^H NMR (400 MHz, DMSO-*d*_6_) δ 12.54 (s, 1H, NH), 7.77 (d, *J =* 8.6 Hz, 2H), 7.66 (d, *J =* 15.4 Hz, 1H), 7.50 (d, *J =* 3.5 Hz, 1H), 7.26 (d, *J =* 3.6 Hz, 1H), 7.23 (d, *J =* 15.4 Hz, 1H), 7.02 (d, *J =* 8.7 Hz, 2H), 4.40 (s, 2H), 3.82 (s, 3H), 2.64 (s, 3H); ^13^C NMR (100 MHz, DMSO-*d*_6_) δ 181.86, 168.17, 166.03, 162.10, 158.40, 157.87, 144.31, 138.25, 132.81, 131.31, 127.21, 122.19, 115.01, 114.33, 55.90, 37.22, 18.70; Anal. Calcd. For C_19_H_17_N_3_O_3_S_3_: C, 52.88%; H, 3.97%; N, 9.74%. Found: C, 52.75%; H, 3.89%; N, 9.96%.

### 4.2 Biology

#### 4.2.1 Cell viability assay

The effects of compounds **10a-o** and **13a-e** on cell viability were assessed using the human mammary gland epithelial normal cell line (MCF-10A). The MTT assay assessed the cell viability of compounds **10a-o** and **13a-e** following a four-day incubation with MCF-10A cells (El-Sherief et al., 2019; Ramadan et al., 2020). Check Supplementary Appendix A for additional information.

#### 4.2.2 Tubulin polymerization inhibition assay

Compounds **10a-o** and **13a-e** effects on tubulin polymerization were investigated using the Tubulin Polymerization Assay Kit (Cytoskeleton Inc., Denver, CO, USA) according to the supplier’s instructions (Abdelbaset et al., 2021; Abdelbaset et al., 2018). Details are presented in Supplementary Appendix A.

#### 4.2.3 Antiproliferative assay

The antiproliferative efficacy of novel compounds **10a**, **10d**, **10f**, **10h**, **10m**, **10o** (Scaffold A) and **13b-d** (Scaffold B) against four human cancer cell lines (colon - HCT-116, prostatic - PC-3, breast -MCF-7, and pancreatic-MDAMB-231) was evaluated utilizing the MTT test (Mahmoud et al., 2022; Hisham et al., 2022; Al-Wahaibi et al., 2023). Doxorubicin and Sorafenib were used as controls in this study.

#### 4.2.4 Apoptotic markers assays

Compounds **10a**, **10o**, and **13d** were assessed for their ability to activate caspase-3, caspase-9, and Bax, as well as to downregulate the anti-apoptotic protein Bcl2 in the MDAMB-231 pancreatic cancer cell line (Youssif et al., 2019). Supplementary Appendix A provides more information.

## Data Availability

The original contributions presented in the study are included in the article/Supplementary Material, further inquiries can be directed to the corresponding authors.
