# Supplementary material for: Design, synthesis, in silico studies, and apoptotic antiproliferative activity of novel thiazole-2-acetamide derivatives as tubulin polymerization inhibitors
Source: Front Chem. 2025 Apr 16;13:1565699. doi: 10.3389/fchem.2025.1565699 (PMC12040969; doi:10.3389/fchem.2025.1565699)

Design, synthesis, *in silico* studies, and apoptotic antiproliferative activity of novel thiazole-2-acetamide derivatives as tubulin polymerization inhibitors

Lamya H. Al-Wahaibi[1], Ali M. Elshamsy[2], Taha F.S. Ali[3], Bahaa G. M. Youssif*[4], S. Bräse[5]*, Mohamed Abdel-Aziz*[3], Nawal A. El-Koussi[2,6]

[1]Department of Chemistry, College of Sciences, Princess Nourah bint Abdulrahman University, Riyadh 11671, Saudi Arabia; [2]Pharmceutical Chemistry Department, Faculty of Pharmacy, Deraya University, Minia, Egypt; [3]Medicinal Chemistry Department, Faculty of Pharmacy, Minia University, Minia 61519, Egypt; [4]Department of Pharmaceutical Organic Chemistry, Faculty of Pharmacy, Assiut University, Assiut-71526, Egypt; [5]Institute of Biological and Chemical Systems, IBCS-FMS, Karlsruhe Institute of Technology, 76131 Karlsruhe, Germany [6]Department of Pharmaceutical Medicinal Chemistry, Faculty of Pharmacy, Assiut University, Assiut, Egypt.

*To whom correspondence should be addressed:

Mohamed Abdel-Aziz, Ph.D. Department of Medicinal Chemistry, Faculty of Pharmacy, Minia University, 61519-Minia, Egypt.

Tel.: +2101003311327; E-mail address: abulnil@hotmail.com

Bahaa G. M. Youssif, Ph.D. Pharmaceutical Organic Chemistry Department, Faculty of Pharmacy, Assiut University, Assiut 71526, Egypt.

Tel.: +201098294419; E-mail address: bgyoussif2@gmail.com

S. Bräse

Institute of Biological and Chemical Systems, IBCS-FMS, Karlsruhe Institute of Technology, 76131 Karlsruhe, Germany. E-mail: braese@kit.edu

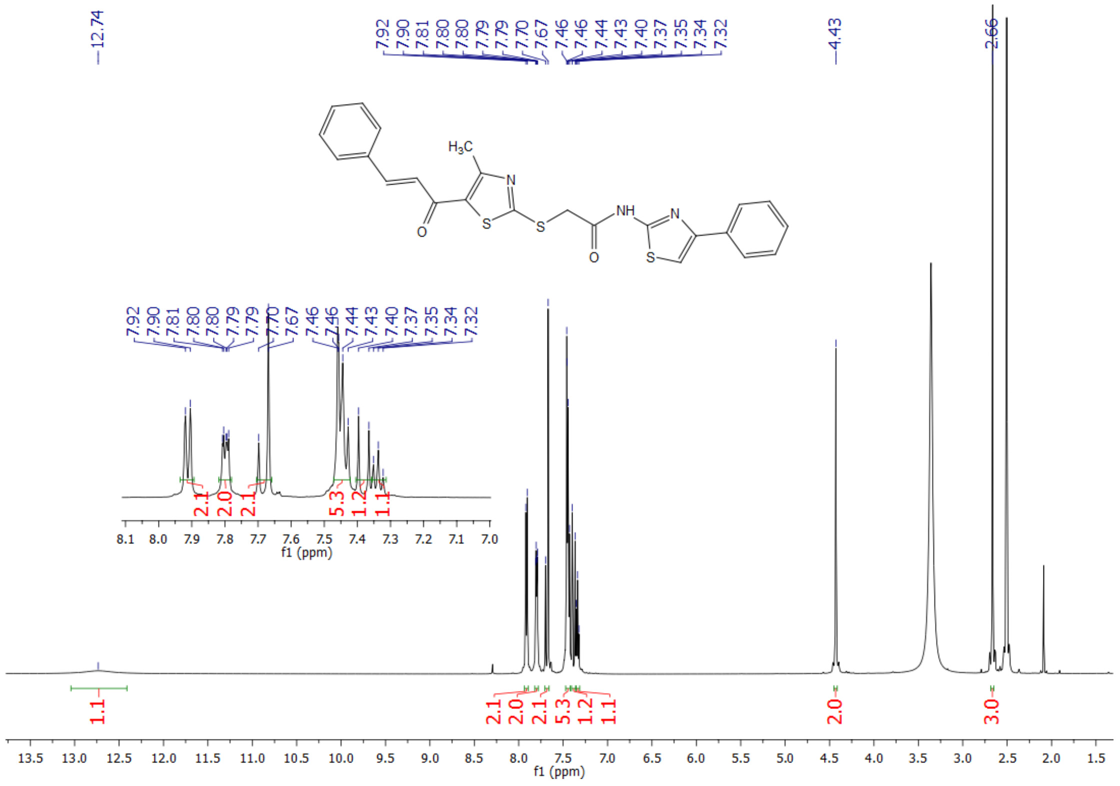

Figure S1. ^{1}H NMR (400 MHz, DMSO-d_6) spectrum of **10a**

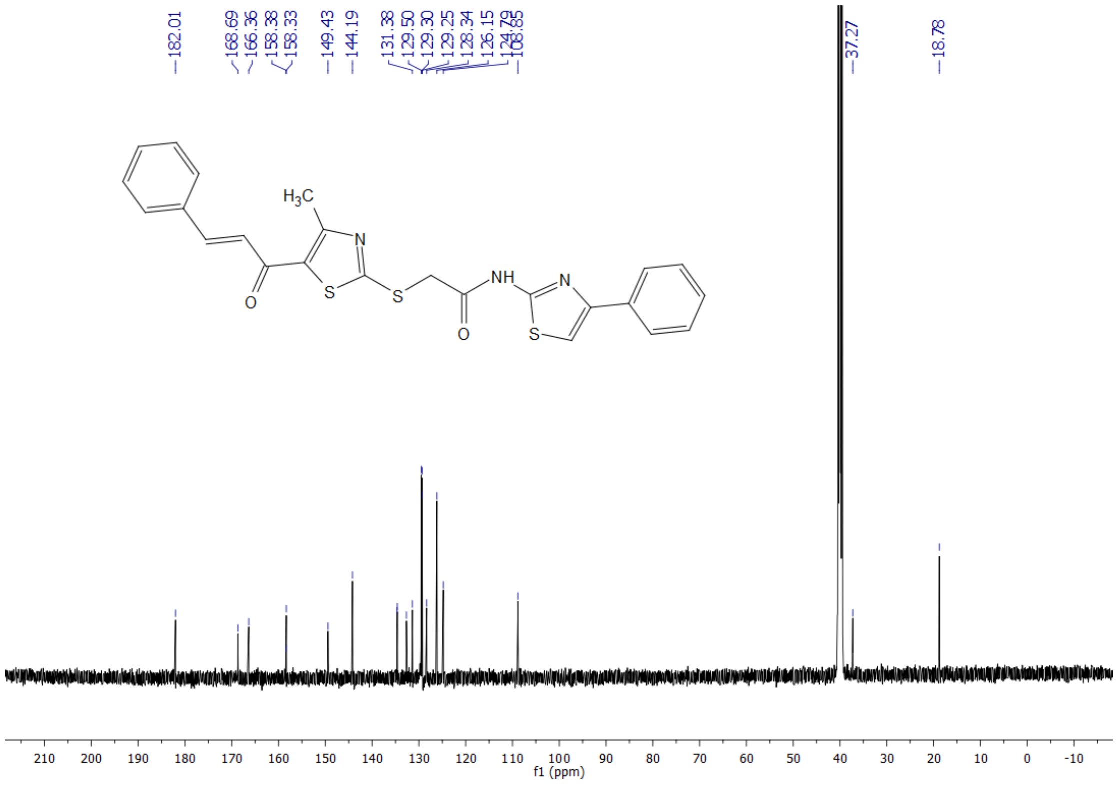

Figure S2. ^{13}C NMR (100 MHz, DMSO-d_6) spectrum of **10a**

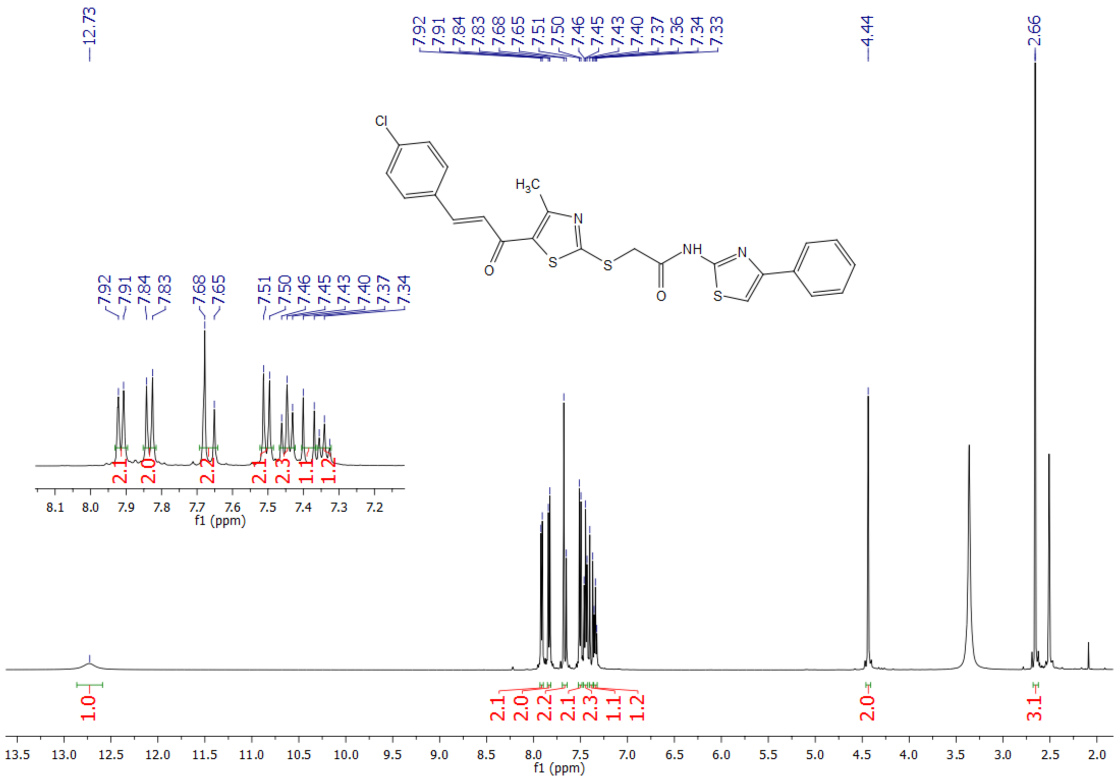

Figure S3. ^{1}H NMR (400 MHz, DMSO-d_6) spectrum of **10b**

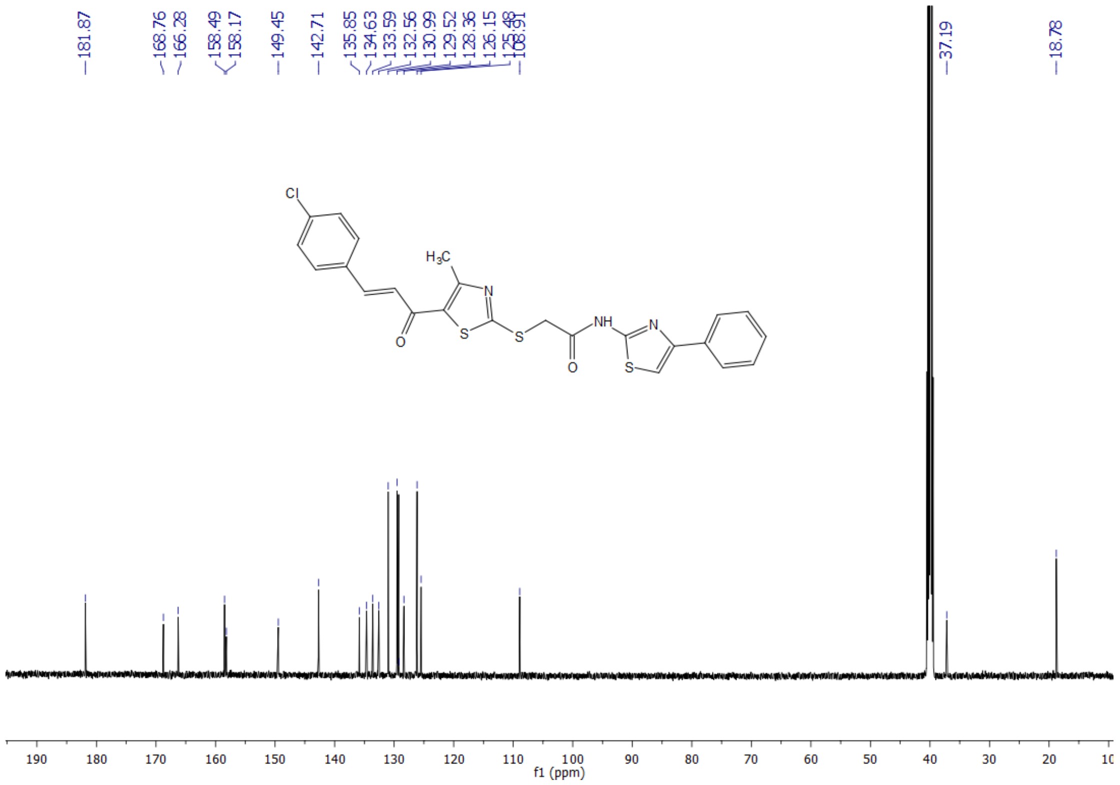

Figure S4. ^{13}C NMR (100 MHz, DMSO-d_6) spectrum of **10b**

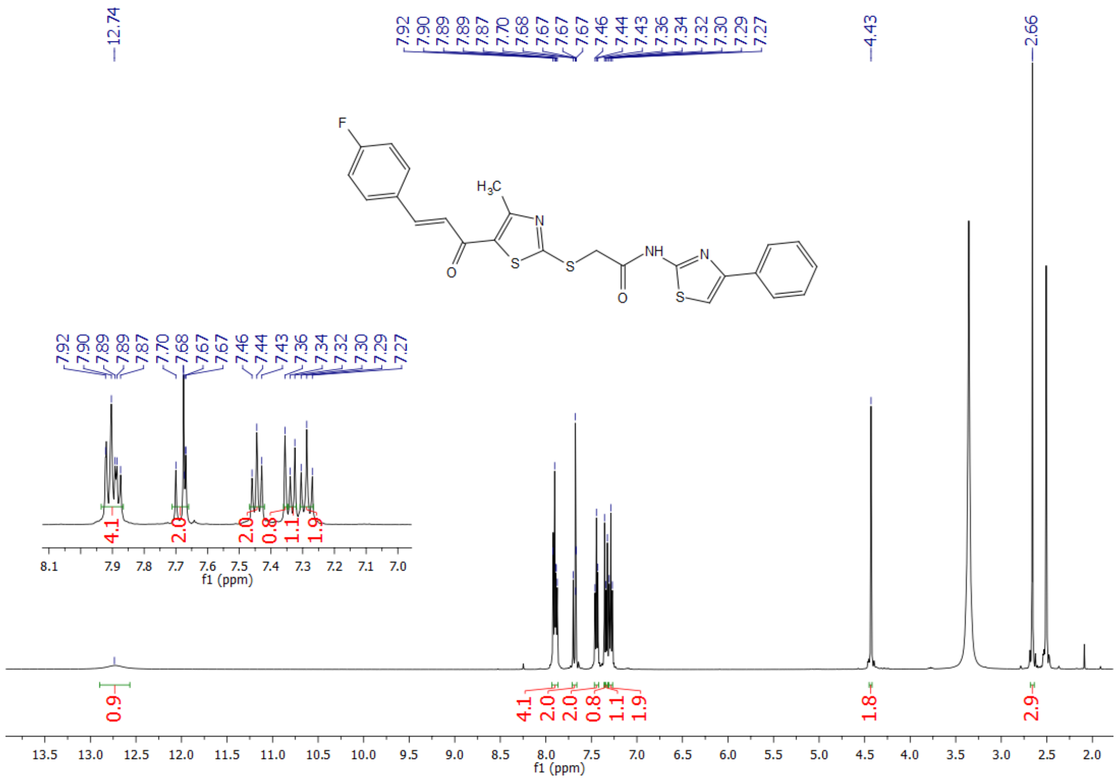

Figure S5. ^{1}H NMR (400 MHz, DMSO-d_6) spectrum of **10c**

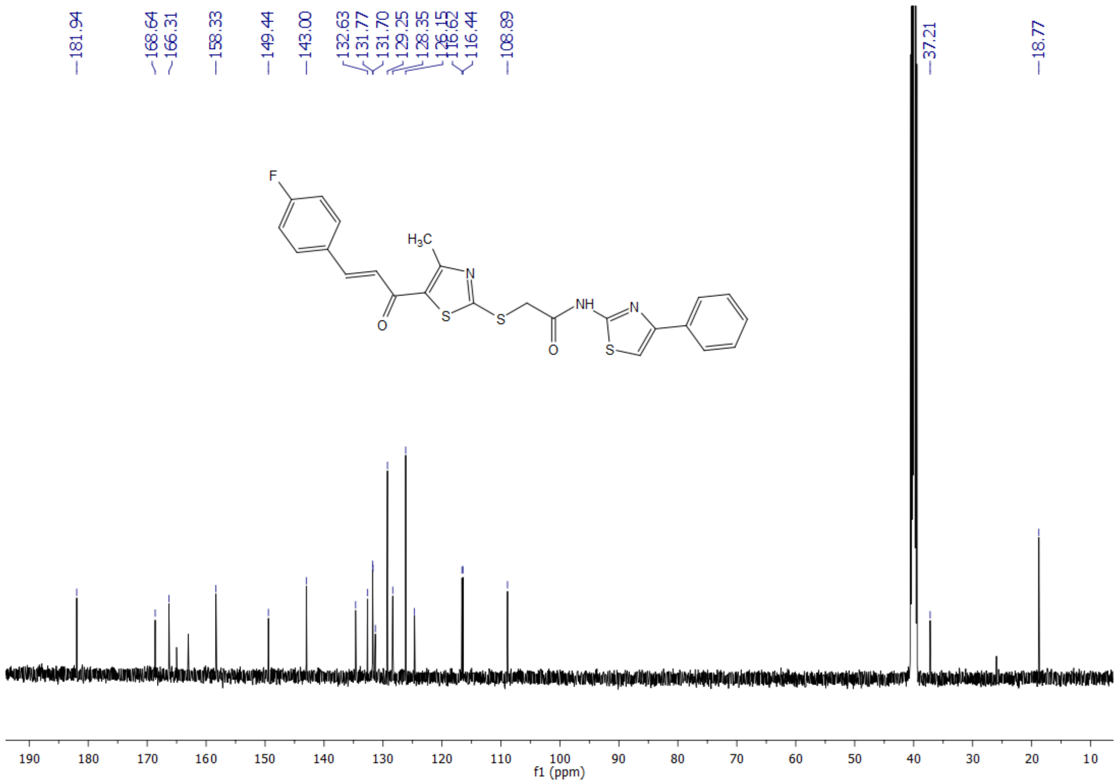

Figure S6. ^{13}C NMR (100 MHz, DMSO-d_6) spectrum of **10c**

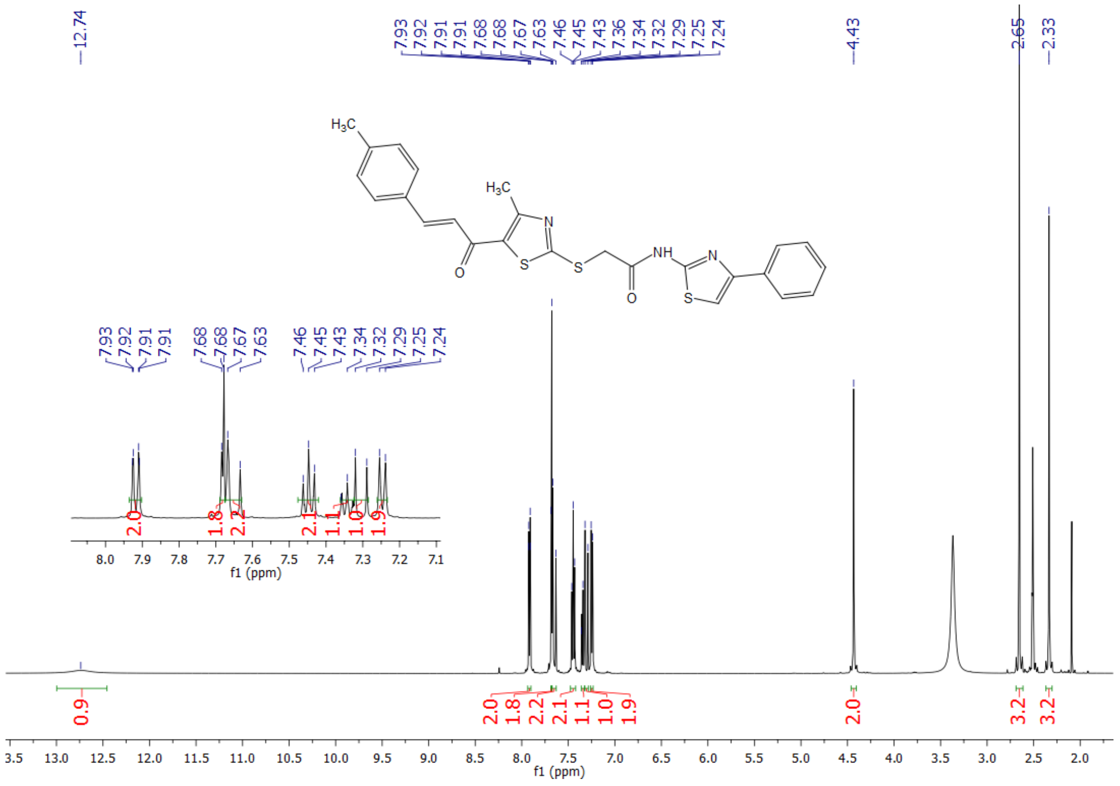

Figure S7. ^{1}H NMR (400 MHz, DMSO-d_6) spectrum of **10d**

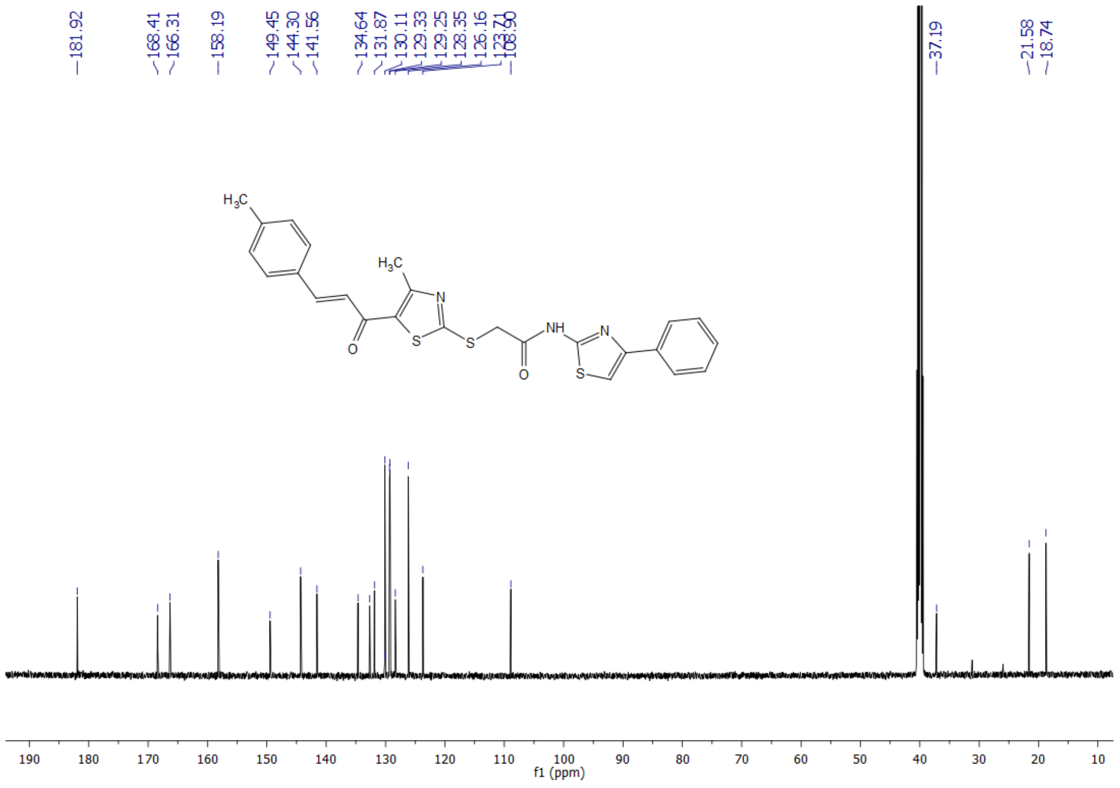

Figure S8. ^{13}C NMR (100 MHz, DMSO-d_6) spectrum of **10d**

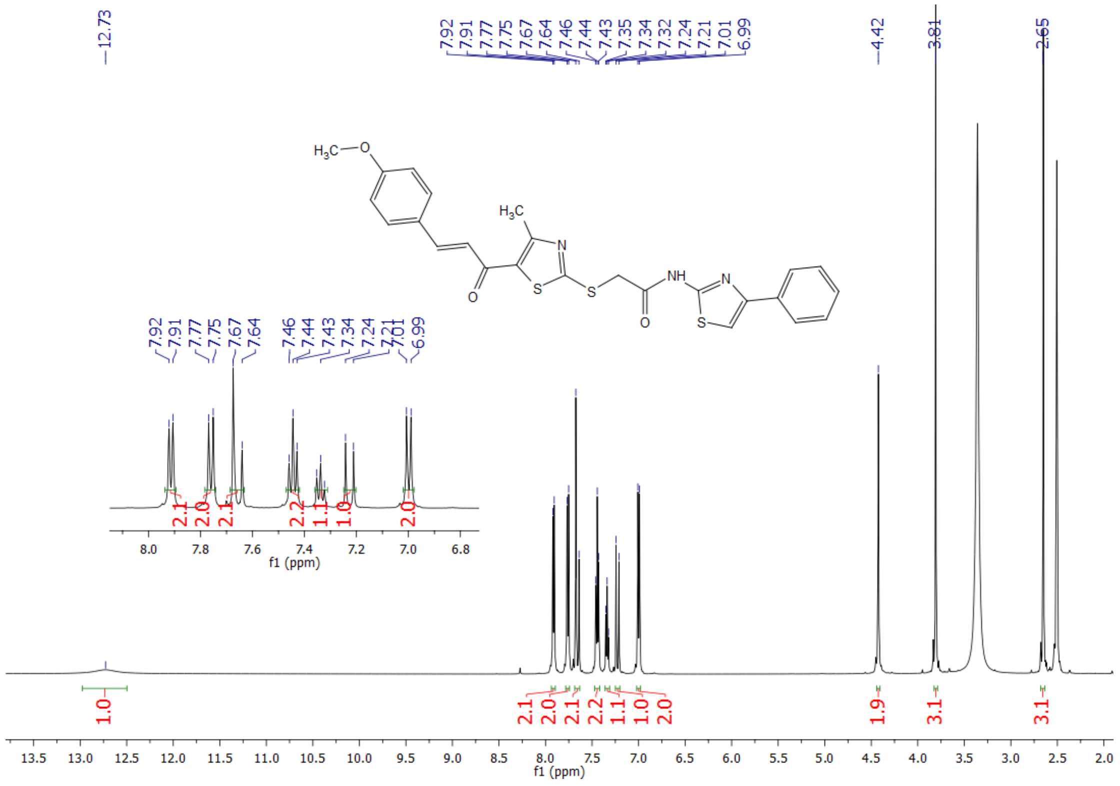

Figure S9. ^{1}H NMR (400 MHz, DMSO-d_6) spectrum of **10e**

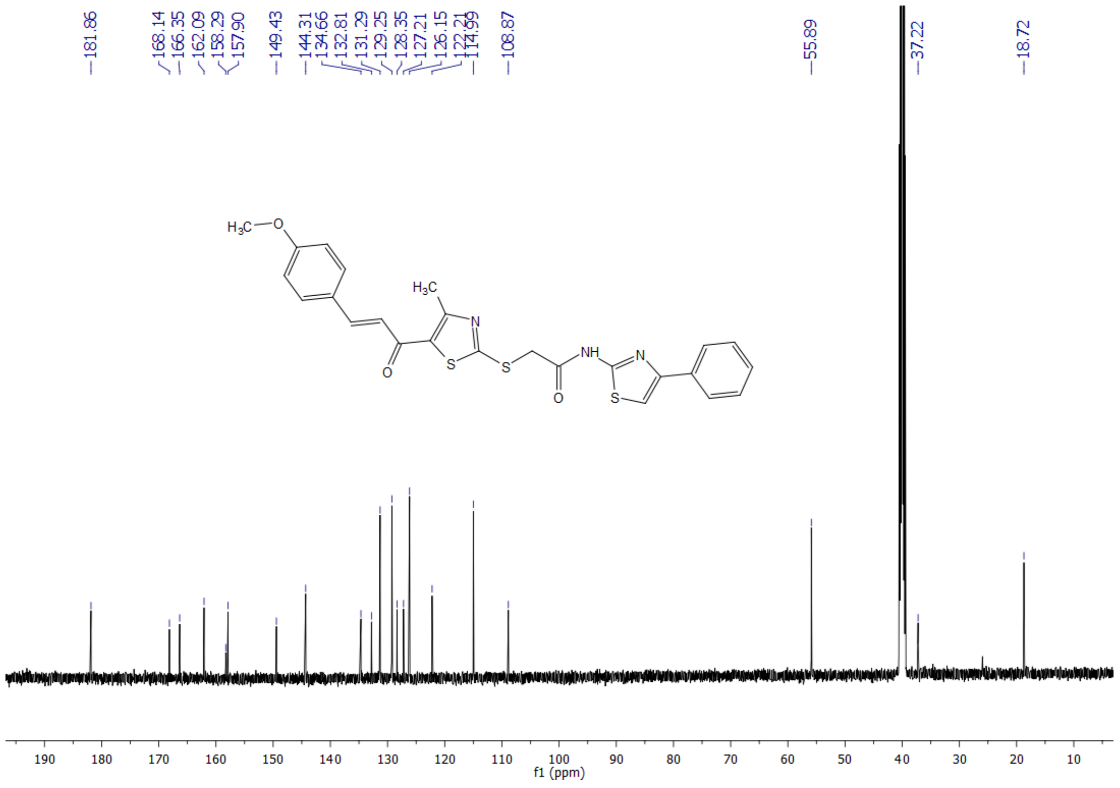

Figure S10. ^{13}C NMR (100 MHz, DMSO-d_6) spectrum of **10e**

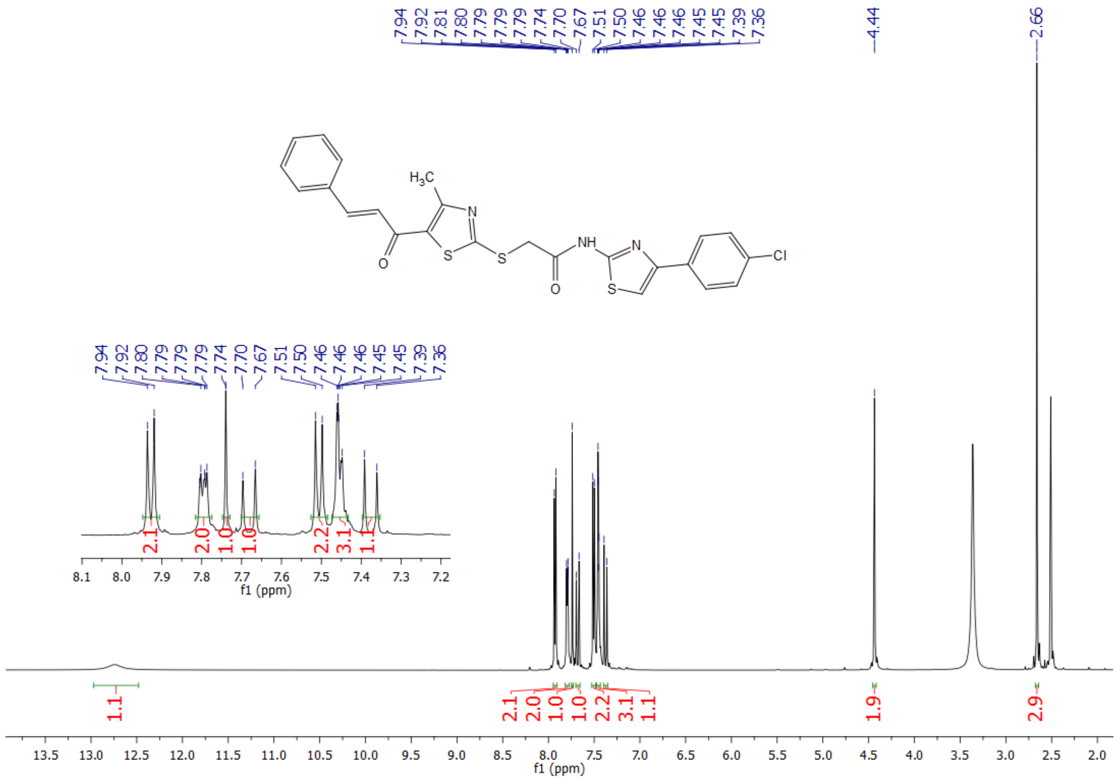

Figure S11. ^{1}H NMR (400 MHz, DMSO-*d*6) spectrum of **10f**

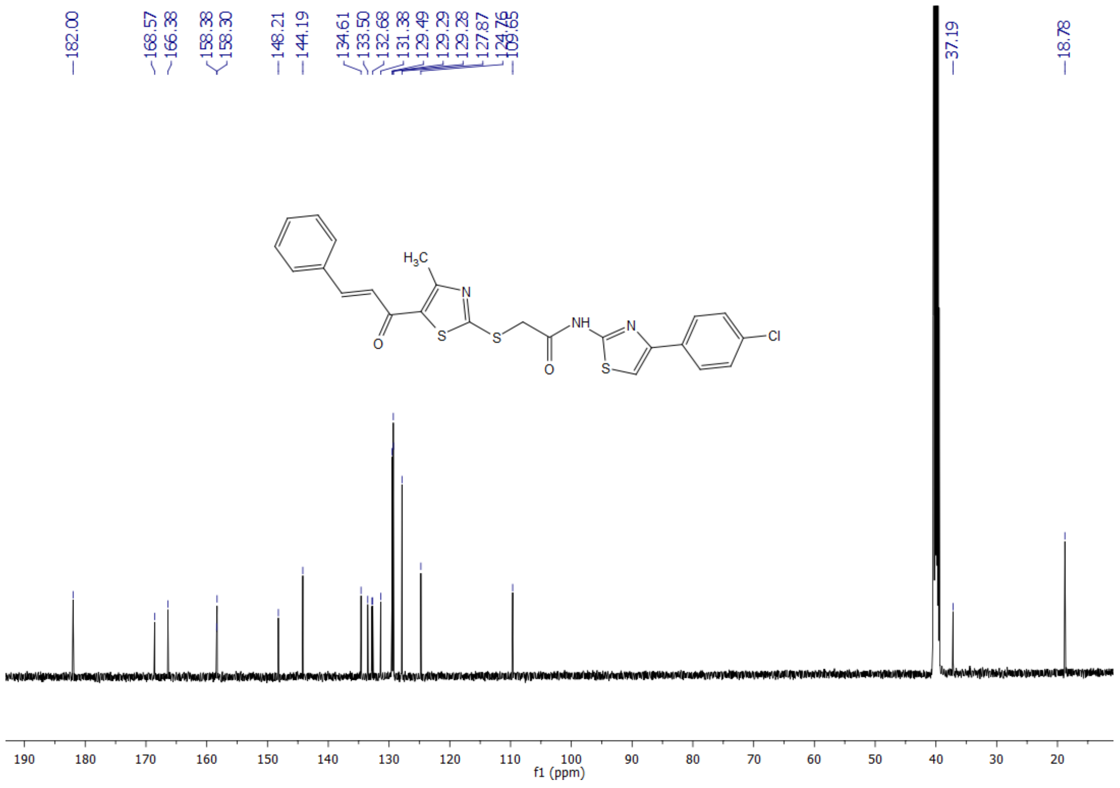

Figure S12. ^{13}C NMR (100 MHz, DMSO-*d*6) spectrum of **10f**

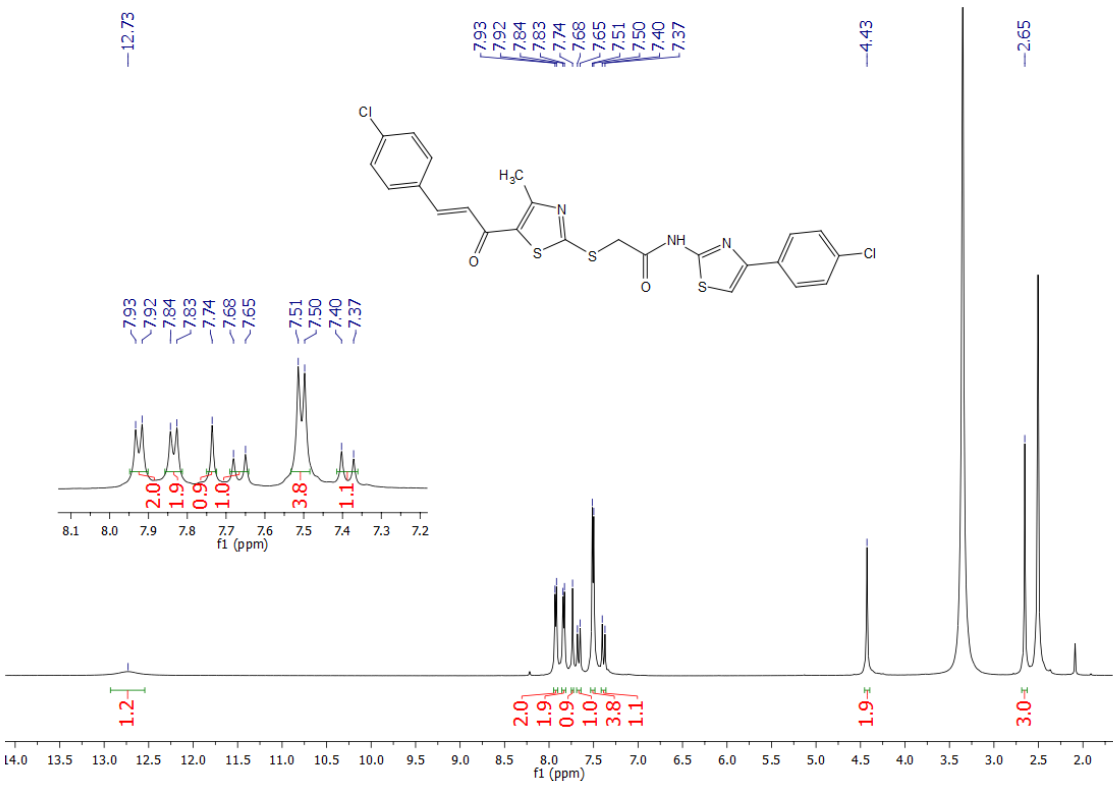

Figure S13. ^{1}H NMR (400 MHz, DMSO-d_6) spectrum of **10g**

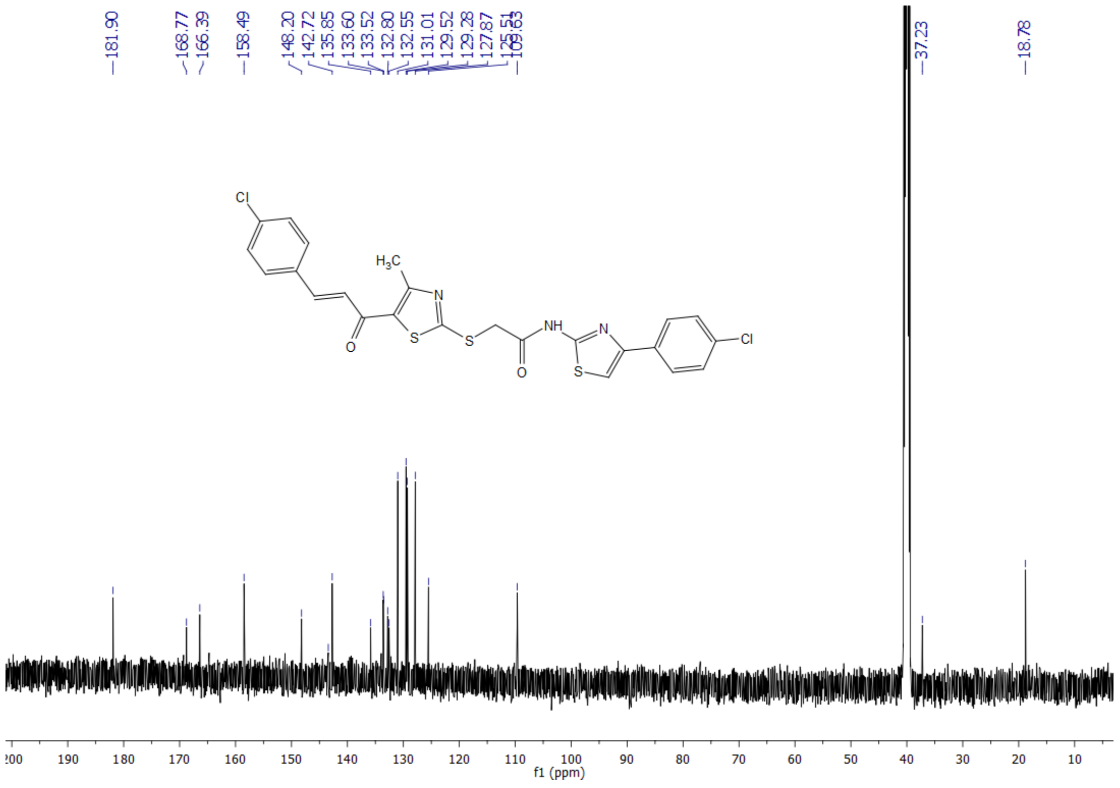

Figure S14. ^{13}C NMR (100 MHz, DMSO-d_6) spectrum of **10g**

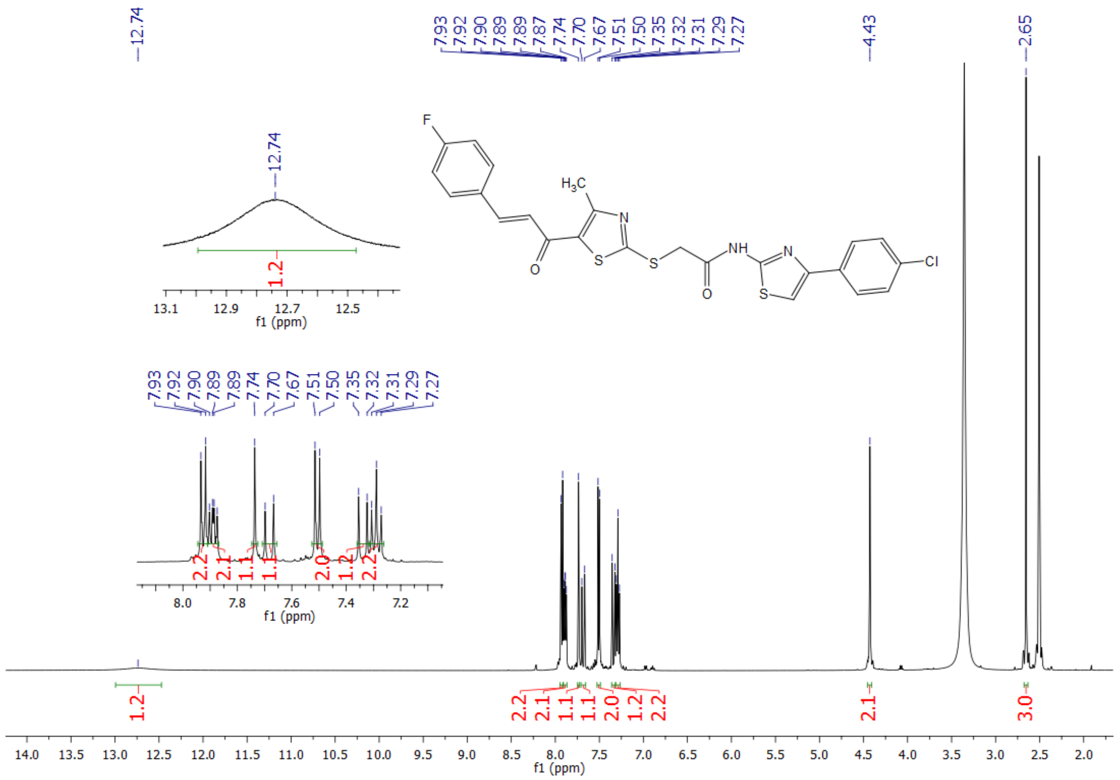

Figure S15. ^{1}H NMR (400 MHz, DMSO-d_6) spectrum of **10h**

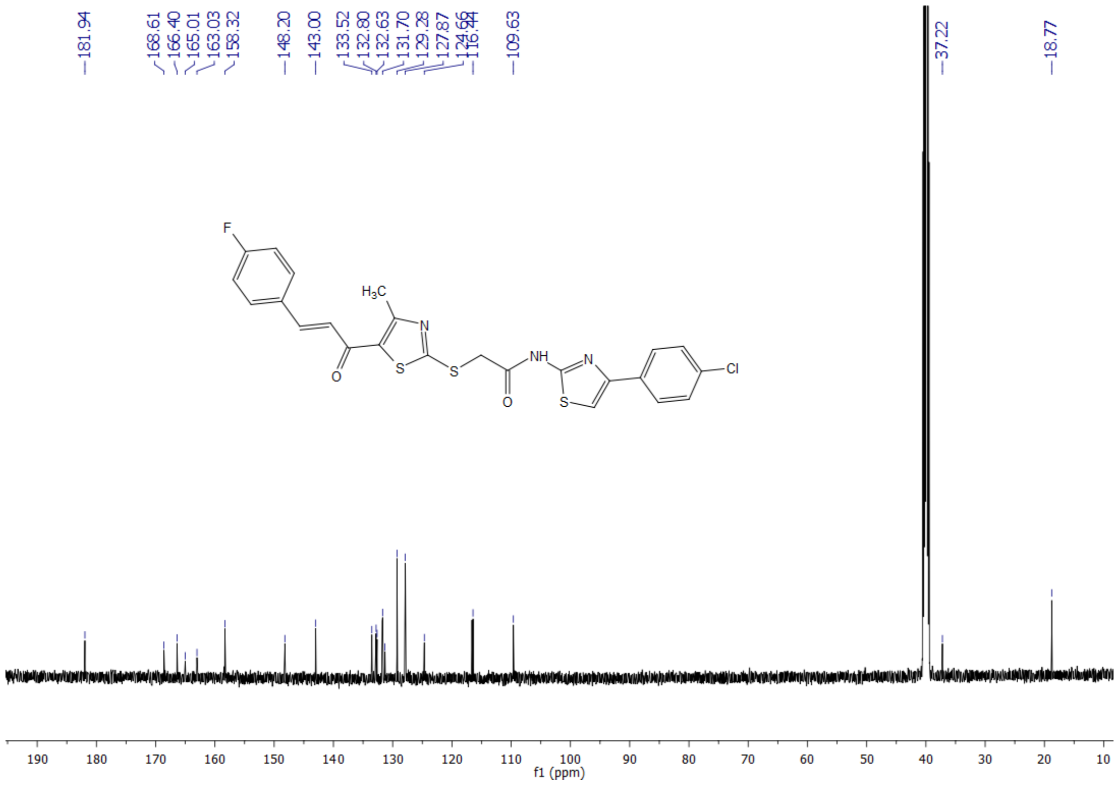

Figure S16. ^{13}C NMR (100 MHz, DMSO-d_6) spectrum of **10h**

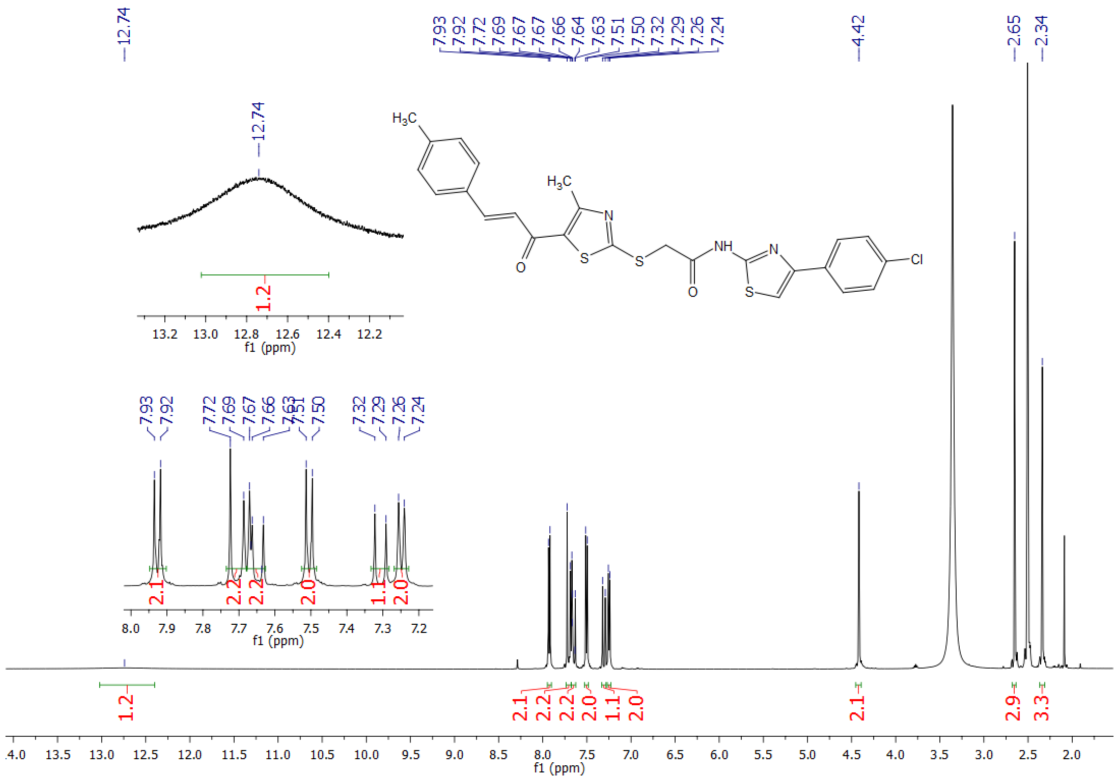

Figure S17. ^{1}H NMR (400 MHz, DMSO-d_6) spectrum of **10i**

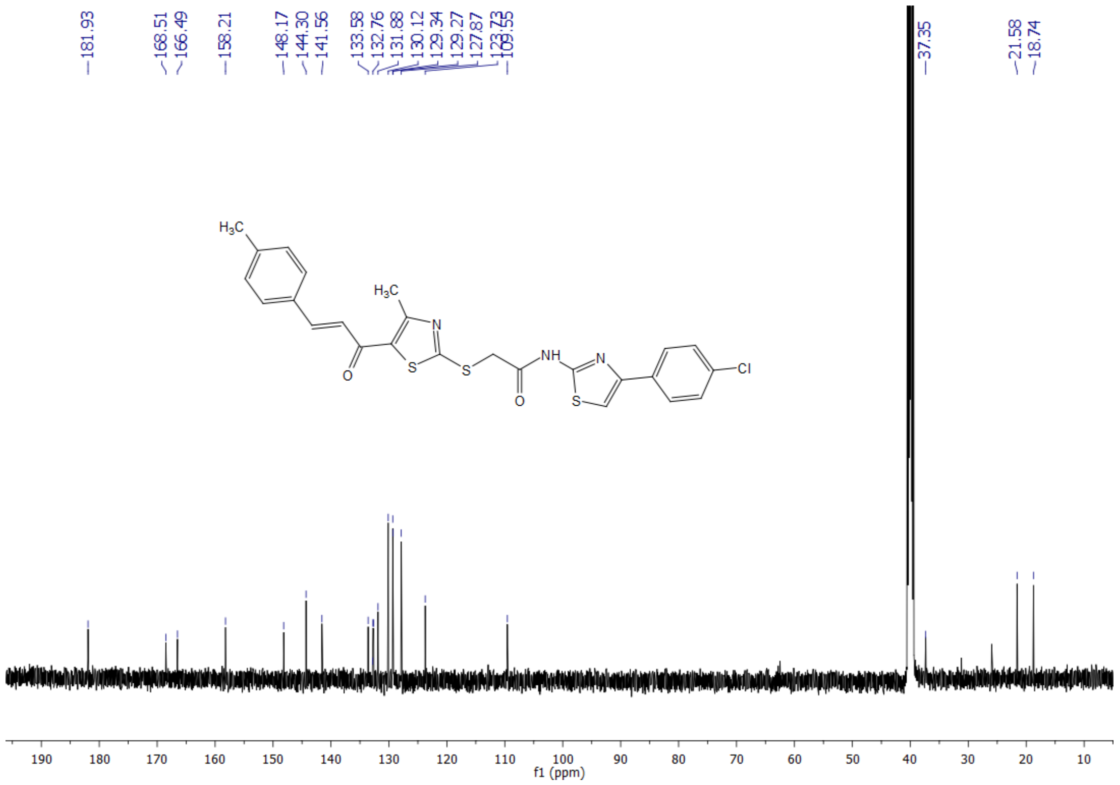

Figure S18. ^{13}C NMR (100 MHz, DMSO-d_6) spectrum of **10i**

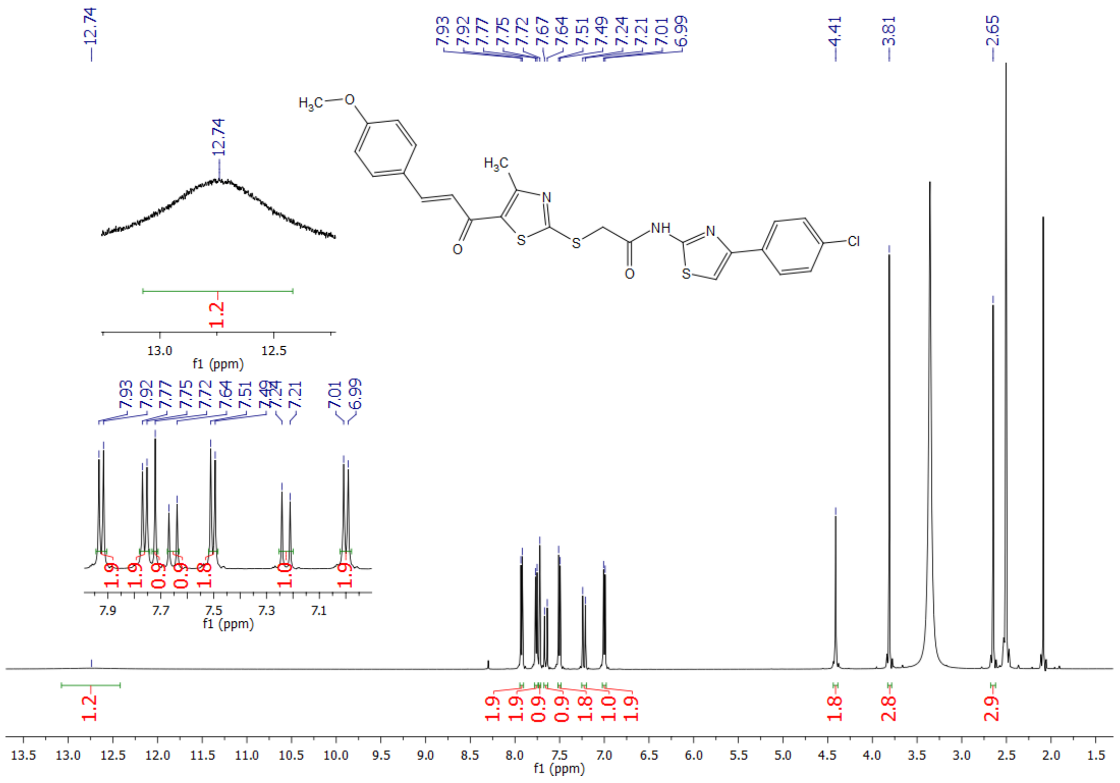

Figure S19. ^{1}H NMR (400 MHz, DMSO-d_6) spectrum of **10j**

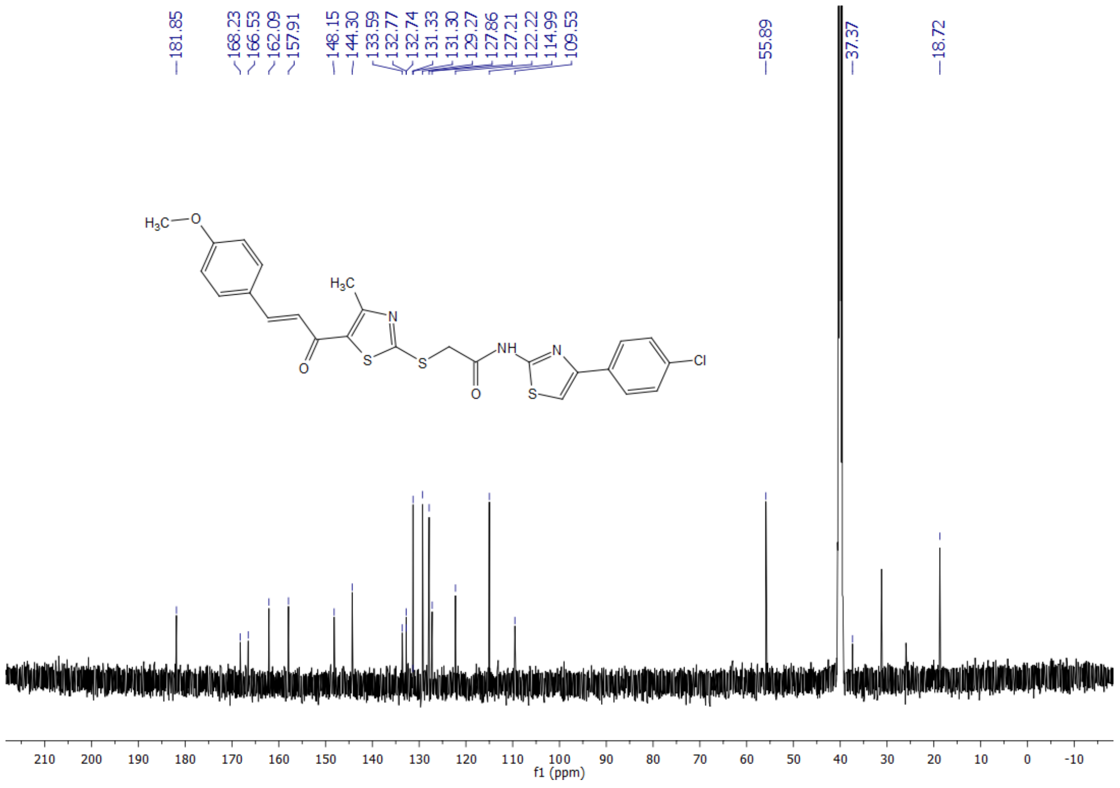

Figure S20. ^{13}C NMR (100 MHz, DMSO-d_6) spectrum of **10j**

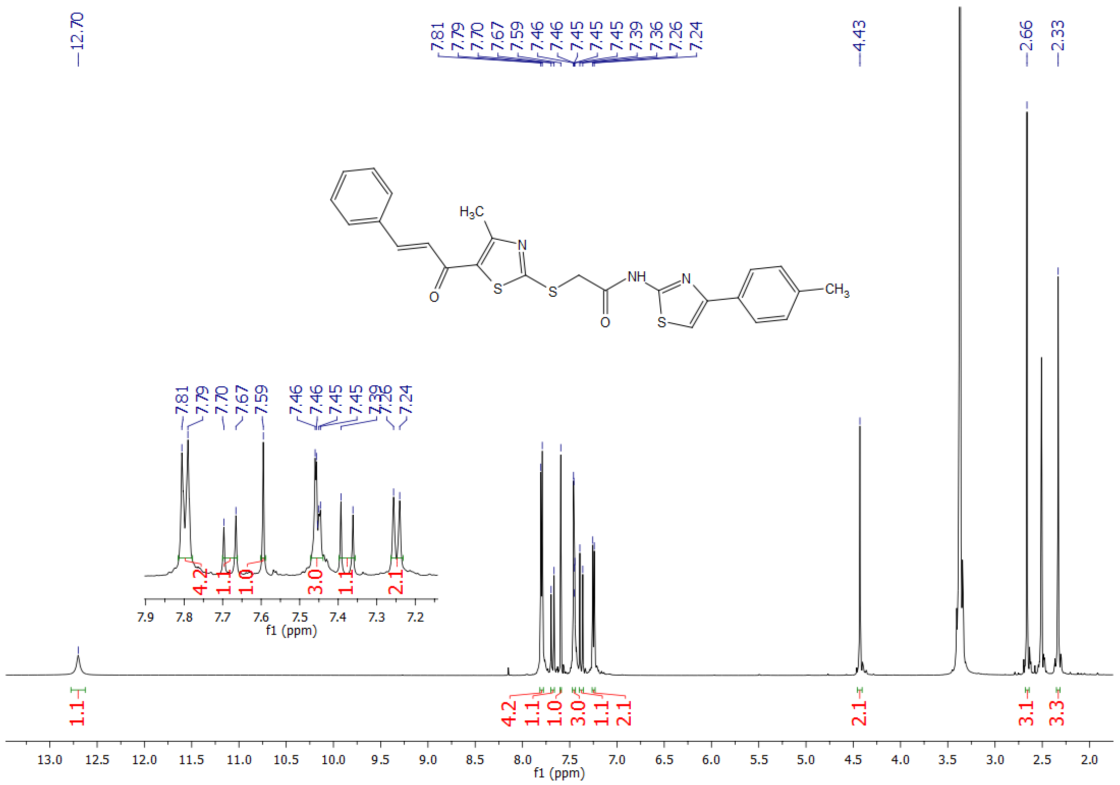

Figure S21. ^{1}H NMR (400 MHz, DMSO-d_6) spectrum of **10k**

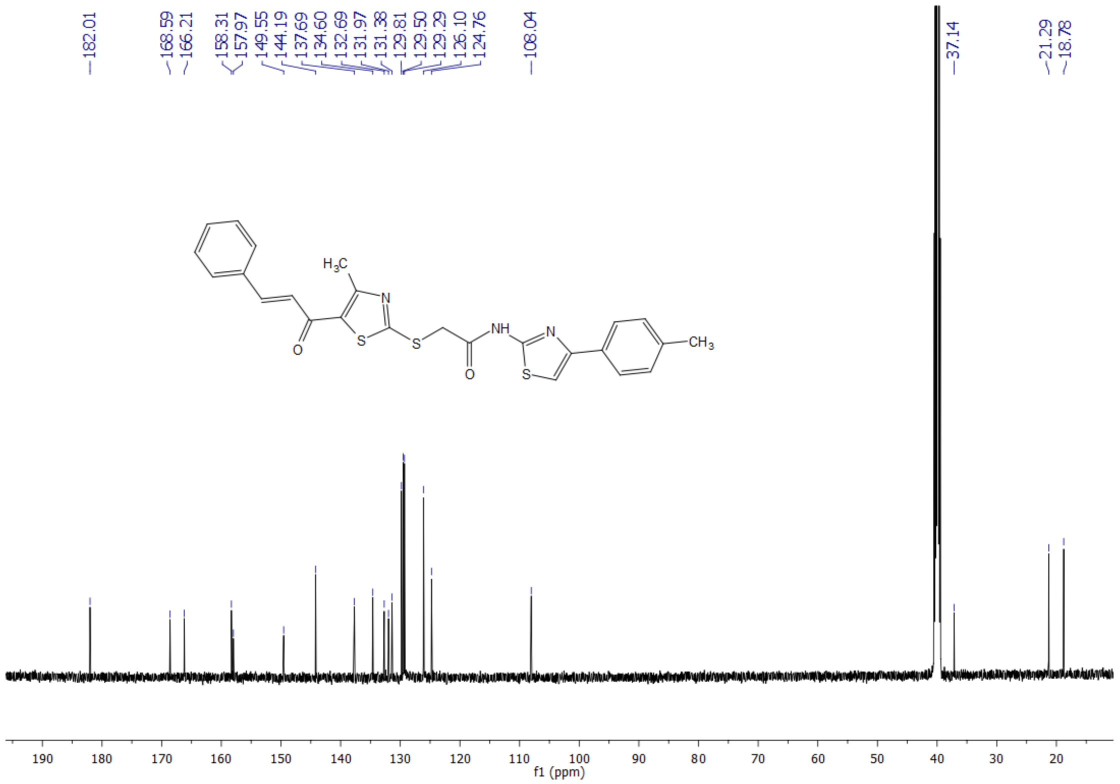

Figure S22. ^{13}C NMR (100 MHz, DMSO-d_6) spectrum of **10k**

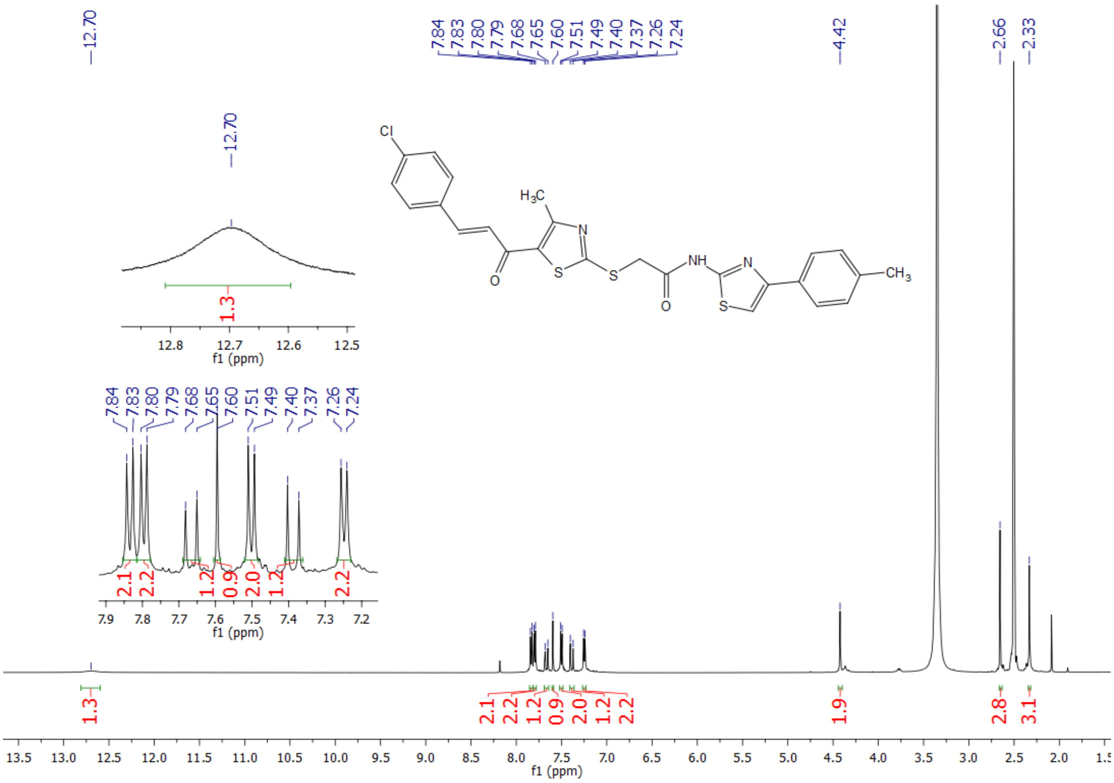

Figure S23. ^{1}H NMR (400 MHz, DMSO-d_6) spectrum of **10l**

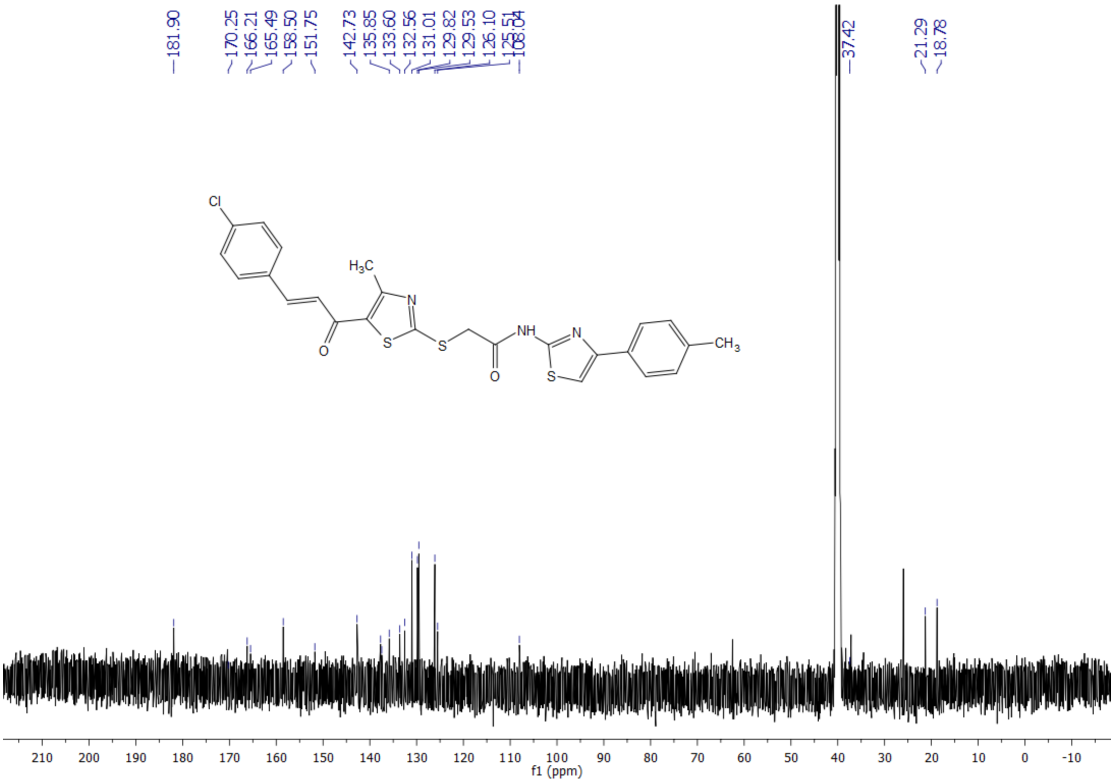

Figure S24. ^{13}C NMR (100 MHz, DMSO-d_6) spectrum of **10l**

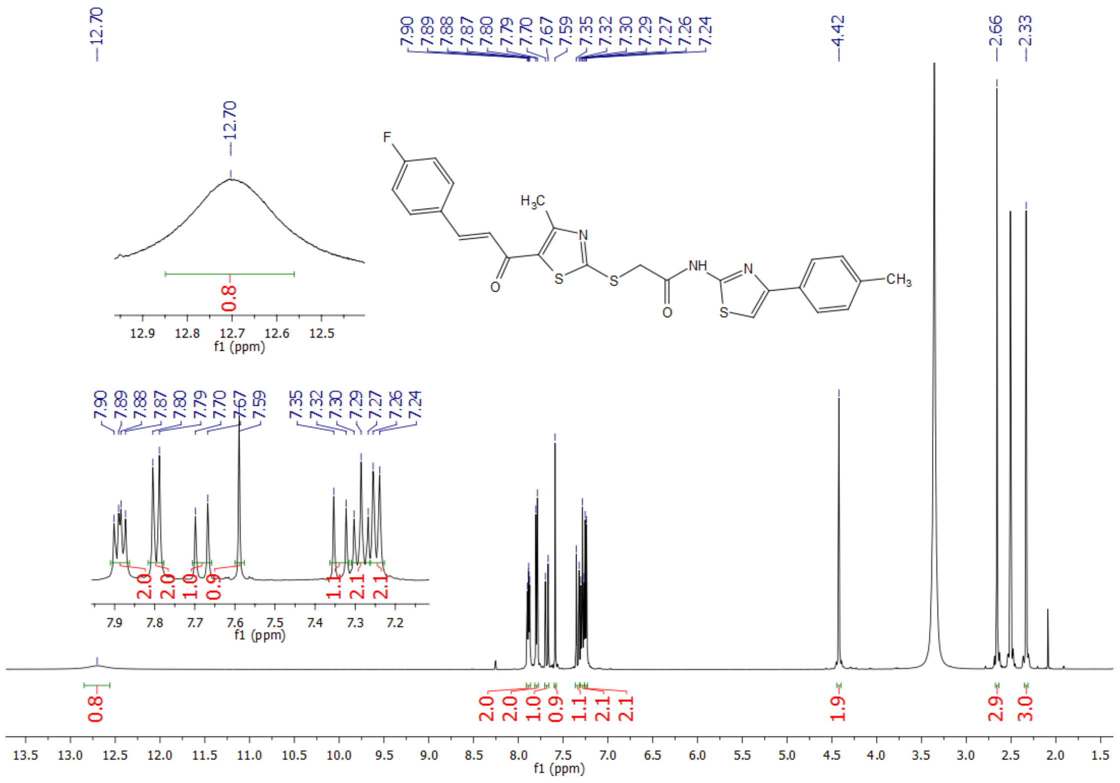

Figure S25. ^{1}H NMR (400 MHz, DMSO-d_6) spectrum of **10m**

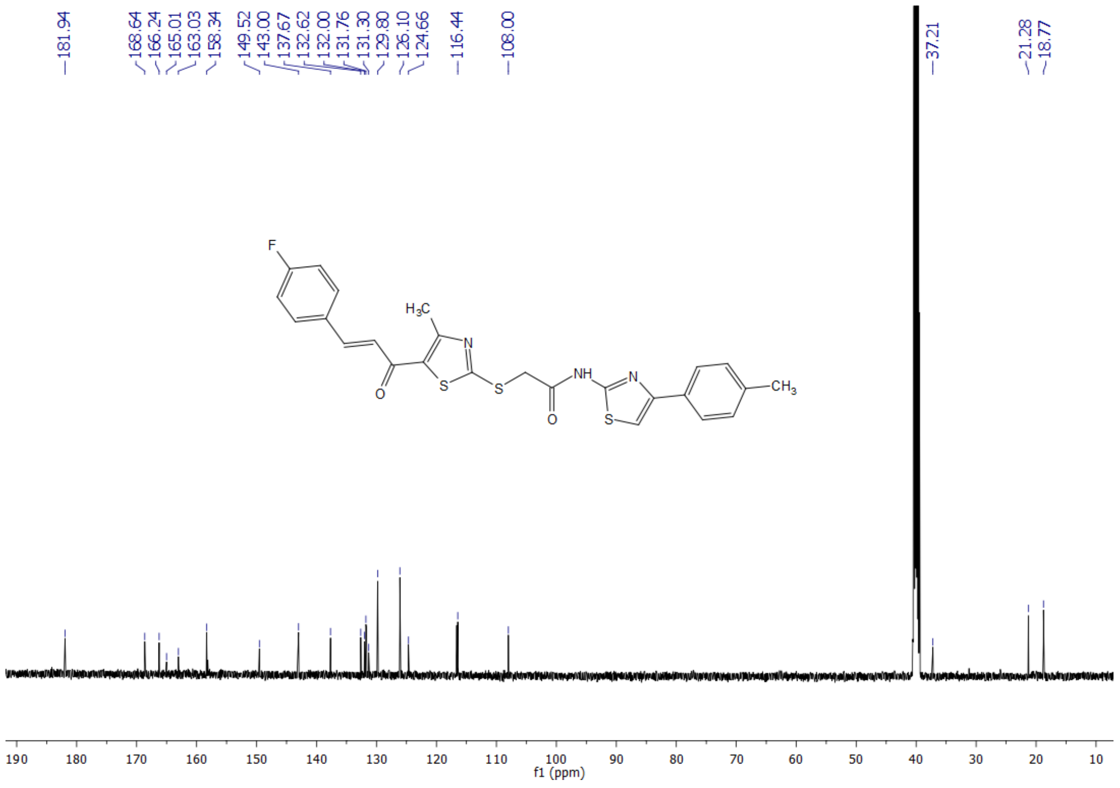

Figure S26. ^{13}C NMR (100 MHz, DMSO-d_6) spectrum of **10m**

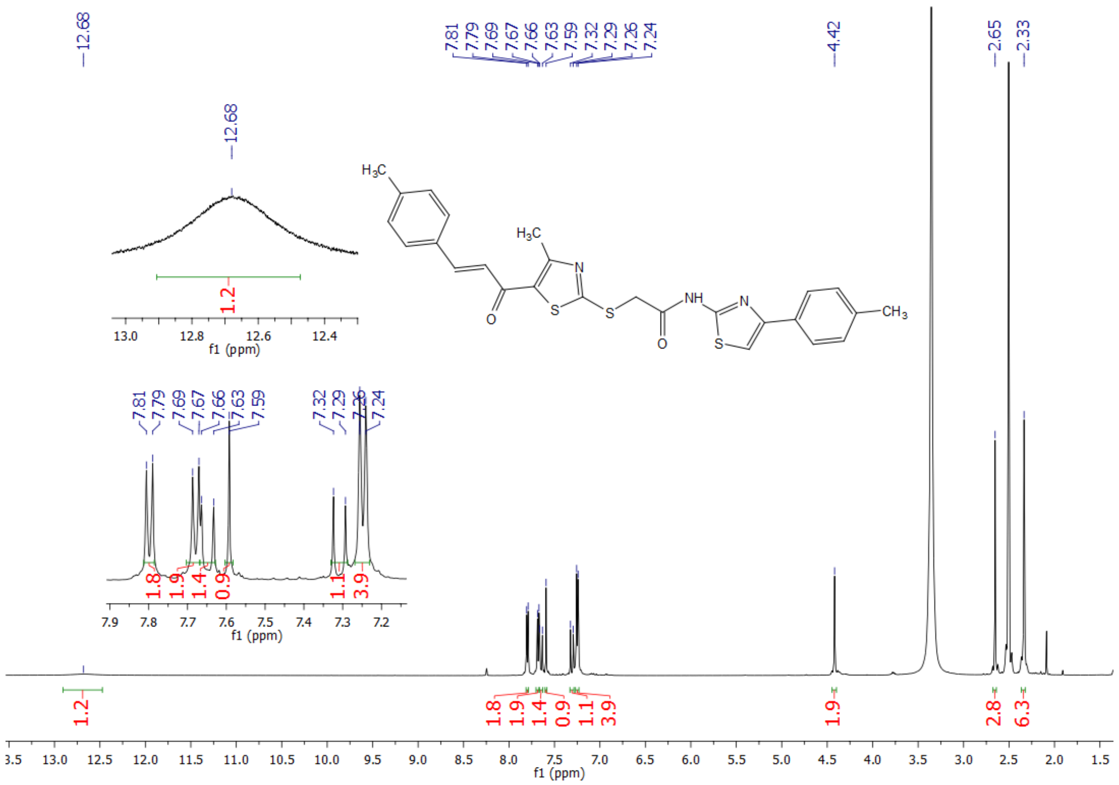

Figure S27. ^{1}H NMR (400 MHz, DMSO-d_6) spectrum of **10n**

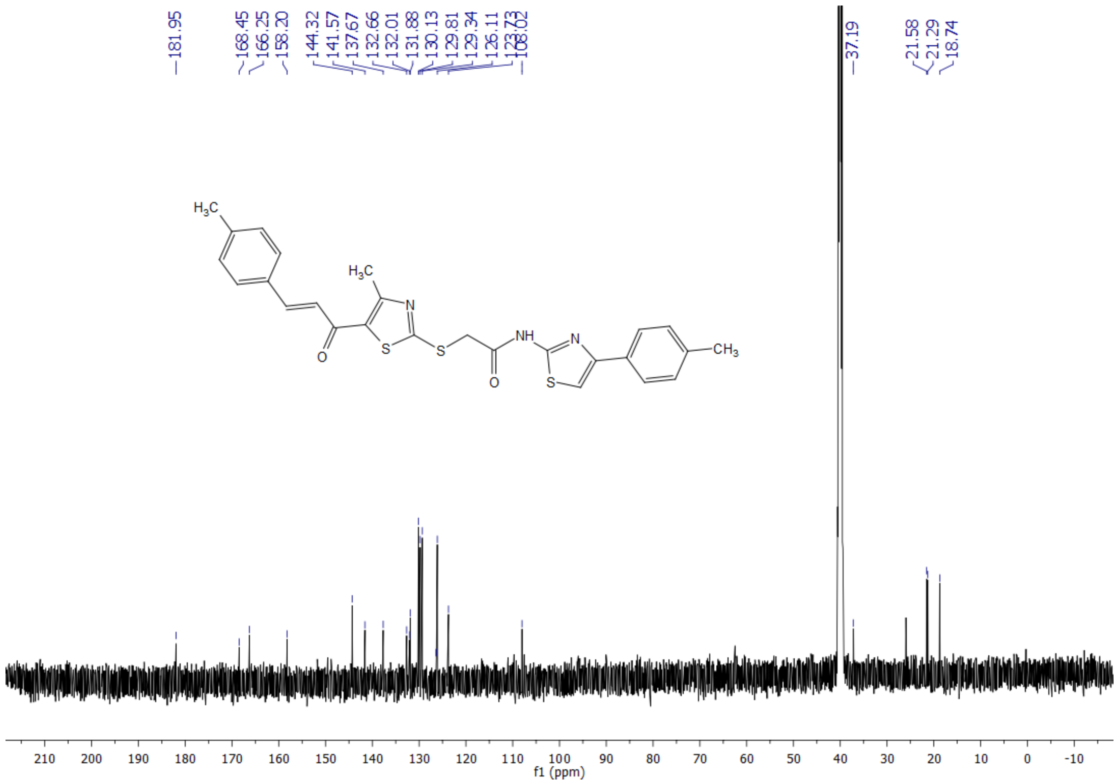

Figure S28. ^{13}C NMR (100 MHz, DMSO-d_6) spectrum of **10n**

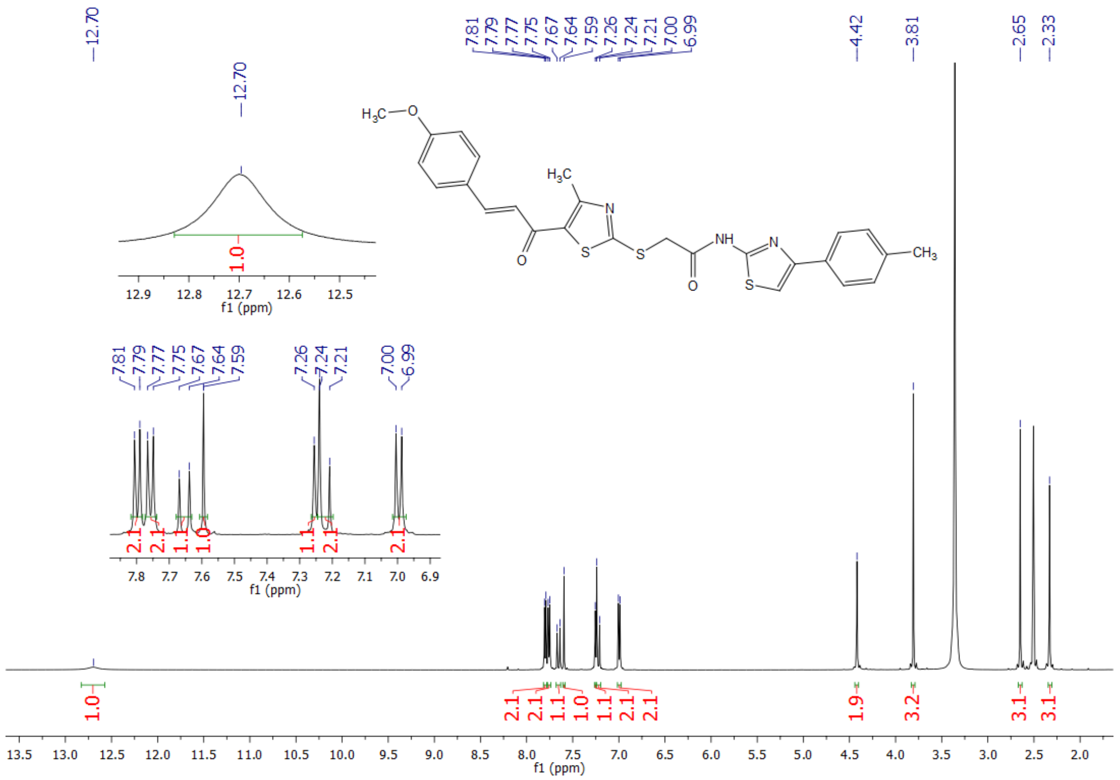

Figure S29. ^{1}H NMR (400 MHz, DMSO-d_6) spectrum of **10o**

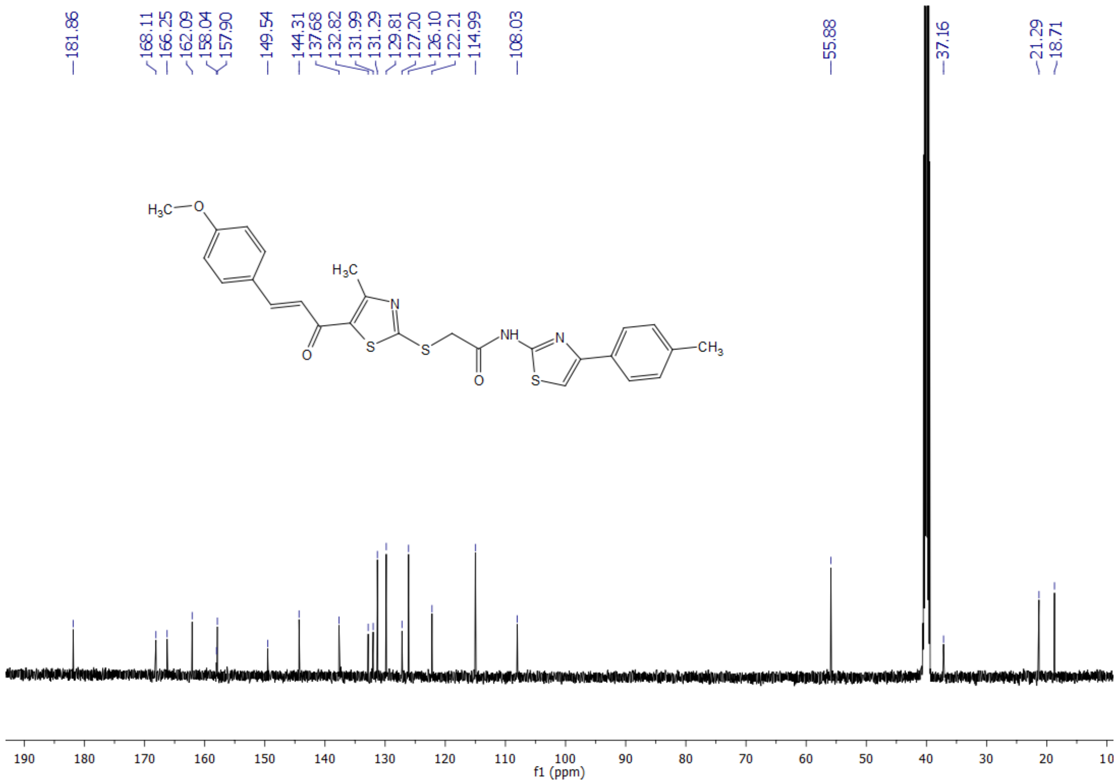

Figure S30. ^{13}C NMR (100 MHz, DMSO-d_6) spectrum of **10o**

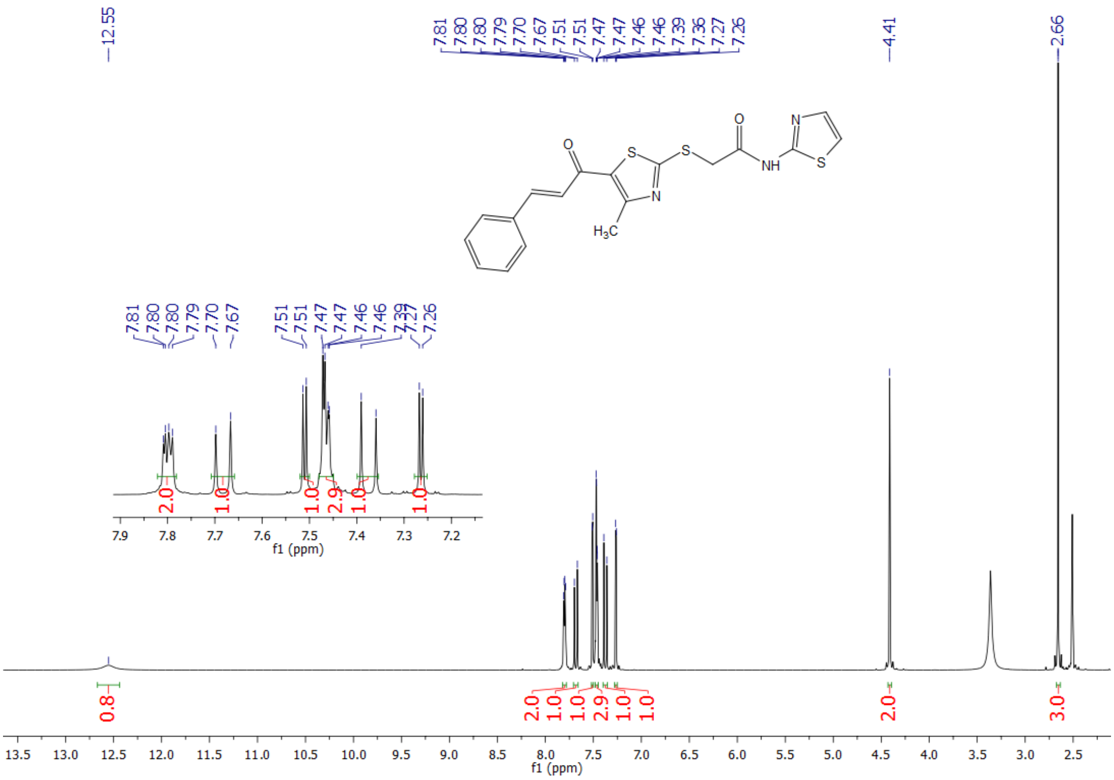

Figure S31. ^{1}H NMR (400 MHz, DMSO-d_6) spectrum of **13a**

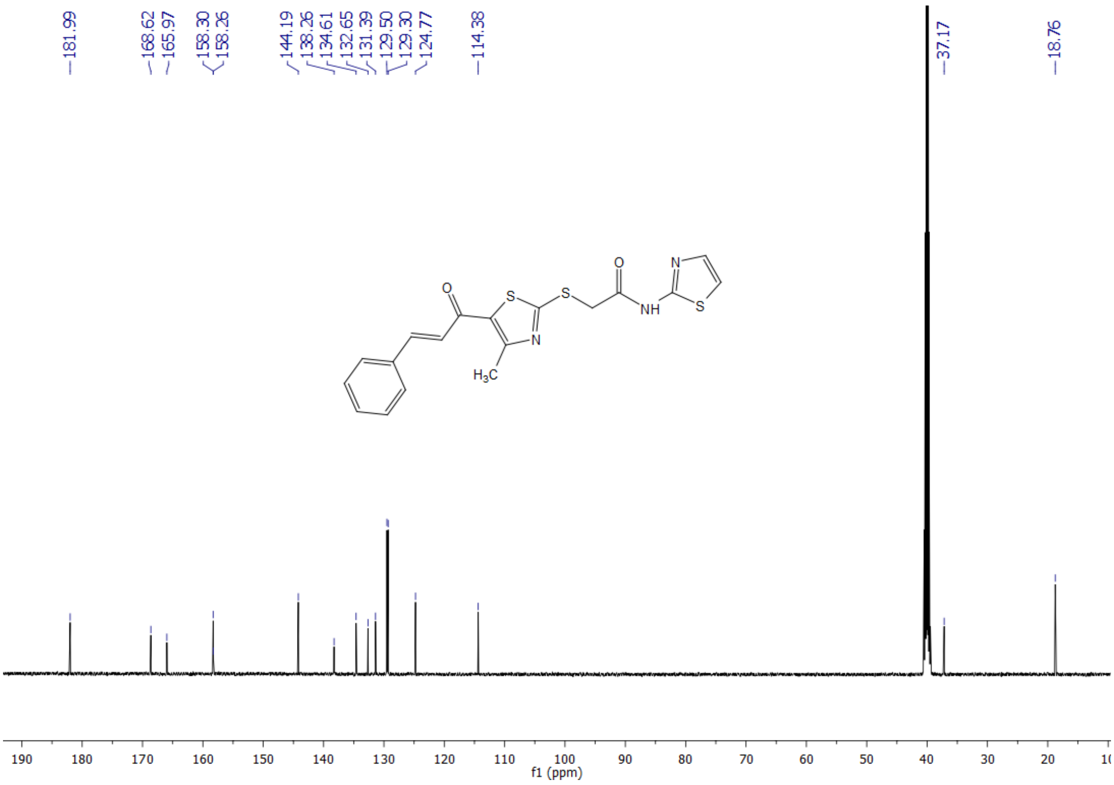

Figure S32. ^{13}C NMR (100 MHz, DMSO-d_6) spectrum of **13a**

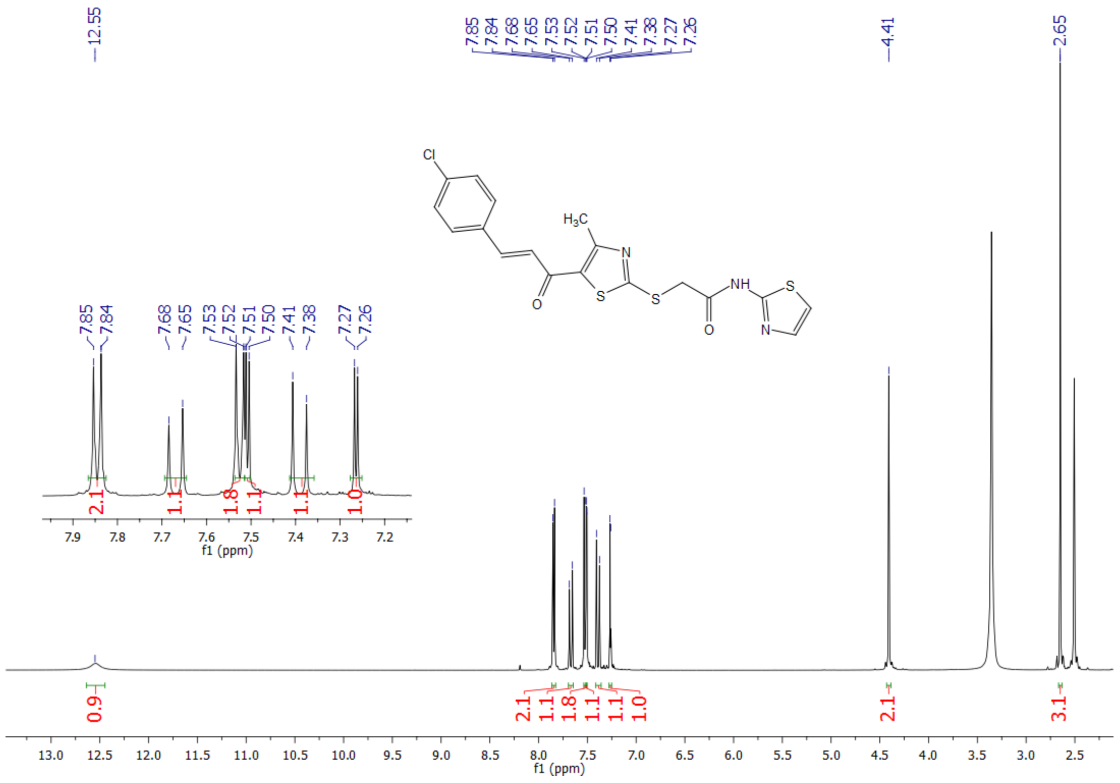

Figure S33. ^{1}H NMR (400 MHz, DMSO-d_6) spectrum of **13b**

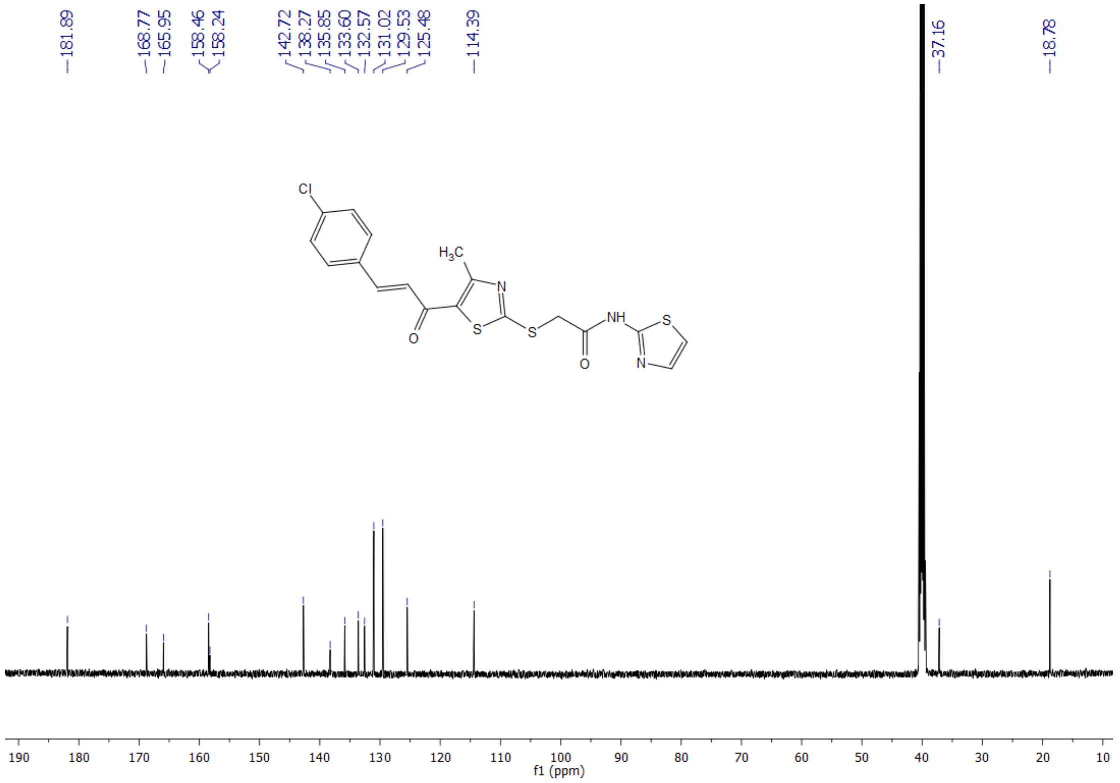

Figure S34. ^{13}C NMR (100 MHz, DMSO-d_6) spectrum of **13b**

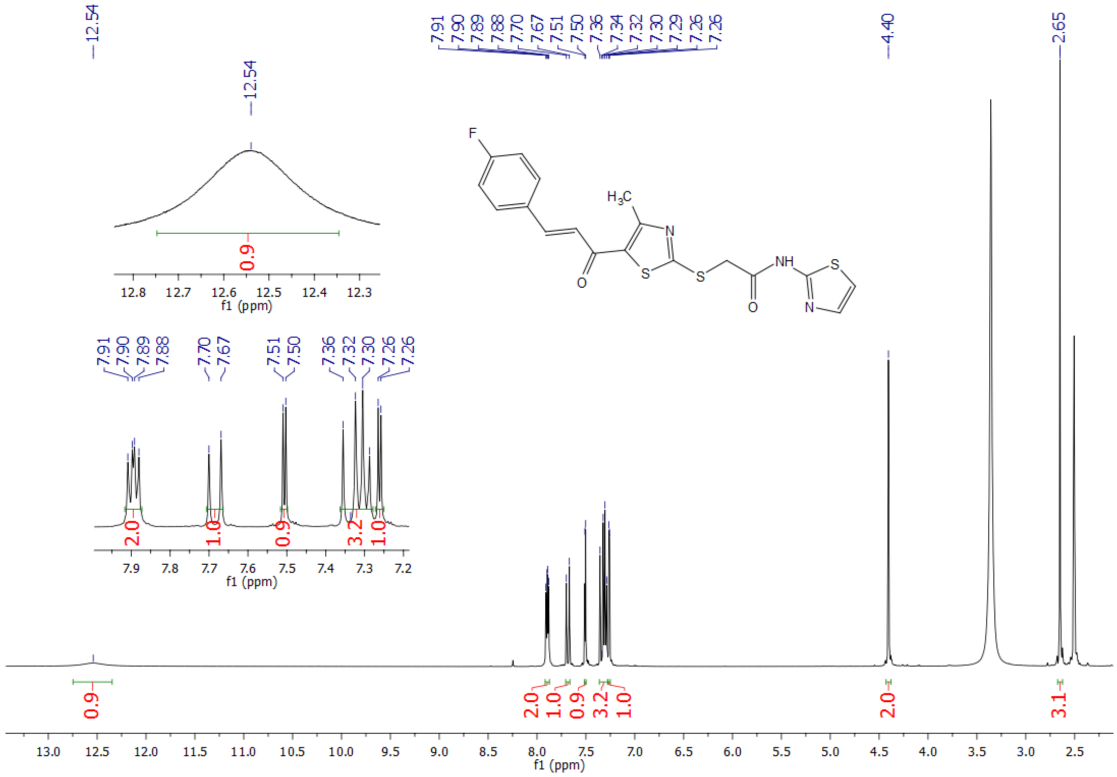

Figure S35. ^{1}H NMR (400 MHz, DMSO-d_6) spectrum of **13c**

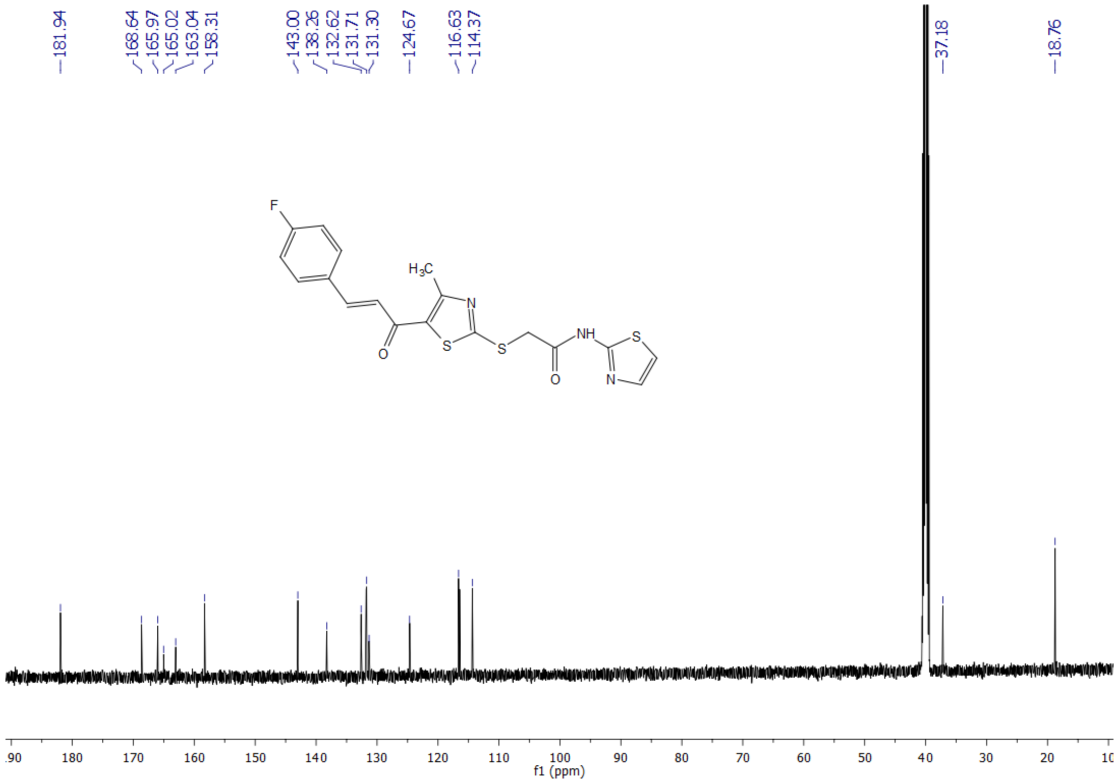

Figure S36. ^{13}C NMR (100 MHz, DMSO-d_6) spectrum of **13c**

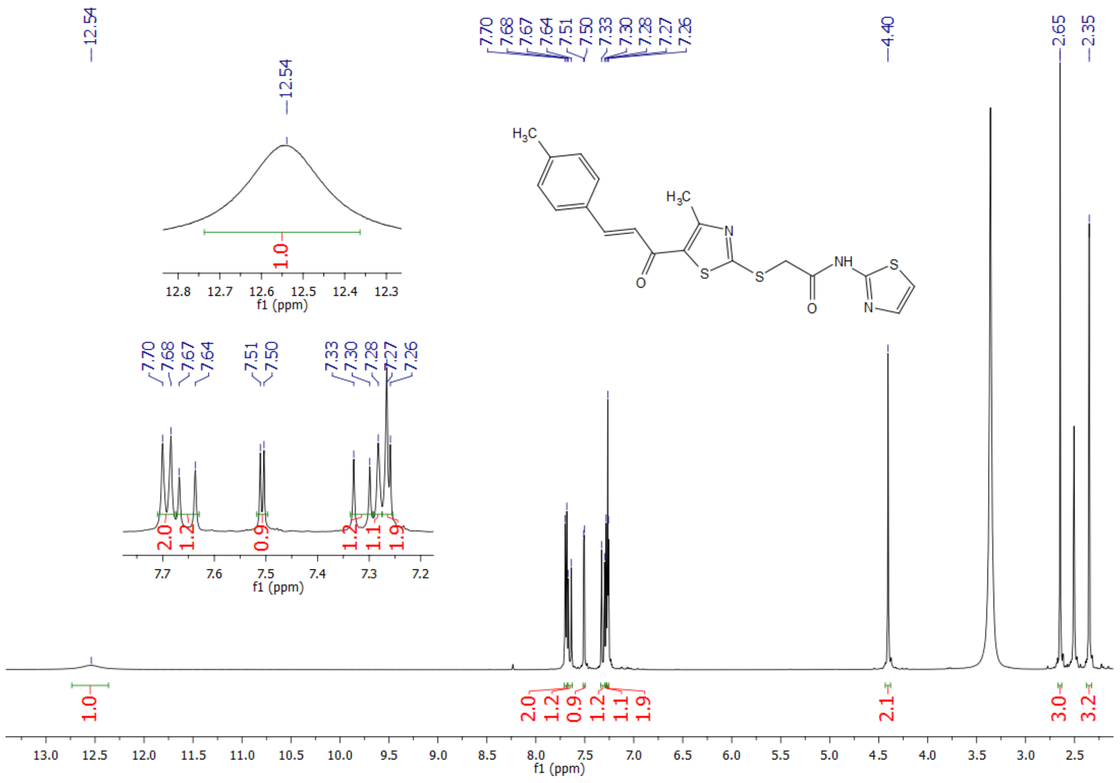

Figure S37. ^{1}H NMR (400 MHz, DMSO-d_6) spectrum of **13d**

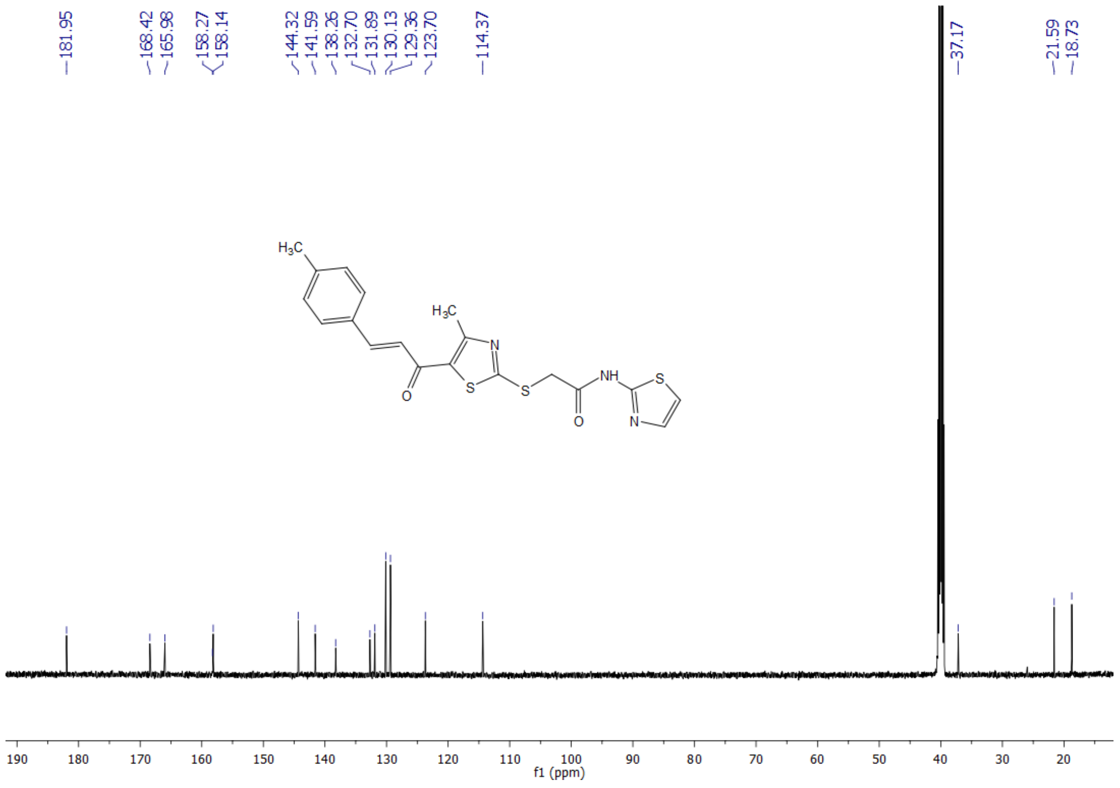

Figure S38. ^{13}C NMR (100 MHz, DMSO-d_6) spectrum of **13d**

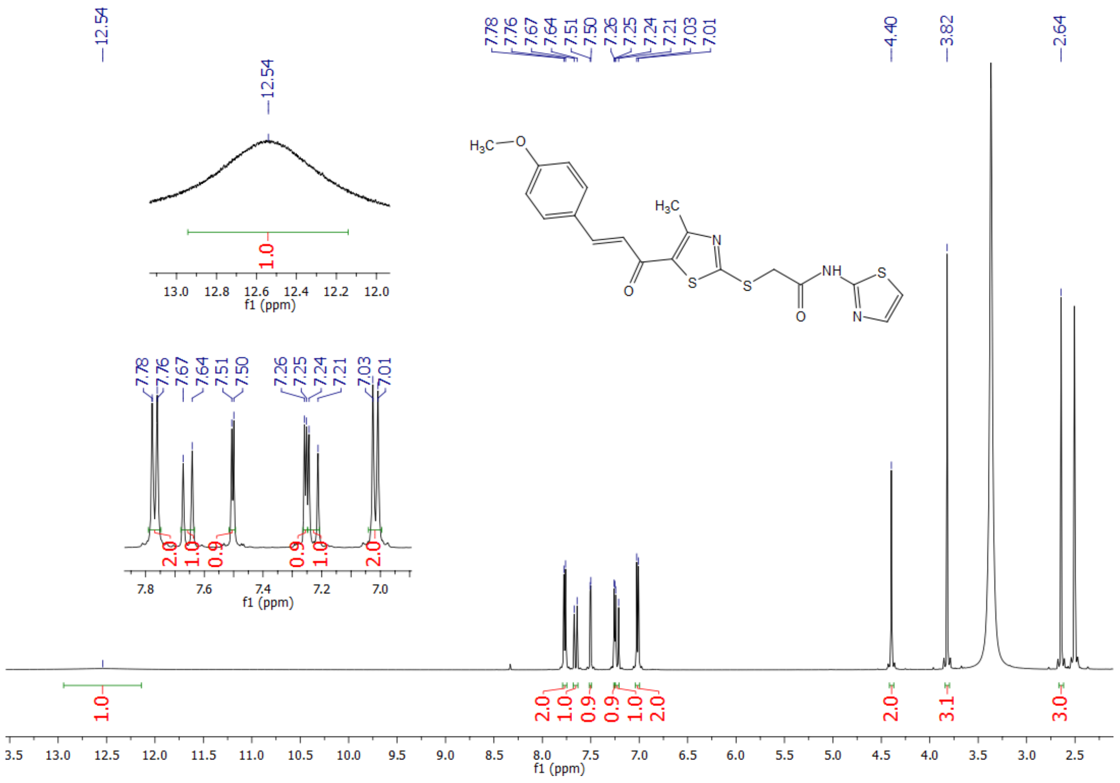

Figure S39. ^{1}H NMR (400 MHz, DMSO-d_6) spectrum of **13e**

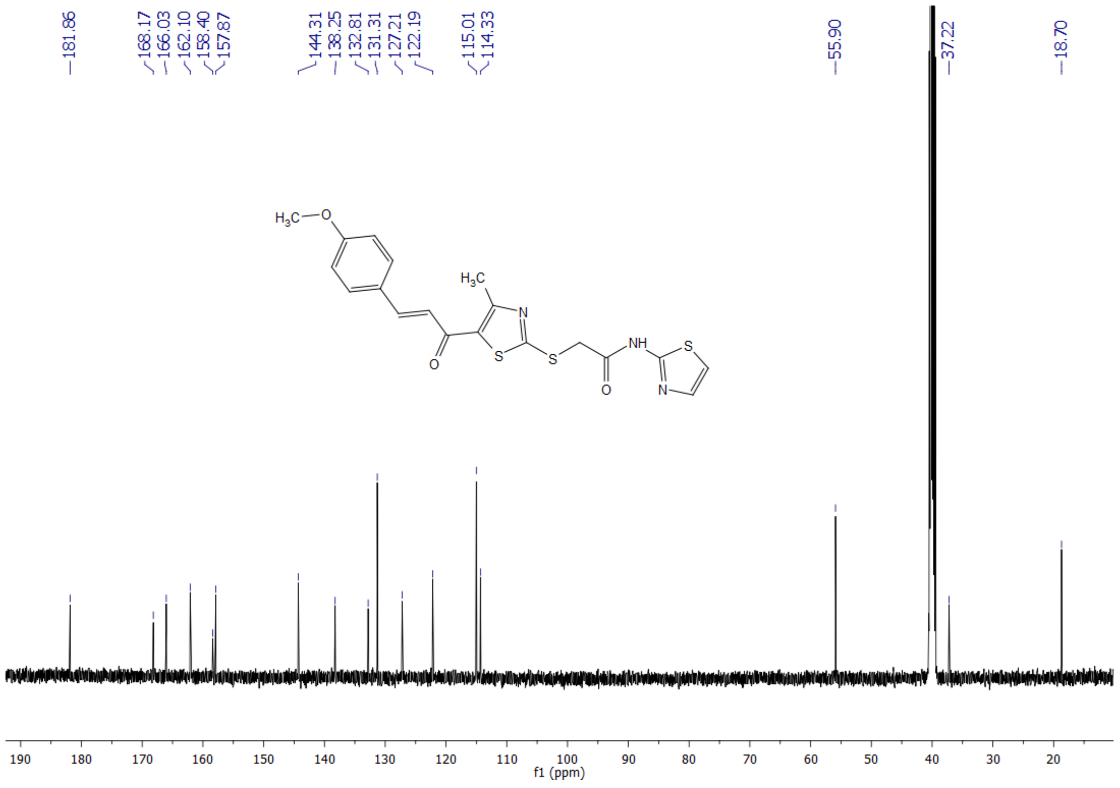

Figure S40. ^{13}C NMR (100 MHz, DMSO-d_6) spectrum of **13e**

Appendix A

4. Experimental

4.1. Chemistry

General Information

All reagents and solvents were of general purpose or analytical grade and purchased from Sigma Aldrich Ltd, Fisher Scientific, Fluka and Acros. 1H and 13C NMR spectra were recorded with a Bruker Avance III spectrometer operating at 400, 100 MHz respectively, with Me4Si as internal standard and DMSO-d6 as a solvent. Elemental analysis was performed by the regional center for mycology and biotechnology (Cairo, Egypt). TLC was carried out on precoated silica plates (Keisel gel 60 F254, BDH) using Hexane: Ethyl acetate, 1 : 2, v/v. Compounds were visualized by illumination under UV light (254 nm). Melting points were determined on an electrothermal instrument and are uncorrected. All solvents were dried prior to use and stored over 4 Å molecular sieves, under nitrogen. All the compounds were ≥ 95% pure.

4.2. Biological evaluation

4.2.1 Cell Viability assay (MTT assay)

MTT assay was performed to investigate the effect of the synthesized compounds on mammary epithelial cells (MCF-10A). The cells were propagated in medium consisting of Ham's F-12 medium/ Dulbecco's modified Eagle's medium (DMEM) (1:1) supplemented with 10% foetal calf serum, 2 mM glutamine, insulin (10 μg/mL), hydrocortisone (500 ng/mL) and epidermal growth factor (20 ng/mL). Trypsin ethylenediamine tetra acetic acid (EDTA) was used to passage the cells after every 2-3 days. 96-well flat-bottomed cell culture plates were used to seed the cells at a density of 104 cells mL^{-1}. The medium was aspirated from all the wells of culture plates after 24 h followed by the addition of synthesized compounds (in 200 μL medium to yield a final concentration of 0.1% (v/v) dimethyl sulfoxide) into individual wells of the plates. Four wells were designated to a single compound. The plates were allowed to incubate at 37°C for 96 h. Afterwards, the medium was aspirated and 3-[4,5-dimethylthiazol-2-yl]-2,5-diphenyltetrazolium bromide (MTT) (0.4 mg/mL) in medium was added to each well and subsequently incubated for 3 h. The medium was aspirated and 150 μL dimethyl sulfoxide (DMSO) was added to each well. The plates were vortexed followed by the measurement of absorbance at 540 nm on a microplate reader. The results were presented as inhibition (%) of proliferation in contrast to controls comprising 0.1% DMSO.

4.2.2. Assay for antiproliferative effect

To explore the antiproliferative potential of compounds propidium iodide fluorescence assay was performed using different cell lines such as Panc-1 (pancreas cancer cell line), MCF-7 (breast cancer cell line), HT-29 (colon cancer cell line) and A-549 (epithelial cancer cell line), respectively. To calculate the total nuclear DNA, a fluorescent dye (propidium iodide, PI) is used which can attach to the DNA, thus offering a quick and precise technique. PI cannot pass through the cell membrane and its signal intensity can be considered as directly proportional to quantity of cellular DNA. Cells whose cell membranes are damaged or have changed permeability are counted as dead ones. The assay was performed by seeding the cells of different cell lines at a density of 3000-7500 cells/well (in200µl medium) in culture plates followed by incubation for 24h at 37 °C in humidified 5%CO2/95% air atmospheric conditions. The medium was removed; the compounds were added to the plates at 10 µM concentrations (in 0.1% DMSO) in triplicates, followed by incubation for 48h. DMSO (0.1%) was used as control. After incubation, medium was removed followed by the addition of PI (25 µl, 50µg/mL in water/medium) to each well of the plates. At -80 °C, the plates were allowed to freeze for 24 h, followed by thawing at 25 °C. A fluorometer (Polar-Star BMG Tech) was used to record the readings at excitation and emission wavelengths of 530 and 620 nm for each well. The percentage cytotoxicity of compounds was calculated using the following formula:

$$\%\ \text{Cytotoxicity} = \frac{A_C - A_{TC}}{A_C} \times 100$$

Where A*TC*= Absorbance of treated cells and AC= Absorbance of control. Erlotinib was used as positive control in the assay.

4.2.3. Tubulin polymerization assay

The activity of compounds on tubulin polymerization was investigated by Tubulin Polymerization Assay Kit (Cytoskeleton Inc., Denver, CO, USA), which works via fluorescent reporter enhancement. The fluorescence of compounds (dissolved in DMSO at 5 and 25 µM concentration) was recorded in triplicates using FLUO star OPTIMA. Docetaxel and vincristine (Apoteket AB, Sweden) served as positive stabilizing and destabilizing controls. Both were used at 3 µM concentration in PBS.

4.3. Molecular Docking

The crystal structure of tubulin-CA4 complex (PDB code: 5LYJ) was downloaded from the Protein Data Bank. Structures of compounds **10a** and **13d** were drawn and optimized using MarvinSketch and Avogadro molecular editors. The protein was prepared using Autodock tools where the co-crystallized combretastatin A-4 and water molecules were removed then kollman charges and polar hydrogens were added. The grid coordinates for tubulin were set to 12.115x58.515x37.709 for x, y and z axes, respectively with grid dimensions of 80x80x80. Autodock vina was used for molecular docking and the best docking poses were visualized using Discovery Studio Visualizer.

Table S1. Binding affinities and amino acid interactions of **CA-4**, **10a** and **13d** in the colchicine binding site of tubulin

Compound	Binding affinity (Kcal/mol)	Amino acids
**CA-4**	-6.6	Leu242, Ala250, Leu255, Met259, Val181, Asn258, Lys352, Ala316, Ala354, Cys241, Val238, Ile318, Leu248
**10a**	-7.3	Leu242, Ala250, Leu255, Met259, Val181, Lys352,

		Ala316, Ala354, Cys241, Val238, Leu248, Leu252, Tyr202
**13d**	-7.2	Leu242, Ala250, Leu255, Met259, Val181, Lys352, Ala316, Cys241, Leu248, Tyr202

4.4. ADME prediction

The physicochemical and pharmacokinetic parameters of **10a** were predicted using the SwissADME tool (http://www.swissadme.ch/index.php). Lipophilicity was estimated through five independent models: WLOGP, XLOGP3, MLOGP, iLOGP, and SILICOS-IT. The arithmetic mean of these models was calculated to provide a consensus log Po/w value. The bioavailability radar illustrates six different physicochemical properties: size, polarity, lipophilicity, solubility, flexibility, and saturation.

Table S2. Physicochemical Properties of compound **10a**

Physicochemical Properties	
Formula	$C_{24}H_{19}N_3O_2S_3$
Molecular weight	477.62 g/mol
Number of heavy atoms	32
Number of aromatic heavy atoms	22
Fraction Csp3	0.08
Number of rotatable bonds	9
Number of H-bond acceptors	4
Number of H-bond donors	1
Molar Refractivity	133.55
TPSA	153.73 Å^2

Table S3. Lipophilicity Metrics of compound **10a**

Lipophilicity	
Log $P_{o/w}$ (iLOGP)	3.39
Log $P_{o/w}$ (XLOGP3)	6.23
Log $P_{o/w}$ (WLOGP)	5.90
Log $P_{o/w}$ (MLOGP)	2.53
Log $P_{o/w}$ (SILICOS-IT)	7.42
Consensus Log $P_{o/w}$	5.10

Table S4. Water Solubility Data for compound **10a**

Water Solubility	
Log *S* (ESOL)	-6.64
Solubility	1.09e-04 mg/ml ; 2.29e-07 mol/l
Class	Poorly soluble
Log *S* (Ali)	-9.24
Solubility	2.72e-07 mg/ml ; 5.70e-10 mol/l
Class	Poorly soluble
Log *S* (SILICOS-IT)	-8.53
Solubility	1.41e-06 mg/ml ; 2.95e-09 mol/l
Class	Poorly soluble

Table S5. Pharmacokinetics Profile of compound **10a**

Pharmacokinetics	
GI absorption	Low
BBB permeant	No
P-gp substrate	No
CYP1A2 inhibitor	No
CYP2C19 inhibitor	Yes
CYP2C9 inhibitor	Yes
CYP2D6 inhibitor	No
CYP3A4 inhibitor	Yes

Log K_p (skin permeation)	-4.79 cm/s

Table S6. Drug likeness Evaluation of compound **10a**

Drug likeness	
Lipinski	Yes; 0 violation
Ghose	No; 2 violations: WLOGP>5.6, MR>130
Veber	No; 1 violation: TPSA>140
Egan	No; 2 violations: WLOGP>5.88, TPSA>131.6
Muegge	No; 2 violations: XLOGP3>5, TPSA>150
Bioavailability Score	0.55

Table S7. Medicinal Chemistry Analysis of compound **10a**

Medicinal Chemistry	
Brenk	1 alert: michael_acceptor_1
Lead likeness	No; 3 violations: MW>350, Rotors>7, XLOGP3>3.5
Synthetic accessibility	3.94

4.5. Statistical analysis

Computerized Prism 5 program was used to statistically analyzed data using one-way ANOVA test followed by Tukey's as post ANOVA for multiple comparison at P ≤.05. Data were presented as mean ± SEM.

Raw Data

Average of Relative viability of cells (%)

Conc.(µM)	HCT-116	MCF-7	MDAMB-231	PC-3
**13c**				
100	16.1	21.7	19.6	28.8
50	29.9	36.7	38.9	42.1
25	42.2	51.6	50.1	54.6
12.5	51.3	68.1	54.8	67.7
6.25	73.1	83.0	71.6	87.9
3.125	92.6	96.3	93.3	99.1
1.56	100	100	100	100
**10h**				
100	20.2	23.6	21.6	36.5
50	39.4	37.1	26.7	55.8
25	52.8	50.2	37.1	72.4
12.5	69.3	53.9	47.4	93.5
6.25	79.5	76.5	68.5	100
3.125	97.6	94.8	85.7	100
1.56	100	100	100	100
**13b**				
100	14.3	8.2	9.2	17.1
50	27.4	14.7	17.4	25.3
25	39.5	22.6	26.3	33.7
12.5	53.2	39.3	34.6	45.8
6.25	73.3	57.1	60.5	69.2
3.125	92.7	83.5	68.9	86.4
1.56	100	97.8	91.7	100
**10m**				
100	28.4	22.3	24.8	36.8
50	43.7	28.4	32.3	46.7
25	54.1	37.2	43.7	57.4
12.5	67.3	53.5	55.2	70.2
6.25	87.4	72.7	68.5	84.1
3.125	99.8	91.6	87.1	98.5
1.56	100	100	100	100
**13d**				
100	9.2	8.3	7.5	7.6
50	17.4	17.1	11.3	19.4
25	26.5	24.5	15.6	20.5
12.5	32.7	32.4	35.2	35.6
6.25	63.9	45.2	45.6	57.8
3.125	69.8	71.2	57.9	83.3
1.56	90.6	88.6	71.2	97.1
**10f**				

100	23.6	19.2	14.3	24.2
50	35.7	27.4	17.8	32.3
25	50.5	33.5	27.4	46.7
12.5	63.4	41.9	38.7	58.2
6.25	71.1	68.3	63.2	70.5
3.125	90.2	84.7	79.5	91.8
1.56	100	100	96.1	100
		**10d**		
100	22.4	8.1	13.9	18.7
50	29.7	20.4	16.9	27.9
25	41.9	22.6	25.8	36.4
12.5	50.2	39.2	31.6	52.3
6.25	73.6	57.3	60.4	69.6
3.125	91.5	84.1	69.3	86.5
1.56	100	97.5	89.2	100
		**10o**		
100	7.8	7.2	4.5	14.3
50	15.2	11.9	10.2	23.7
25	22.7	20.3	17.6	31.6
12.5	24.5	29.7	26.7	48.2
6.25	51.6	45.1	37.3	61.1
3.125	66.5	61.6	47.1	87.4
1.56	89.1	73.4	61.2	99.3
		**10a**		
100	8.7	5.2	7.4	9.3
50	16.9	10.4	14.3	17.2
25	25.4	16.7	21.2	24.5
12.5	31.5	24.3	34.1	32.6
6.25	60.1	42.8	39.1	52.2
3.125	69.8	53.3	65.5	70.4
1.56	89.2	67.3	87.6	88.7

Tubulin polymerization Enzyme Assay

Researcher: Prof Dr. Bahaa

Type of analysis: Tubulin polymerization Enzyme assay

Tested Cell line: No cell lines used or any biological homogenate, the target compound activity against the enzyme were measured directly.

Kit Catalog no: ....

Reader: Tecan spark Multimode Plate Reader

Ex. 360 nm and Em. 450 nm.

Solvent: DMSO

No of samples: 10 samples

Samples code: 522, 528, 529, 530, 532, 533, 534, 535, 536, 537, Combretastatin(CA4)

Final results:

No	Compound	M.Wt	Tub. (IC_{50})	
	code	(g/mol)	IC_{50}±SD (µg/ml)	IC_{50} (µM) ≡ (µmol/L)
1	22	415.54	1.53±0.06	3.68±0.14
2	28	507.64	7.77±0.25	15.30±0.49
3	29	512.06	2.88±0.09	5.62±0.17
4	30	546.50	32.45±2.07	59.37±3.78
5	32	526.08	17.63±0.64	33.45±1.21
6	33	542.08	11.14±0.41	20.55±0.75
7	34	491.64	23.48±0.86	47.75±1.74
8	35	526.08	7.331±0.27	13.93±0.51
9	36	509.63	4.039±0.15	7.92±0.29
10	37	505.67	8.075±0.3	15.96±0.59
11	CA4	334.4	2.786±0.1	8.33±0.29

Fully detailed results:

Tub. enzyme inhibition assay												
	Conc.		Optical density			Temperature						
code	conc	Log Conc.	RFU2	RFU1	ΔRFU	T2	T1	ΔT	slope	%inhibition	K. Activity	IC50 (μg/ml)
522	100	2	3686	0	3686	30	0	30	948.4	87	15.546	1.53± 0.06
	10	1	8966	0	8966	30	0	30	948.4	68.5	37.815	
	1	0	14954	0	14954	30	0	30	948.4	47.4	63.07	
	0.1	-1	22472	0	22472	30	0	30	948.4	21	94.779	
	0.01	-2	26528	0	26528	30	0	30	948.4	6.76	111.89	
(Tub.control)	Non	Non	28453	0	28453	30	0	30	948.4	0	120	
code	conc	Log Conc.	RFU2	RFU1	ΔRFU	T2	T1	ΔT	slope	%inhibition	K. Activity	
528	100	2	4741	0	4741	30	0	30	948.4	83.3	19.996	7.77± 0.25
	10	1	15362	0	15362	30	0	30	948.4	46	64.791	
	1	0	22643	0	22643	30	0	30	948.4	20.4	95.5	
	0.1	-1	25722	0	25722	30	0	30	948.4	9.6	108.49	
	0.01	-2	27721	0	27721	30	0	30	948.4	2.57	116.92	
(Tub. Control)	Non	Non	28453	0	28453	30	0	30	948.4	0	120	
code	conc	Log Conc.	RFU2	RFU1	ΔRFU	T2	T1	ΔT	slope	%inhibition	K. Activity	
529	100	2	3529	0	3529	30	0	30	948.4	87.6	14.884	2.88± 0.09
	10	1	11707	0	11707	30	0	30	948.4	58.9	49.376	
	1	0	18537	0	18537	30	0	30	948.4	34.8	78.182	
	0.1	-1	24026	0	24026	30	0	30	948.4	15.6	101.33	
	0.01	-2	26964	0	26964	30	0	30	948.4	5.23	113.72	
(control)	Non	Non	28453	0	28453	30	0	30	948.4	0	120	
code	conc	Log Conc.	RFU2	RFU1	ΔRFU	T2	T1	ΔT	slope	%inhibition	K. Activity	32.45 ±2.07
530	100	2	8401	0	8401	30	0	30	948.4	70.5	35.432	

	10	1	19254	0	19254	30	0	30	948.4	32.3	81.206	
	1	0	24096	0	24096	30	0	30	948.4	15.3	101.63	
	0.1	-1	26961	0	26961	30	0	30	948.4	5.24	113.71	
	0.01	-2	28266	0	28266	30	0	30	948.4	0.65	119.22	
**(Tub. Control)**	Non	Non	28453	0	28453	30	0	30	948.4	0	120	
**code**	**conc**	**Log Conc.**	**RFU2**	**RFU1**	**ΔRFU**	**T2**	**T1**	**ΔT**	**slope**	**%inhibition**	**K. Activity**	
	100	2	7929	0	7929	30	0	30	948.4	72.1	33.442	
	10	1	16694	0	16694	30	0	30	948.4	41.3	70.409	
**532**	1	0	22973	0	22973	30	0	30	948.4	19.3	96.892	**17.63 ±0.64**
	0.1	-1	26881	0	26881	30	0	30	948.4	5.52	113.37	
	0.01	-2	28021	0	28021	30	0	30	948.4	1.51	118.18	
**(Tub. Control)**	Non	Non	28453	0	28453	30	0	30	948.4	0	120	
**code**	**conc**	**Log Conc.**	**RFU2**	**RFU1**	**ΔRFU**	**T2**	**T1**	**ΔT**	**slope**	**%inhibition**	**K. Activity**	
	100	2	6971	0	6971	30	0	30	948.4	75.5	29.401	
	10	1	15453	0	15453	30	0	30	948.4	45.7	65.175	
**533**	1	0	22032	0	22032	30	0	30	948.4	22.6	92.923	**11.14 ±0.41**
	0.1	-1	26439	0	26439	30	0	30	948.4	7.08	111.51	
	0.01	-2	27881	0	27881	30	0	30	948.4	2.01	117.59	
**(Tub. Control)**	Non	Non	28453	0	28453	30	0	30	948.4	0	120	
**code**	**conc**	**Log Conc.**	**RFU2**	**RFU1**	**ΔRFU**	**T2**	**T1**	**ΔT**	**slope**	**%inhibition**	**K. Activity**	
	100	2	7992	0	7992	30	0	30	948.4	71.9	33.707	
	10	1	18061	0	18061	30	0	30	948.4	36.5	76.175	
**534**	1	0	23765	0	23765	30	0	30	948.4	16.5	100.23	**23.48 ±0.86**
	0.1	-1	27003	0	27003	30	0	30	948.4	5.09	113.89	
	0.01	-2	28372	0	28372	30	0	30	948.4	0.28	119.66	
**(Tub. Control)**	Non	Non	28453	0	28453	30	0	30	948.4	0	120	
**code**	**conc**	**Log Conc.**	**RFU2**	**RFU1**	**ΔRFU**	**T2**	**T1**	**ΔT**	**slope**	**%inhibition**	**K. Activity**	**7.331 ±0.27**

**535**	100	2	5829	0	5829	30	0	30	948.4	79.5	24.585	
	10	1	14424	0	14424	30	0	30	948.4	49.3	60.835	
	1	0	21441	0	21441	30	0	30	948.4	24.6	90.43	
	0.1	-1	25357	0	25357	30	0	30	948.4	10.9	106.95	
	0.01	-2	27926	0	27926	30	0	30	948.4	1.85	117.78	
**(Tub. Control))**	Non	Non	28453	0	28453	30	0	30	948.4	0	120	
**code**	**conc**	**Log Conc.**	**RFU2**	**RFU1**	**ΔRFU**	**T2**	**T1**	**ΔT**	**slope**	**%inhibition**	**K. Activity**	
**536**	100	2	5361	0	5361	30	0	30	948.4	81.2	22.611	**4.039 ±0.15**
	10	1	11943	0	11943	30	0	30	948.4	58	50.371	
	1	0	19927	0	19927	30	0	30	948.4	30	84.045	
	0.1	-1	23671	0	23671	30	0	30	948.4	16.8	99.836	
	0.01	-2	26754	0	26754	30	0	30	948.4	5.97	112.84	
**(Tub. Control)**	Non	Non	28453	0	28453	30	0	30	948.4	0	120	
**code**	**conc**	**Log Conc.**	**RFU2**	**RFU1**	**ΔRFU**	**T2**	**T1**	**ΔT**	**slope**	**%inhibition**	**K. Activity**	
**537**	100	2	6617	0	6617	30	0	30	948.4	76.7	27.908	**8.075 ±0.3**
	10	1	14259	0	14259	30	0	30	948.4	49.9	60.139	
	1	0	21038	0	21038	30	0	30	948.4	26.1	88.73	
	0.1	-1	25909	0	25909	30	0	30	948.4	8.94	109.27	
	0.01	-2	27881	0	27881	30	0	30	948.4	2.01	117.59	
**(Tub. Control)**	Non	Non	28453	0	28453	30	0	30	948.4	0	120	
**code**	**conc**	**Log Conc.**	**RFU2**	**RFU1**	**ΔRFU**	**T2**	**T1**	**ΔT**	**slope**	**%inhibition**	**K. Activity**	
**CA4**	100	2	4492	**0**	4492	30	0	30	948.4	84.2	18.946	**2.786 ±0.1**
	10	1	11657	**0**	11657	30	0	30	948.4	59	49.165	
	1	0	18535	**0**	18535	30	0	30	948.4	34.9	78.174	
	0.1	-1	22427	**0**	22427	30	0	30	948.4	21.2	94.589	
	0.01	-2	25976	**0**	25976	30	0	30	948.4	8.7	109.56	
**(Tub. Control)**	Non	Non	28453	**0**	28453	30	0	30	948.4	0	120	

#(Tub. Control)→referred to Control (Kit without any testing compound) and represented full activity or 100% activity.

#%Inh → referred to the inhibition percentage caused by the tested compound.

# T1 and T2 → the starting and ending time and the difference (ΔT=30 min.) represented the reaction time between enzyme kit and tested compound.

# RFU1 and RFU2 → each one of them represented the relative fluorescence (or by the mean Optical density) unit at time T1 and T2 and the difference (Δ RFU) represents the total relative fluorescence after reaction completion (30min.).

# K. activity → referred to Kinetic activity of the tested compound after reaction time completion.

# IC_{50} →referred to Half maximal enzyme activity concentration.

Note: For IC_{50} calculation use the straight-line equation and let the value of Y equals 50 and calculate the value of x, the antilog x value equals the value of IC_{50}

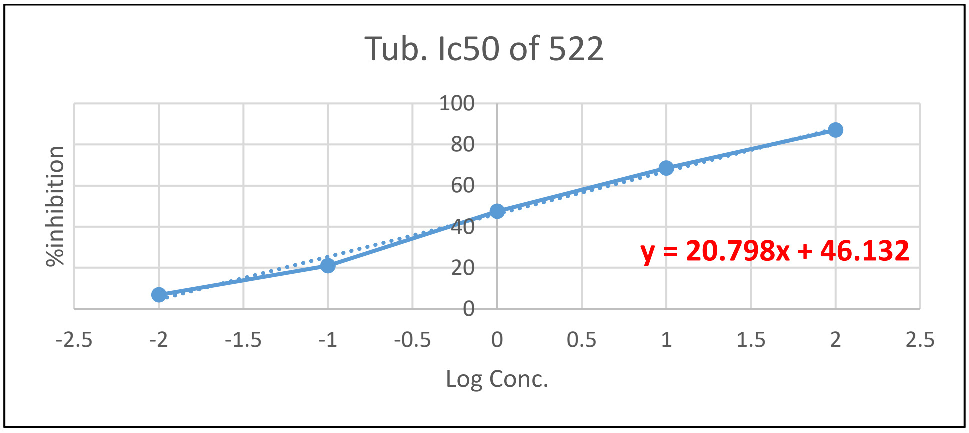

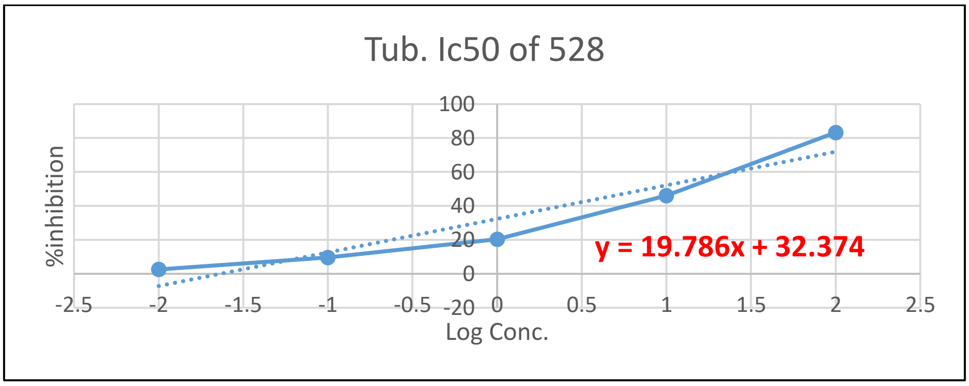

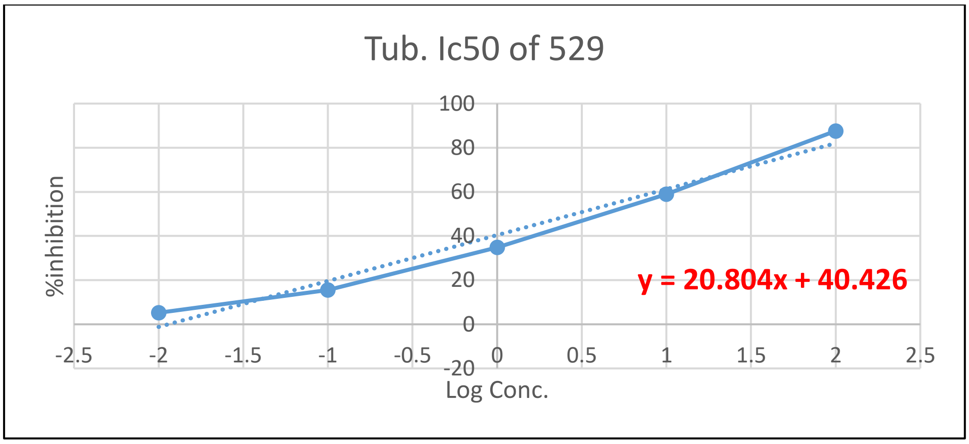
Tub. Ic50 of 529
%inhibition
Log Conc.
y = 20.804x + 40.426
100
80
60
40
20
0
-20
-2.5
-2
-1.5
-1
-0.5
0
0.5
1
1.5
2
2.5

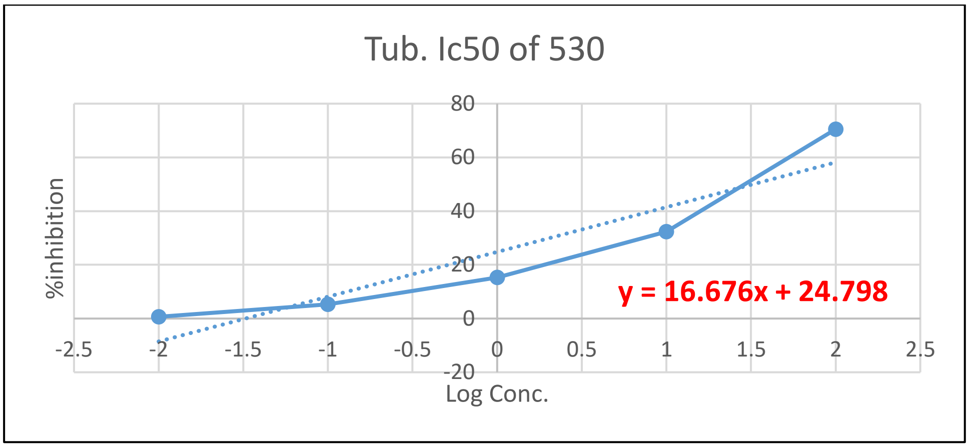
Tub. Ic50 of 530
%inhibition
Log Conc.
y = 16.676x + 24.798
80
60
40
20
0
-20
-2.5
-2
-1.5
-1
-0.5
0
0.5
1
1.5
2
2.5

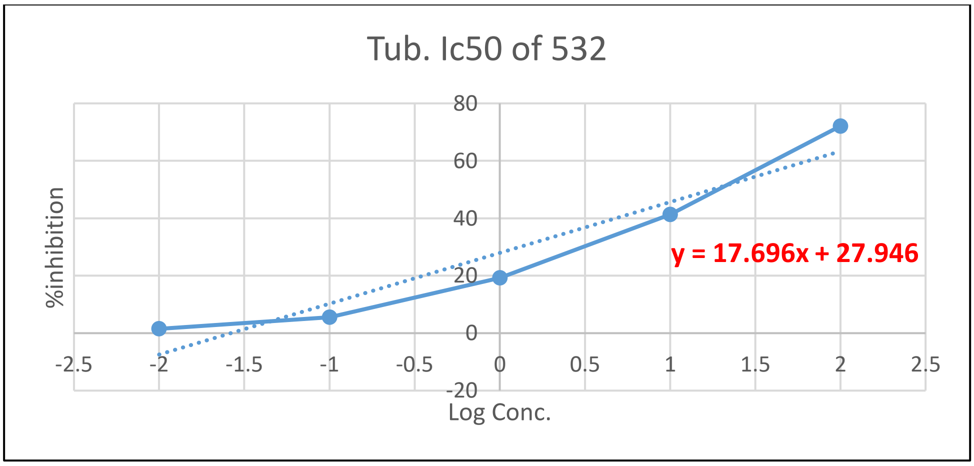
Tub. Ic50 of 532
%inhibition
Log Conc.
y = 17.696x + 27.946
80
60
40
20
0
-20
-2.5
-2
-1.5
-1
-0.5
0
0.5
1
1.5
2
2.5

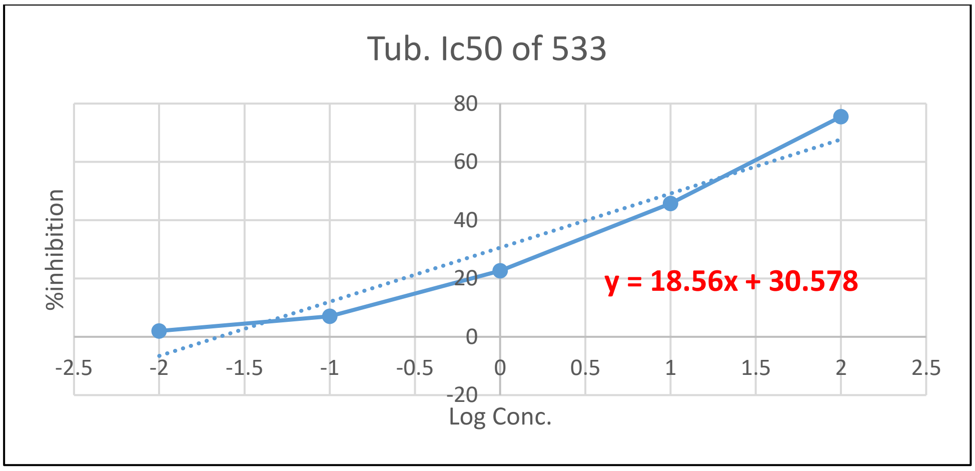
Tub. Ic50 of 533
%inhibition
Log Conc.
y = 18.56x + 30.578

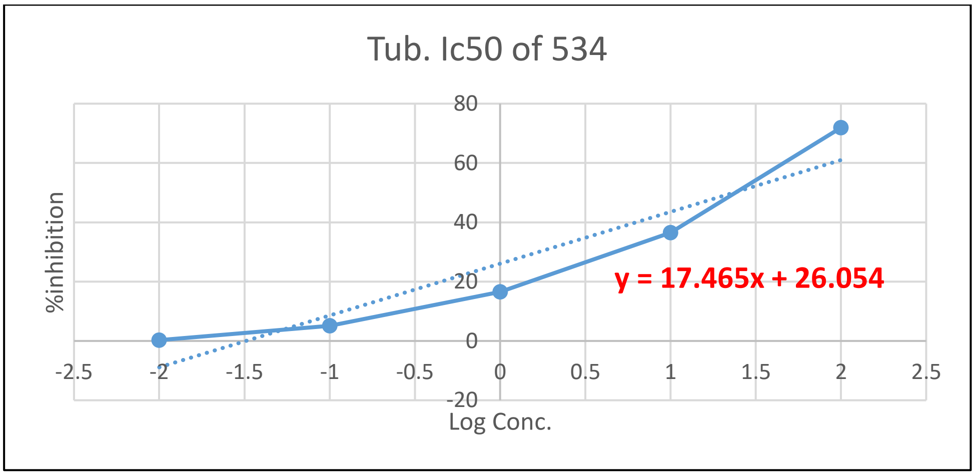
Tub. Ic50 of 534
%inhibition
Log Conc.
y = 17.465x + 26.054

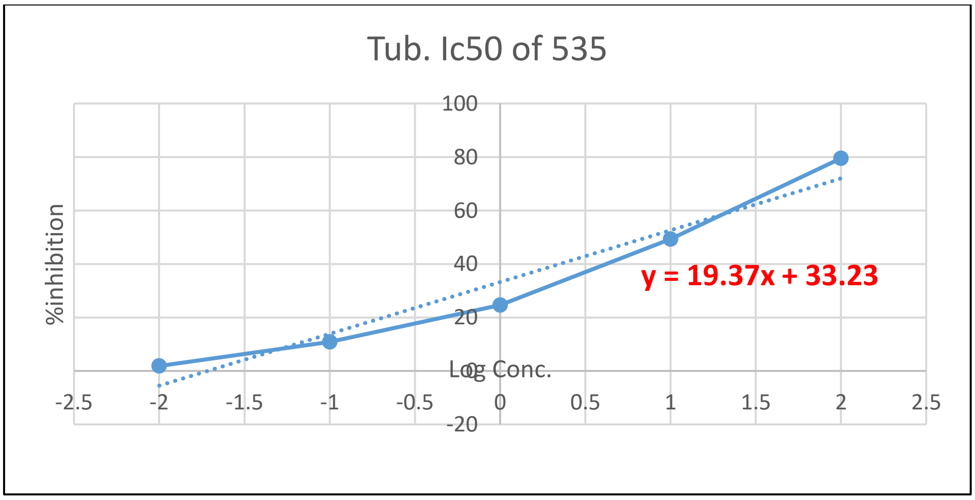
Tub. Ic50 of 535
%inhibition
Log Conc.
y = 19.37x + 33.23

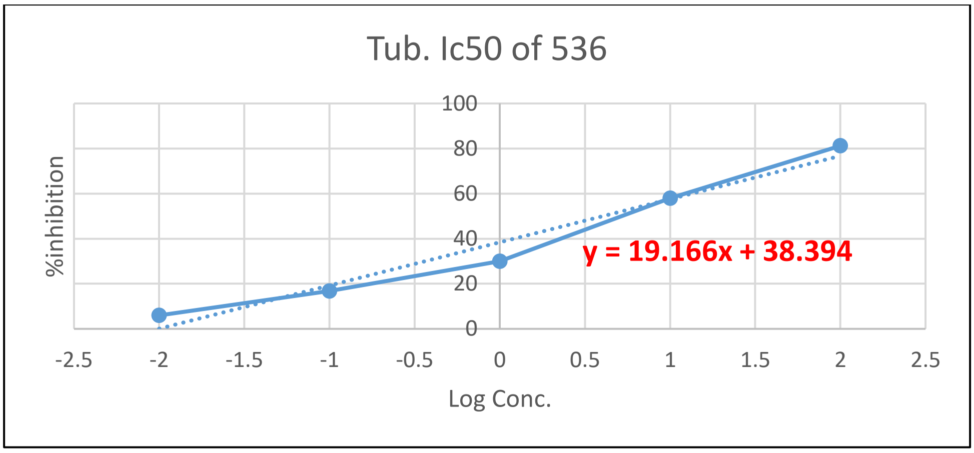
Tub. Ic50 of 536
%inhibition
Log Conc.
y = 19.166x + 38.394
100
80
60
40
20
0
-2.5
-2
-1.5
-1
-0.5
0
0.5
1
1.5
2
2.5

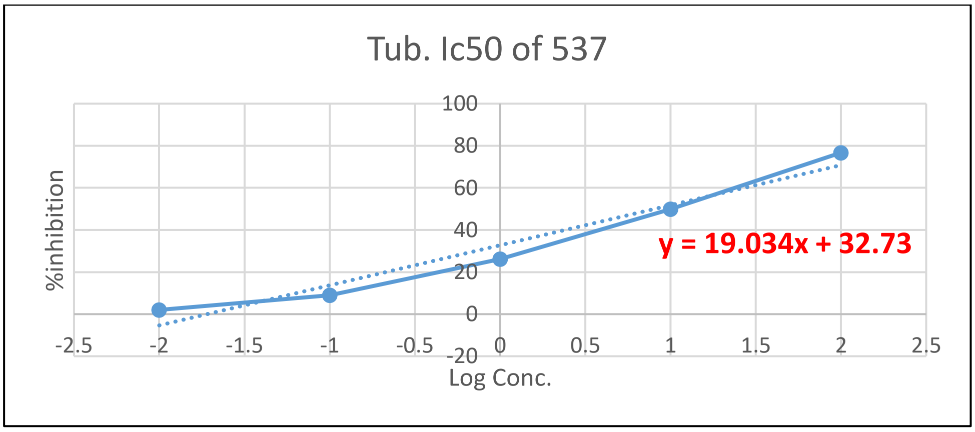
Tub. Ic50 of 537
%inhibition
Log Conc.
y = 19.034x + 32.73
100
80
60
40
20
0
-20
-2.5
-2
-1.5
-1
-0.5
0
0.5
1
1.5
2
2.5

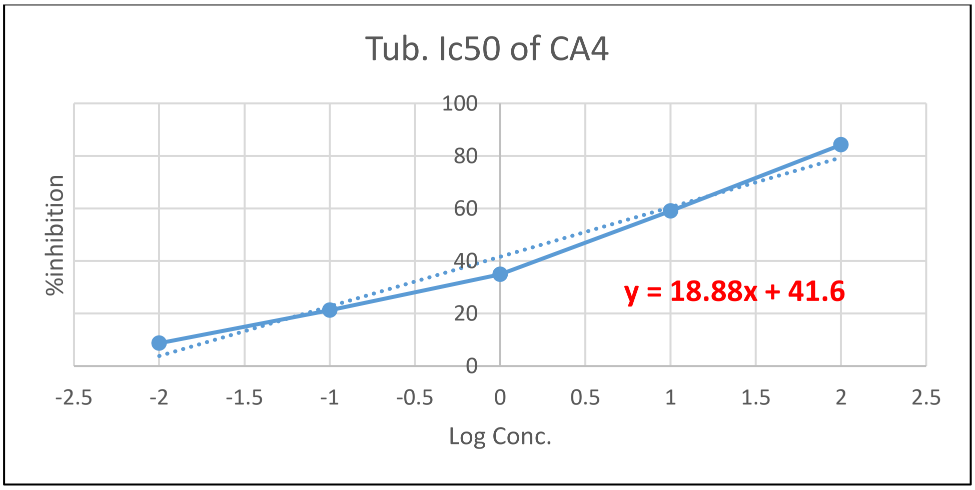
Tub. Ic50 of CA4
%inhibition
Log Conc.
y = 18.88x + 41.6
100
80
60
40
20
0
-2.5
-2
-1.5
-1
-0.5
0
0.5
1
1.5
2
2.5

Tub

code	IC50	conc	log	%inh	T2	T1	ΔT	RFU2	RFU1	ΔRFU	slope	K.Activity
**YB-EGY-519**		100	2	78.7	30	0	30	8329	0	8329	1301	25.608
		50	1.7	62.4	30	0	30	14661	0	14661	1301	45.076
		10	1	32.4	30	0	30	26391	0	26391	1301	81.141
		1	0	16.6	30	0	30	32555	0	32555	1301	100.09
		0.1	-1	5.68	30	0	30	36812	0	36812	1301	113.18
EC				0	30	0	30	39025	0	39025	1301	120

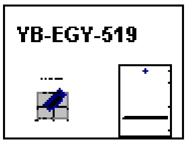

code	IC50	conc	log	%inh	T2	T1	ΔT	RFU2	RFU1	ΔRFU	slope	K.Activity
**YB-EGY-520**		100	2	92.3	30	0	30	2991	0	2991	1301	9.196
		50	1.7	76.9	30	0	30	9026	0	9026	1301	27.751
		10	1	54.2	30	0	30	17862	0	17862	1301	54.918
		1	0	32.3	30	0	30	26428	0	26428	1301	81.254
		0.1	-1	15.2	30	0	30	33079	0	33079	1301	101.7
EC				0	30	0	30	39025	0	39025	1301	120

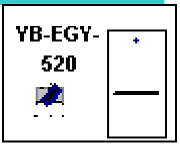

code	IC50	conc	log	%inh	T2	T1	ΔT	RFU2	RFU1	ΔRFU	slope	K.Activity
YB-EGY-521		100	2	90.8	30	0	30	3576	0	3576	1301	10.995
		50	1.7	71.2	30	0	30	11237	0	11237	1301	34.549
		10	1	48.8	30	0	30	19982	0	19982	1301	61.436
		1	0	27.4	30	0	30	28334	0	28334	1301	87.115
		0.1	-1	6.12	30	0	30	36643	0	36643	1301	112.66
EC				0	30	0	30	39025	0	39025	1301	120

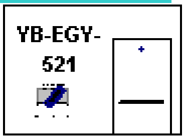

code	IC50	conc	log	%inh	T2	T1	ΔT	RFU2	RFU1	ΔRFU	slope	K.Activity
**YB-EGY-523**		100	2	75.9	30	0	30	9422	0	9422	1301	28.968
		50	1.7	41.7	30	0	30	22756	0	22756	1301	69.965
		10	1	26.3	30	0	30	28753	0	28753	1301	88.403
		1	0	16.4	30	0	30	32642	0	32642	1301	100.36
		0.1	-1	7.07	30	0	30	36272	0	36272	1301	111.52
EC				0	30	0	30	39025	0	39025	1301	120

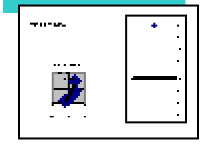

code	IC50	conc	log	%inh	T2	T1	ΔT	RFU2	RFU1	ΔRFU	slope	K.Activity
YB-EGY-524		100	2	92.7	30	0	30	2849	0	2849	1301	8.7594
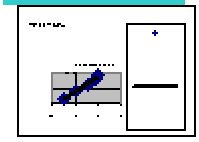		50	1.7	78.9	30	0	30	8253	0	8253	1301	25.374
		10	1	60.5	30	0	30	15414	0	15414	1301	47.391
		1	0	39.4	30	0	30	23649	0	23649	1301	72.71
		0.1	-1	16.5	30	0	30	32585	0	32585	1301	100.18
EC				0	30	0	30	39025	0	39025	1301	120

code	IC50	conc	log	%inh	T2	T1	ΔT	RFU2	RFU1	ΔRFU	slope	K.Activity
YB-EGY-525		100	2	88.7	30	0	30	4416	0	4416	1301	13.577
		50	1.7	64.5	30	0	30	13865	0	13865	1301	42.629
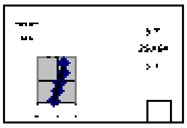		10	1	41.4	30	0	30	22876	0	22876	1301	70.334
		1	0	23.9	30	0	30	29691	0	29691	1301	91.287
		0.1	-1	5.79	30	0	30	36771	0	36771	1301	113.05
EC				0	30	0	30	39025	0	39025	1301	120

code	IC50	conc	log	%inh	T2	T1	ΔT	RFU2	RFU1	ΔRFU	slope	K.Activity
YB-EGY-526		100	2	83	30	0	30	6643	0	6643	1301	20.424
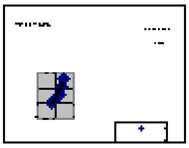		50	1.7	52.5	30	0	30	18539	0	18539	1301	56.999
		10	1	25.6	30	0	30	29027	0	29027	1301	89.245
		1	0	12.4	30	0	30	34181	0	34181	1301	105.09
		0.1	-1	4.47	30	0	30	37286	0	37286	1301	114.64
EC				0	30	0	30	39025	0	39025	1301	120

code	IC50	conc	log	%inh	T2	T1	ΔT	RFU2	RFU1	ΔRFU	slope	K.Activity
YB-EGY-527		100	2	91.1	30	0	30	3461	0	3461	1301	10.641
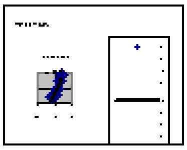		50	1.7	71.7	30	0	30	11027	0	11027	1301	33.903
		10	1	45.2	30	0	30	21376	0	21376	1301	65.722
		1	0	26.6	30	0	30	28664	0	28664	1301	88.129
		0.1	-1	17.5	30	0	30	32182	0	32182	1301	98.945
EC				0	30	0	30	39025	0	39025	1301	120

code	IC50	conc	log	%inh	T2	T1	ΔT	RFU2	RFU1	ΔRFU	slope	K.Activity
YB-EGY-531		100	2	84.5	30	0	30	6052	0	6052	1301	18.607
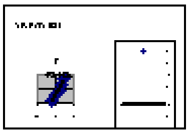		50	1.7	65.5	30	0	30	13467	0	13467	1301	41.405
		10	1	44.8	30	0	30	21554	0	21554	1301	66.269

		1	0	26.6	30	0	30	28665	0	28665	1301	88.132
		0.1	-1	9.85	30	0	30	35187	0	35187	1301	108.18
EC				0	30	0	30	39025	0	39025	1301	120

code	IC50	conc	log	%inh	T2	T1	ΔT	RFU2	RFU1	ΔRFU	slope	K.Activity
**YB-EGY-538**		100	2	90.9	30	0	30	3566	0	3566	1301	10.964
		50	1.7	76.2	30	0	30	9271	0	9271	1301	28.504
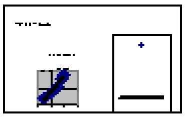		10	1	54.8	30	0	30	17646	0	17646	1301	54.254
		1	0	34	30	0	30	25751	0	25751	1301	79.173
		0.1	-1	16	30	0	30	32775	0	32775	1301	100.77
EC				0	30	0	30	39025	0	39025	1301	120

YB-EGY-519	y = 23.623x + 21.674
YB-EGY-520	y = 25.008x + 35.696
YB-EGY-521	y = 26.758x + 29.079
YB-EGY-523	y = 19.194x + 19.265
YB-EGY-524	y = 24.442x + 39.515
YB-EGY-525	y = 25.464x + 26.015
YB-EGY-526	y = 23.517x + 18.202
YB-EGY-527	y = 23.619x + 32.97
YB-EGY-531	y = 23.443x + 28.891
YB-EGY-538	y = 24.202x + 36.485

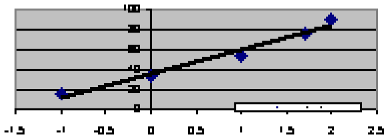

Supplement: Supplementary file 1 [file DataSheet1.pdf]
